# The *Bacillus* BioBrick Box: generation and evaluation of essential genetic building blocks for standardized work with *Bacillus subtilis*

**DOI:** 10.1186/1754-1611-7-29

**Published:** 2013-12-02

**Authors:** Jara Radeck, Korinna Kraft, Julia Bartels, Tamara Cikovic, Franziska Dürr, Jennifer Emenegger, Simon Kelterborn, Christopher Sauer, Georg Fritz, Susanne Gebhard, Thorsten Mascher

**Affiliations:** 1 Department Biology I, AG Synthetic Microbiology, Ludwig-Maximilians-Universität München, Grosshaderner Str. 2-4, D-82152 Planegg-Martinsried, Germany; 2 Ludwig-Maximilians-University Munich, Arnold Sommerfeld Center for Theoretical Physics, Theresienstr. 37, D-80333 München, Germany; 3 Present affiliation: Institute of Cell and Molecular Biosciences, Newcastle University, Centre for Bacterial Cell Biology, Richardson Road, NE2 4AX Newcastle upon Tyne, UK

**Keywords:** *Bacillus subtilis*, BioBrick standard, iGEM, *lux*, Luminescence, Epitope tag, Integrative vector, Plasmid, Inducible promoter

## Abstract

**Background:**

Standardized and well-characterized genetic building blocks are a prerequisite for the convenient and reproducible assembly of novel genetic modules and devices. While numerous standardized parts exist for *Escherichia coli*, such tools are still missing for the Gram-positive model organism *Bacillus subtilis*. The goal of this study was to develop and thoroughly evaluate such a genetic toolbox.

**Results:**

We developed five BioBrick-compatible integrative *B. subtilis* vectors by deleting unnecessary parts and removing forbidden restriction sites to allow cloning in BioBrick (RFC10) standard. Three empty backbone vectors with compatible resistance markers and integration sites were generated, allowing the stable chromosomal integration and combination of up to three different devices in one strain. In addition, two integrative reporter vectors, based on the *lacZ* and *luxABCDE* cassettes, were BioBrick-adjusted, to enable β-galactosidase and luciferase reporter assays, respectively. Four constitutive and two inducible promoters were thoroughly characterized by quantitative, time-resolved measurements. Together, these promoters cover a range of more than three orders of magnitude in promoter strength, thereby allowing a fine-tuned adjustment of cellular protein amounts. Finally, the *Bacillus* BioBrick Box also provides five widely used epitope tags (FLAG, His_10_, cMyc, HA, StrepII), which can be translationally fused N- or C-terminally to any protein of choice.

**Conclusion:**

Our genetic toolbox contains three compatible empty integration vectors, two reporter vectors and a set of six promoters, two of them inducible. Furthermore, five different epitope tags offer convenient protein handling and detection. All parts adhere to the BioBrick standard and hence enable standardized work with *B. subtilis*. We believe that our well-documented and carefully evaluated *Bacillus* BioBrick Box represents a very useful genetic tool kit, not only for the iGEM competition but any other BioBrick-based project in *B. subtilis*.

## Introduction

One core aspect of synthetic biology projects that sets them apart from “classical” genetic work is the application of engineering principles such as abstraction, modularity and standardization to assembly strategies and procedures. The characterization and standardization of reusable genetic building blocks is one of the prerequisites for the engineering approach of building complex synthetic biological systems [1]. Towards that end, the Registry of Standard Biological Parts (partsregistry, [2]) was founded by the Massachusetts Institute of Technology in 2003 as a repository for the *i*nternational *G*enetically *E*ngineered *M*achine competition (iGEM) and now maintains and distributes over ten thousand standardized biological parts that adhere to the BioBrick standard as described in the “request for comments 10” (RFC 10) [3]. Such standardized genetic parts – which have for example been successfully used for the construction of novel genetic circuitries, such as a bacterial camera or a push-on-push-off-switch [4,5] – not only significantly simplify devices assembly, but also increase the reproducibility of the resulting constructs [6].

While there are a number of other assembly techniques like Gibson assembly [7], Golden Gate shuffling [8,9] or MoClo [10], the BioBrick standard still plays a key role in the framework of the annual iGEM competition. Moreover, it is also very useful for any other lab, since it is based on standard type II restriction endonucleases used for routine cloning. While the use of such standardized parts and assembly strategies is organism-independent, there is nevertheless a need for specific parts that accommodate organism-specific requirements, such as G+C content, codon preference and different expression and/or regulatory signals.

The classical BioBrick standard allows the free combination of most parts, but does not work for translational fusions, e.g. for addition of *gfp* or epitope-tags to protein-coding sequences. For this purpose, a number of BioBrick-compatible adaptations were developed, as described in RFC 23 and RFC 25 [11,12]. In each case, the combination of parts is performed via standard restriction digests and subsequent ligations, preferably with vector backbones of different antibiotic resistances to allow the so-called 3A-assembly [13].

Currently, the vast majority of available parts in the Registry of Standard Biological Parts were designed for the Gram-negative model organism *Escherichia coli*, due to its central role in iGEM and other synthetic biology projects. For other organisms, such as the Gram-positive model organism *Bacillus subtilis*, the range of available BioBricks is still very limited, especially when looking for well-evaluated and reliable parts. This limitation is unfortunate, given the unique features, powerful genetics and biotechnological relevance of this bacterium.

*B. subtilis*, together with other *Bacillus* species, is one of the main producers of industrially relevant enzymes, such as proteases, amylases and lipases. Its excellent fermentation properties, the ability to efficiently secrete proteins and the lack of toxic by-products render it indispensable for the biotechnological industry [14]. *B. subtilis* is the by far best-characterized Gram-positive bacterium [15], due to its powerful genetics and advantages for industrial use. In addition, *B. subtilis* has also become a model organism for studying bacterial (multicellular) differentiation, because of its capability to form highly resistant endospores upon nutrient limitation [16-18]. Another transient differentiation strategy is to become naturally competent for genetic transformation by synthesizing the machinery necessary for DNA uptake [17,19,20]. The high efficiency of this process and its tight association with homologous recombination not only enables easy genetic manipulations of the chromosome, but has also led to the development of mostly integrative vectors for use in *B. subtilis*, even though replicative vectors can also be used [21]. The advantages of integrative vectors are their stable maintenance and hence also defined copy number inside the chromosome.

Based on all those features and differences, the 2012 LMU-Munich iGEM-team decided to develop and provide a set of essential genetic tools for the work with *B. subtilis*. First of all, empty compatible integrative vectors are needed that can carry any construct of choice. Second, well-evaluated promoters of different strength, both constitutive and inducible, are required to control gene expression levels. For their evaluation and as a tool for measuring the activity of other promoters, reporter vectors are also required. For that purpose, we chose the well-established *lacZ* reporter, encoding the β-galactosidase, as well as a luciferase reporter (*luxABCDE*), which we carefully evaluated for the first time. Moreover, a set of epitope tags is provided to enable the convenient detection and purification of proteins. The resulting toolbox presented here, called the *Bacillus* BioBrick Box, (Table 1) is now completely evaluated and freely available in two DNA repositories, the Registry of Standard Biological Parts [2] and the Bacillus Genetics Stock Center [22]. The vector sources and formatted GeneBank files are given in Table 2 and Additional file 1, respectively.

**Table 1:** **Overview of the *Bacillus *** **BioBrick Box including a summary of the features**

**BioBrick**	**Description and comments**^**a**^	**Reference of source**	**BGSC #**	**Registry #**
**Vectors**				
pBS1C	Empty vector, integration at *amyE*, amp^r^, cm^r^	pDG1662-derivative [23]	ECE257	BBa_K823023
pBS2E	Empty vector, integration at *lacA*, amp^r^, mls^r^	pAX01-derivative [24]	ECE258	BBa_K823027
pBS4S	Empty vector, integration at *thrC*, amp^r^, spec^r^	pDG1731-derivative [23]	ECE259	BBa_K823022
pBS1C*lacZ*	*lacZ*-reporter vector, integration at *amyE*, amp^r^, cm^r^	pAC6-derivative [25]	ECE260	BBa_K823021
pBS3C*lux*	*lux*-reporter vector, integration at *sacA*, amp^r^, cm^r^	pAH328-derivative [26]	ECE261	BBa_K823025
**Promoters**				
P_*veg*_	Very strong constitutive promoter	[27]	ECE262	BBa_K823003
P_*liaG*_	Constitutive promoter	[28]	ECE263	BBa_K823000
P_*lepA*_	Strong constitutive promoter	[29]	ECE264	BBa_K823002
J23101	Very weak constitutive promoter	BBa_J23101	ECE266	BBa_K823005
P_*liaI*_	Bacitracin-inducible promoter	[30,31]	ECE267	BBa_K823001
P_*xylA*_	Xylose-inducible promoter	[32]	ECE268	BBa_K823015
**Epitope-tags**				
His_10_	10xHis-tag	[33]	ECE269	BBa_K823037
FLAG	FLAG-tag	[34]	ECE270	BBa_K823034
StrepII	Streptactin-tag	[35,36]	ECE271	BBa_K823038
HA	HA-tag	[34,37]	ECE272	BBa_K823035
cMyc	cMyc-tag	[34,38]	ECE273	BBa_K823036

^a^amp^r^, ampicillin resistance; cm^r^, chloramphenicol resistance; kan^r^, kanamycin resistance; spc^r^, spectinomycin resistance; mls^r^, erythromycin-induced resistance to *m*acrolide, *l*incosamide and *s*treptogramin B-antibiotics (MLS).

**Table 2:** **Vector sources for the *Bacillus *** **BioBrick Box**

**Vector**	**Description**^***a***^	**Used for**	**Ref.**
pAC6	Vector for transcriptional promoter fusions to *lacZ*; integrates at *amyE*; cm^r^, amp^r^	pBS1C*lacZ*	[25]
pAH328	Vector for transcriptional promoter fusions to *luxABCDE* (luciferase); integrates at *sacA*; cm^r^, amp^r^	pBS3C*lux*	[26]
pDG1662	Empty vector, integrates at *amyE*, cm^r^, spc^r^, amp^r^	pBS1C	[23]
pDG1731	Empty vector; integrates at *thrC*, spc^r^, mls^r^, amp^r^	pBS4S	[23]
pAX01	Vector for xylose-dependent gene expression; integrates at *lacA*, mls^r^, amp^r^	pBS2E	[24]
pXT	Vector for xylose-inducible gene expression; integrates in *thrC*; spc^r^, amp^r^	P_*xylA*_	[39]
pSB1C3	Replicative *E. coli* vector, MCS features *rfp*-cassette; cm^r^	MCS	[40]
pGFPamy	Vector for transcriptional promoter fusions to *gfpmut1*; integrates at *amyE*; cm^r^, amp^r^	*gfpmut1*	[41]

^a^cm^r^, chloramphenicol resistance; kan^r^, kanamycin resistance; spc^r^, spectinomycin resistance; mls^r^, erythromycin-induced resistance to *m*acrolid-, *l*incosamid- and *s*treptogramin B-antibiotics (MLS); MCS, multiple cloning site.

## Results and discussion

### The vectors of the *Bacillus* BioBrick Box

**Figure 1:**
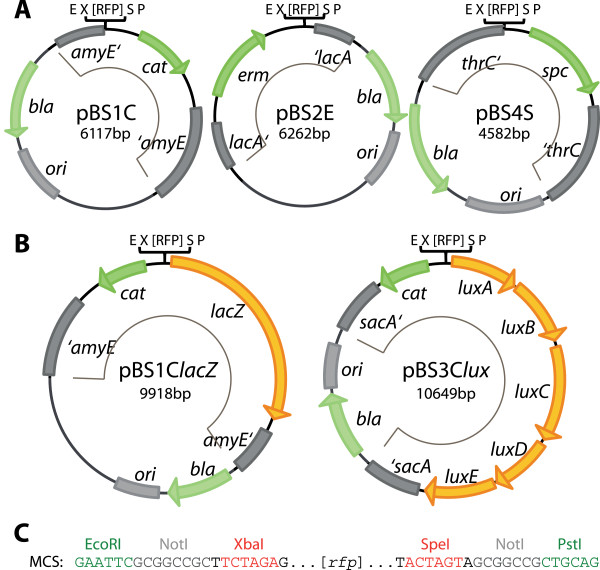
**Maps of the modified *Bacillus *BioBrick Box-vectors in BioBrick (RFC10) standard.** Resistance genes are indicated in green, reporter genes in orange and the origin of replication for *E. coli* as well as the integration loci are shown as gray boxes. The integrative part of the vectors is marked with a thin black line inside each vector map. The vector names are based on the following nomenclature: p = plasmid, BS = *B. subtilis*; the number refers to the integration locus: 1 = *amyE*, 2 = *lacA*, 3 = *sacA*, 4 = *thrC*; and the last letter codes for the resistance in *B. subtilis*: C = chloramphenicol (mediated by *cat*), E = MLS (mediated by *erm:* specifies resistance to *m*acrolide, *l*incosamide and *s*treptogramin B antibiotics if induced by erythromycin), S = spectinomycin (mediated by *spc*)). The addition in italics refers to the functional part of the reporter vectors: *lacZ* for β-galactosidase and *lux* for the *luxABCDE*-cassette encoding luciferase. **(A)** The three empty integrative vectors pBS1C, pBS2E and pBS4S contain different resistance genes (*cat*, *erm*, *spec*) and integration loci (*amyE*, *lacA*, *thrC*). This compatibility allows a combined usage in one single strain. They are derived from pDG1662, pAX01 and pDG1731, respectively. **(B)** Reporter vectors encoding β-galactosidase (*lacZ*) or luciferase (*luxABDCE*) as reporter genes. They integrate into *amyE* or *sacA*, respectively, and both mediate chloramphenicol resistance. All vectors harbor the RFC10-compatible multiple cloning site with an *rfp*-cassette, as shown in **(C)**. See text and material and methods for details on construction.

To allow the integration of new devices and modules into the chromosome of *B. subtilis*, we first constructed five vectors (see Table 1 and Figure 1) by modifying established *B. subtilis* vectors to comply with the BioBrick standard. As described in detail below, we chose the original *B. subtilis* vectors such that they harbor three different resistance cassettes and also compatible homology regions for integration into the *B. subtilis* chromosome. This way, all three vectors and derived plasmids can be combined in a single strain. For convenient cloning, they all contain the *bla* cassette for ampicillin resistance and an *E. coli* origin of replication (ori).

The original *B. subtilis* vectors pDG1662, pAX01, pDG1731 [23,24] were modified to generate “empty” vectors that lack promoters and reporter genes (Figure 1A). Their integrative part contains the flanking homology regions, a resistance cassette for selection in *B. subtilis* and the multiple cloning site (MCS), containing an *rfp*-cassette flanked by the restriction sites EcoRI, NotI, XbaI (upstream) and SpeI, NotI and PstI (downstream) (Figure 1C). They allow cloning in BioBrick standard [3] with selection for white colonies as a result of the removal of the *rfp*-insert, which – if still present – leads to formation of red colonies in *E. coli*. All unnecessary parts, such as promoters or additional resistance genes were deleted (see methods for details on construction). In all vectors the restriction sites interfering with the RFC 10 standard were removed. Moreover, for the empty vectors we also removed all AgeI and NgoMIV sites, thus allowing direct cloning in “Freiburg” standard [11] for translational fusions. Alignments with original vectors are provided in Additional file 2.

The three empty vectors, designated pBS1C, pBS2E and pBS4S, integrate into the *amyE*, *lacA* and *thrC* locus and harbor *cat*, *erm* and *spc* resistance cassettes, respectively (Table 1). The reporter vector pBS1C*lacZ* was derived from pAC6 [25] and contains the β-galactosidase reporter gene *lacZ* from *E. coli* with the *sacB* ribosome binding site (RBS) from *B. subtilis* downstream of the MCS. The luciferase-reporter vector pBS3C*lux*, which was derived from pAH328 [26], contains the *luxABCDE*-operon from *Photorabdus luminescence* with all RBSs adjusted for use in *B. subtilis* (Figure 1B). Both reporter vectors allow the measurement of promoter activities based on transcriptional fusions and mediate chloramphenicol resistance. They integrate into the *amyE* (pBS1C*lacZ*) and *sacA* (pBS3C*lux*) loci, respectively.

For transformation of *B. subtilis*, the vectors and derived plasmids need to be linearized at a unique ScaI site inside the *bla* gene. The only exception is pBS2E, which contains a second ScaI site within the integrative part. For this vector, BsaI or PciI can be used for linearization. The functionality of all empty vectors has been verified in a number of different projects (own unpublished results). The reporter vectors were subsequently used to evaluate the different promoters of the *Bacillus* BioBrick Box (see further below).

### Characterization of the *thrC* integration locus

The four integration sites chosen for the vectors of the *Bacillus subtilis* BioBrick Box are commonly used for insertion of genetic constructs into the *B. subtilis* chromosome. The *sacA*, *lacA* and *amyE* genes encode metabolic enzymes required for the degradation of different alternative carbohydrates, i.e. sucrose, galactans and starch, respectively [42-44]. Accordingly, their expression is regulated by sugar availability [42,43,45], which is easy to control by experimental design. The fourth locus, *thrC*, is required for synthesis of threonine and amino acids derived from it. Expression of the *hom-thrC-thrB* operon was found to be high under many of the experimental conditions tested in a recent expression profiling study of *B. subtilis*[46]. While this is not surprising for an amino acid biosynthesis operon, it has major implications for use as an integration site. Regulation of the operon’s promoter, P_*hom*_, is not well understood in *B. subtilis*, and we therefore constructed a transcriptional P_*hom*_-*luxABCDE* fusion to study the promoter activity under different growth conditions.

During growth in LB medium, representing amino acid-replete conditions, expression of P_*hom*_-*luxABCDE* was at an intermediate level, reaching a maximum of 10^5^ RLU/OD (Additional file 3: Figure S1). However, in the defined CSE medium, promoter activities were strongly increased and reached twelve times higher values than in LB medium. Addition of 0.1% casamino acids reduced these activities to two-fold above LB, and in the presence of 1% casamino acids expression was similar to growth in LB (Additional file 3: Figure S1). These data show that some considerations are required when using *thrC* as an integration site. That is, for applications in minimal or defined media, any construct to be integrated into *thrC* should contain a strong terminator upstream in order to prevent undesired read-through from P_*hom*_.

### The promoters of the *Bacillus* BioBrick Box

Promoters are crucial control elements for developing complex *in vivo* expression programs. For this purpose, they do not only have to be standardized, but also have to show a predictable and reliable activity under a variety of different conditions. For the optimization of metabolic pathways, the fine-tuning of gene expression is indispensable. Those features can sometimes be provided by inducible promoters or by developing promoter libraries [47-49], as described for *E. coli*[50,51]. To provide a first set of characterized promoter-parts for *B. subtilis*, we constructed and evaluated four constitutive promoters of different strength, as well as two inducible promoters that allow inducer-controlled gene expression levels.

#### Constitutive promoters

For our BioBrick Box, we chose three native σ^A^-dependent *B. subtilis* promoters and eleven representatives of the Anderson promoter collection [51], which are often used as a gold standard for constitutive gene expression in *E. coli*. The native *B. subtilis* promoters comprise P_*liaG*_, expressing regulatory components of the cell envelope stress response system [28], P_*lepA*_, expressing highly conserved genes in bacteria and mitochondria necessary for exact protein translation [29], and P_*veg*_, which is the strongest constitutive promoter in *B. subtilis* known to date [27]. The latter controls transcription of the *veg* gene, which seems to have an important function during the outgrowth of spores [52].

**Figure 2:**
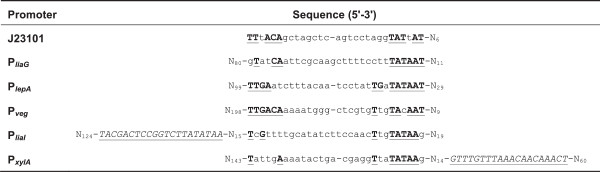
**Nucleotide sequences of the *Bacillus *BioBrick Box promoter collection.** Sequences are aligned according to their -35 and -10 region. All promoter fragments were designed with 3′-end exactly upstream of the RBS. The -10 and -35 regions (if conserved) are shown in bold and underlined. Regulator binding sites for XylR in P_*xylA*_ and LiaR in P_*liaI*_ are shown in italics and underlined. The native P_*xylA*_ also contains a catabolite responsive element (CRE) that suppresses the transcription in the presence of glucose [53]. This binding site is downstream of the promoter and is not present in the P_*xylA*_ –fragment used here, which is derived from the pXT vector [39].

All relevant sequence features of the promoters evaluated in this study are shown in Figure 2; the complete sequences of the cloned fragments can be found in Additional file 1. To determine their activity in *B. subtilis*, the promoter fragments were cloned into the luciferase reporter vector pBS3C*lux* and assayed in LB medium as well as in chemically defined MCSE medium (MOPS-based CSE, see Methods for details).

**Figure 3:**
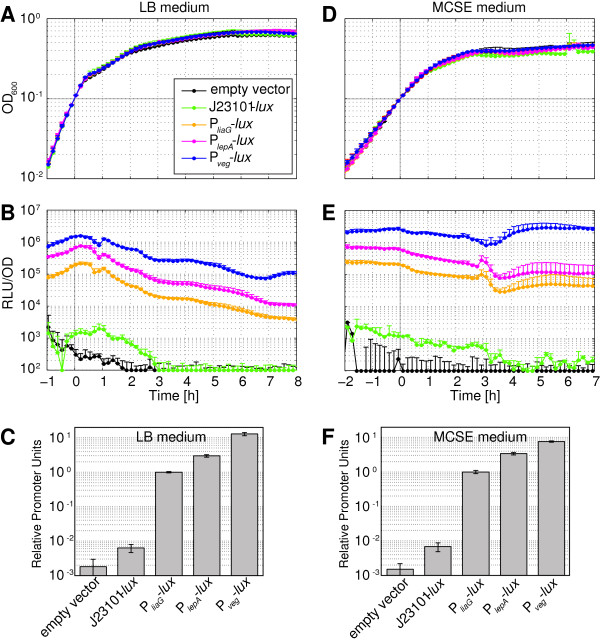
**Luciferase activities of the constitutive *Bacillus *BioBrick Box promoters.** Measurement of promoter activity was done based on pBS3C*lux*-derivatives via luminescence of 100 μl cultures growing in a plate reader (Biotek®, Synergy™2; 96-well plate, 37°C, shaking) over time. The graphs are aligned to an OD_600_ = 0.1 at time point 0 (*grey line*). **(A)**-**(C)** show data of growth in complex LB medium; **(D)**-**(F)** show data of growth in the defined medium MCSE. **(A)** and **(D)** Growth curves (OD_600_) from cultures of the reporter strains TMB1872 (*lux* without promoter), TMB1862 (J23101-*lux*), TMB1856 (P_*liaG*_-*lux*), TMB1860 (P_*lepA*_-*lux*) and TMB1930 (P_*veg*_*-lux*). **(B)** and **(E)** Promoter activities of the same strains expressed as luminescence in relative luminescence units (RLU) per OD_600_ and using the same color code as in panels **(A)** and **(D)**. **(C)** and **(F)** Bar graphs of relative promoter units (RPUs, normalized to P_*liaG*_) at time point 0, corresponding to OD_600_ = 0.1. All graphs show mean values and standard deviations (error bars) of at least four biological replicates.

Figure 3 shows both cell growth (Figure 3A/D) and the resulting luciferase activities (Figure 3B/E) over an extended growth period, ranging from exponential into the stationary growth phase in the two growth media. The activities, expressed as relative luminescence units (RLU) per OD_600_, covered about three orders of magnitude (Figure 3B/E), ranging from ~10^3^ RLU/OD for the Anderson promoter J23101 to ~10^6^ RLU/OD for the strong P_*veg*_ promoter. For growth in LB, promoter activities varied within a factor of two to three during exponential phase, and declined during stationary phase by more than one order of magnitude (Figure 3B). These variations likely arise from metabolic changes that the cells undergo during growth in this complex medium, as also reflected in the ever-changing growth rate in LB medium (Figure 3A). In contrast, the growth rate in the chemically defined MCSE medium was more constant in exponential phase (Figure 3D) and luciferase activities displayed less dynamic variation than in LB medium (Figure 3E).

While the Anderson promoter J23101 is a strong promoter in *E. coli*[51], deviating from the *E. coli* consensus promoter sequence by only a single nucleotide exchange in the -35 promoter region (TTGACA to TTTACA), it was the only of eleven different Anderson promoters tested in *B. subtilis* that generated a measurable luminescence output at all (data not shown). Still, compared to the values measured in *E. coli*, its activity was surprisingly low and only about ten-fold above the background of the empty vector (Figure 3B/E). This result clearly demonstrates the context-dependence of expression signals and hence stresses the importance of establishing organism-specific parts.

The absolute luciferase activities as documented in Figure 3B/E do not only depend on the promoter strength, but are also dependent on experimental conditions and setups, which can vary significantly between labs and experiments. However, while absolute values might vary, the relative strength of the promoters has proven to be a rather robust and comparable unit between several experiments and even between different research groups [54]. Towards that end, a suitable standard promoter, measured under the same experimental conditions as the promoter under consideration, serves as reference value, and promoter activities are expressed relative to this standard promoter as *relative promoter units* (RPUs) [54]. The stable expression behavior and intermediate strength of P_*liaG*_ makes it ideally suited as a reference promoter, and hence we propose to use P_*liaG*_ for calculating RPUs in *B. subtilis*. The RPUs for all constitutive promoters, calculated by dividing their activity at an OD_600_ of 0.1 by the activity of P_*liaG*_ are shown in Figure 3C/F.

#### The bacitracin-inducible promoter P_liaI_

Next, we chose the bacitracin-inducible promoter P_*liaI*_ for our BioBrick Box, due to its high dynamic range and low basal activity [30,31]. P_*liaI*_ controls the *liaIH* operon of *B. subtilis*, which is the main target of the envelope stress-inducible two-component system LiaRS. Upon induction with the cell wall antibiotic bacitracin, LiaS activates the cognate response regulator LiaR, which in turn strongly induces P_*liaI*_[28,55].

**Figure 4:**
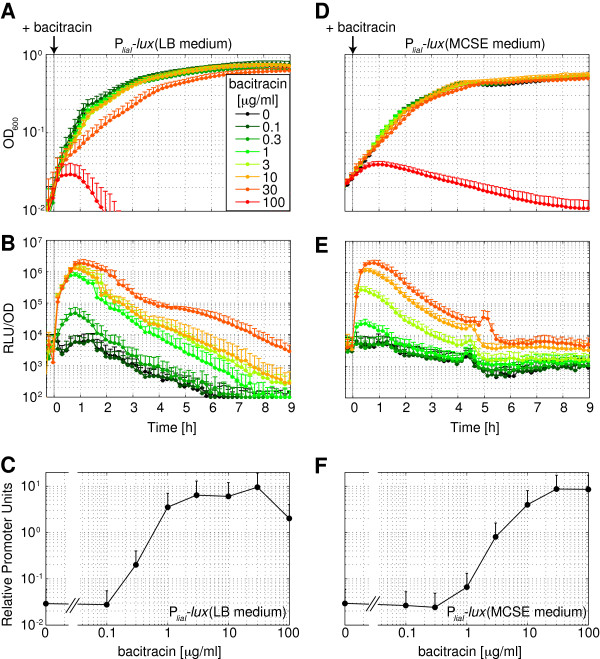
**Luciferase activity of the bacitracin-inducible P**_***liaI ***_**promoter.** Measurement of P_*liaI*_ activity in pBS3C*lux* upon induction with various bacitracin concentrations indicated in the legend. Addition of water was used as non-induced reference. For details of growth and measurement conditions, see legend of Figure 3 and Methods. **(A)**-**(C)** Growth in LB medium. **(D)**-**(F)** Growth in MCSE medium. **(A)** and **(D)** Growth curves (OD_600_) from cultures of the reporter strain TMB1858 (P_*liaI*_-*lux*). **(B)** and **(E)** Promoter activities upon bacitracin induction expressed as luminescence in RLU/OD_600._**(C)** and **(F)** Dose–response curves one hour post-induction in RPUs (normalized to P_*liaG*_). The data on induction with 100 μg/ml bacitracin was omitted in **(B)** and **(E)** due to lysis of the culture. All graphs show mean values and standard deviations (error bars) of four biological replicates.

For quantitative analysis, P_*liaI*_ was cloned into the luciferase reporter vector pBS3C*lux*, grown to exponential phase and induced with different concentrations of bacitracin (see Methods for details). Both in LB as well as in MCSE medium we observed a transient, concentration-dependent increase of luciferase activities for about two to four hours post-induction, which decreased about 100-fold towards stationary phase (Figure 4B/E). The maximal activity of about 300-fold induction was reached 1 hour after bacitracin treatment in LB (30 minutes in MCSE) at a concentration of 30 μg ml^-1^ bacitracin (Figure 4C/F), as shown previously [31]. Furthermore, induction with 30 μg ml^-1^ bacitracin leads to minor growth defects, while higher bacitracin concentrations resulted in the lysis of the culture (Figure 4A/D). The respective dose-response curves expressed in RPUs (normalized to P_*liaG*_) indicate that P_*liaI*_ responded more sensitively to low bacitracin concentrations in LB compared to MCSE medium (Figure 4C/F). This might reflect higher stress levels experienced due to faster growth in LB compared to MCSE medium (Figure 4A/D). Notably, the basal activity of P_*liaI*_ was approximately 0.03 RPU and hence – in the absence of a suitable inducer – perfectly fits into the “activity gap” between the constitutive promoters J23101 (0.006 RPU) and P_*liaG*_ (1 RPU).

#### The xylose-inducible promoter P_xylA_

Natively, P_*xylA*_ drives the expression of the *xylAB* operon in *B. subtilis*, which is required for using xylose as carbon and energy source. This promoter is regulated by XylR and CcpA and hence only induced by xylose if glucose is simultaneously absent, due to carbon catabolite repression [53]. The P_*xylA*_ derivative used in this study is amplified from the expression vector pXT [39] and lacks the catabolite-responsive element (CRE). As a result, the xylose-inducible promoter of the BioBrick Box is not subject to repression. Nevertheless, the choice of carbon source can still affect the xylose-dependent induction of P_*xylA*_ as described in more detail below.

**Figure 5:**
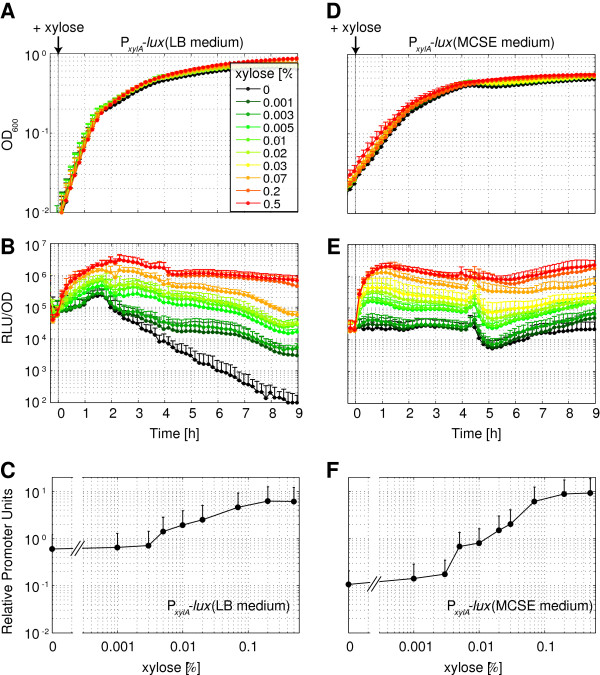
**Luciferase activity of the xylose-inducible P**_***xylA ***_**promoter.** Measurement of P_xyl_-activity in pBS3C*lux* upon induction with various concentrations (see legend) of xylose. Addition of water was used as non-induced reference. For details of growth and measurement conditions, see legend of Figure 3 and methods. **(A)**-**(C)** Growth in LB **(D)**-**(F)** Growth in MCSE. **(A)** and **(D)** Growth curves (OD_600_) from cultures of the reporter strain TMB1931 (P_*xylA*_-*lux*) in semi-logarithmic scale. **(B)** and **(E)** Promoter activities upon xylose-induction expressed as luminescence in RLU/OD_600_. **(C)** and **(F)** Dose–response curves one hour post-induction in RPUs (normalized to P_*liaG*_). All graphs show mean values and standard deviations (error bars) of at least four biological replicates.

The P_*xylA*_ reporter responded to xylose in a concentration-dependent manner in both LB and MCSE medium and reached its maximum activity about 1-1.5 hours post-induction (Figure 5B/E). Due to a significantly increased basal promoter activity in LB, there was only a ten-fold difference between minimal and maximal activity (Figure 5C), compared to a 100-fold difference in MCSE (Figure 5F). While the maximal activity of P_*xylA*_ was similar to P_*liaI*_, the basal activity of the non-induced promoter, especially in LB medium, was significantly higher for P_*xylA*_, resulting in a lower dynamic range. On the other hand, the activity of P_*xylA*_ was sustained, instead of transient for P_*liaI*_, and the sensitivities towards its inducer xylose were comparable in LB and MCSE medium (Figure 5C/F).

As mentioned above, removal of the CRE sequence from P_*xylA*_ removed the repressive effects of glucose via CcpA, but we still observed significantly reduced promoter activities during growth on glucose as carbon source. It was previously shown that glucose-6-phosphate competes with xylose for binding to the xylose repressor protein XylR and prevents its de-activation [56]. We therefore tested xylose-dependent induction of P_*xylA*_ during growth in CSE medium containing different carbon sources. During growth on glucose, addition of 0.2% xylose led to a 20-fold promoter induction, while up to 70-fold changes were observed during growth on fructose, mannitol or arabinose (Additional file 3: Figure S4). Metabolism of the latter three sugars does not produce glucose-6-phosphate as an intermediate and thus bypasses its repressive effect. The reporter strain showed similar growth rates during growth on glucose, fructose or mannitol (doubling time ca. 50 min), while growth on arabinose was markedly slower at ca. 65 min doubling times (Additional file 3: Figure S4 and data not shown). Based on these results we suggest using fructose as the carbon source for routine experiments in minimal or defined media when working with P_*xylA*_.

### The reporters of the *Bacillus* BioBrick Box

Reporter genes are important genetic tools for the measurement of promoter activities. The BioBrick Box therefore provides the established *lacZ*[57] and the *luxABCDE* reporter in the aforementioned reporter vectors pBS1C*lacZ* and pBS3C*lux*. To evaluate both vectors, we used the bacitracin-inducible *liaI* promoter to compare the characteristics of both reporters. The Miller units obtained for the *lacZ* reporter were compared to the RLU/OD_600_ values of the *luxABCDE* reporter one hour post-induction at identical inducer concentrations.

Both reporters showed a high dynamic range over about four orders of magnitude between the empty vector as background and the maximal P_*liaI*_ activity (Additional file 3: Figure S2). Importantly, we observed a linear correlation between P_*liaI*_*-lux* and P_*liaI*_*-lacZ* activity, demonstrating that each of the reporters provides an accurate measurement of promoter activity. Nevertheless, the *lux*-reporter has several advantages over *lacZ*: luminescence can be monitored online in growing cultures and neither requires the addition of a substrate nor performing a separate assay. Moreover, we have determined an average half-life of only 4.2 ± 0.3 min for the luciferase signal (Additional file 3: Figure S3), compared to a lifetime in the order of hours for β-galactosidase (data not shown). Hence, the *lux* reporter is particularly very well suited to resolve transcriptional responses occurring at timescales shorter than the cell-cycle. In fact, given this short lifetime one might expect that the luminescence activity follows the promoter activity almost instantly.

**Figure 6:**
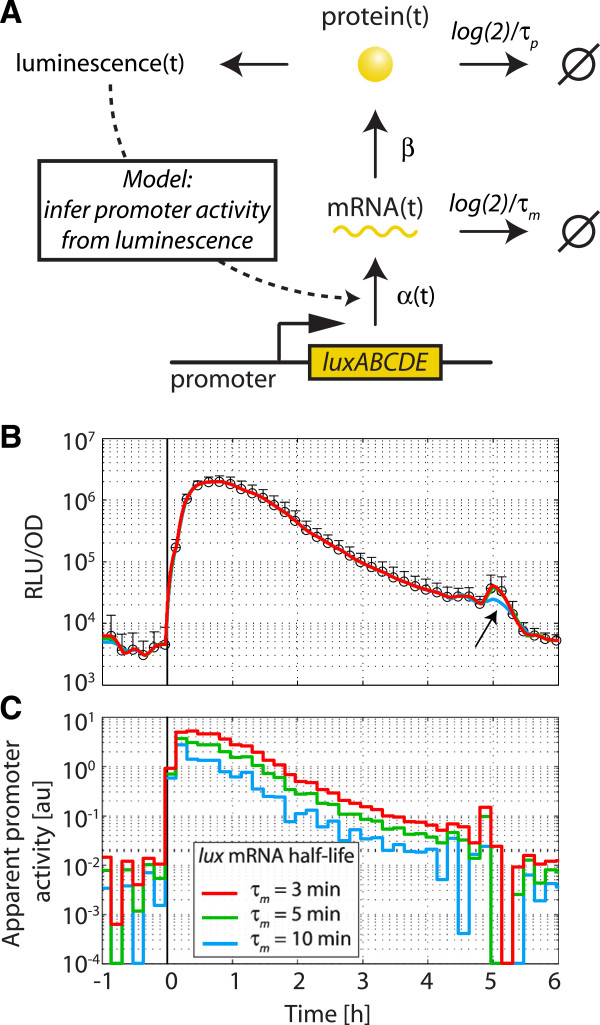
**Inference of promoter activity from luminescence measurements. (A)** Quantitative mathematical model used to infer the underlying promoter activity from luminescence time-series. Here, the promoter activity is reflected in a time-dependent transcription rate, α*(t). luxABCDE* mRNA is degraded at rate log(2)/τ_m_ , translated at rate β into Lux proteins, which are in turn degraded at rate log(2)/τ_p_. For simplicity, the model assumes a single Lux protein species, which is rate-limiting for luciferase activity and considered to be directly proportional to luminescence output. **(B)** As a test case for our method, the luciferase activity of pBS3C*lux*-P_*liaI*_ (TMB1858) induced with 30 μg/ml bacitracin (*symbols*) was fitted by our quantitative mathematical model (*solid lines*). Since the *luxABCDE* mRNA half-life is unknown in *B. subtilis,* we fitted the data using a physiological spectrum of half-lives (τ_m_ = 3, 5 and 10 min) and found that the model can reproduce the highly dynamic luciferase activity (*arrow*) only for mRNA half-lives of 5 min and shorter. **(C)** Underlying apparent promoter activity in arbitrary units (au) for the fits in **(B)**. For all details see main text.

In order to test how closely the luminescence may follow the underlying promoter activity, we devised a simplified mathematical model that includes the processes of *lux* mRNA transcription and degradation as well as Lux protein translation and decay (Figure 6A); see Methods for all assumptions and details. This model then enabled us to relate the time-dependent luminescence signal back to the time-dependent changes in the apparent promoter activity, by making the simplifying assumption that all changes in the luminescence signal are caused by changes in the transcription rate alone. As a proof-of-principle, we fitted the experimental luminescence activity of the P_*liaI*_ promoter induced with 30 μg/ml bacitracin in MCSE (Figure 6B; *symbols*) by our model (Figure 6B; *colored lines*), and extracted the underlying time-dependent apparent promoter activity (Figure 6C). Here it is clearly visible that the fit of the experimental data is best for an mRNA half-life of 5 min or shorter, since otherwise rapid changes in the luminescence signal cannot be reproduced within this model (Figure 6B; *arrow*). Strikingly, the model shows that the time course of the apparent promoter activity is almost identical to the luminescence activity, with only a ~10 min delay between promoter activity and the luminescence signal (Figure 6C), further underlining the power of the *lux* reporter in resolving highly dynamic transcriptional responses. It should be noted that the large fluctuations in apparent promoter activities at values below 10^-2^ arbitrary units are likely due to experimental noise and do not necessarily reflect true changes in transcription rate.

### The epitope tags of the *Bacillus* BioBrick Box

**Figure 7:**
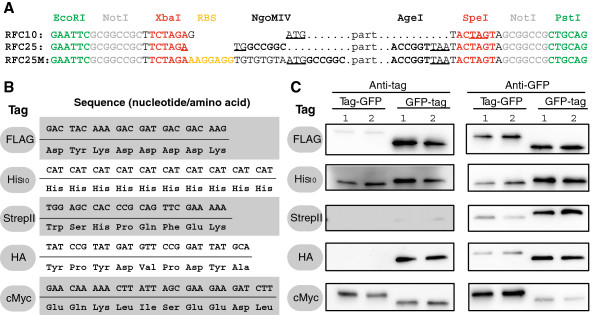
**Epitope tags of the *Bacillus *BioBrick Box. (A)** Pre- and suffixes of different cloning standards. The start- and the first stop-codon are underlined. RFC10 [3] describes the commonly used BioBrick standard. To combine two parts, the upstream part is cut with SpeI, the downstream one with XbaI (*red sites*). The compatible overhangs of the two isocaudomers can be ligated, and result in an eight basepair scar that cannot be recut but is optimal for RBS spacing. This causes a frameshift that is not suitable for translational fusions. RFC25 [11] describes the “Freiburg standard” which was developed by the 2007 iGEM-Team from Freiburg to allow translational fusions. Two parts can be combined similar to RFC10, but using NgoMIV and AgeI as restriction enzymes. The resulting scar ACCGGC codes for Threonine and Glycine. RFC25M refers to our modification of RFC25 by adding an optimized RBS (*yellow*). While this convenient modification simplifies the cloning procedure, it should be noted that as a consequence it also prevents downward compatibility, since the resulting BioBrick directly adds two functional DNA parts in a single step. The tag-BioBricks and *gfpmut1* are designed in that standard. **(B)** Nucleotide and amino acid sequences of the epitope tags, without the RFC25M pre- and suffixes shown in **(A)**. **(C)** Western blots of N- and C-terminal fusions of each tag to GFP, using the strains TMB1920 (Flag-*gfp*), TMB1921 (*gfp*-Flag), TMB1922 (HA-*gfp*), TMB1923 (*gfp*-HA), TMB1924 (cMyc-*gfp*), TMB1925 (*gfp*-cMyc), TMB1926 (His-*gfp*), TMB1927 (*gfp*-His), TMB1928 (StrepII-*gfp*) and TMB1929 (*gfp*-StrepII). For each construct, two independent clones were tested with epitope tag- and GFP-specific antibodies as a positive control.

Epitope tags are useful tools for both protein detection by Western blot analysis using tag-specific antibodies and for purification purposes or protein interaction studies. In our *Bacillus* BioBrick Box, we provide five widely used tags – FLAG-, His_10_-, StrepII-, HA-, and cMyc-tag – in a modified Freiburg standard that was developed to allow the generation of both N- and C-terminal translational fusions (Figure 7A). The DNA and protein sequences of all tags are shown in Figure 7B. To evaluate our tags, fusions to *gfpmut1* under control of a constitutive promoter were generated as described in Methods. Western blot analyses with GFP-specific antibodies showed that all proteins could be detected in the soluble protein fraction and were therefore correctly produced (Figure 7C). Analyses with tag-specific antibodies showed that addition of the different tags led to a slight shifting of apparent sizes between the N- and C-terminal fusions in the range of 25 to 30 kDa on the SDS-PAGE (Figure 7C).

The original FLAG-tag was developed as hydrophilic hexapeptide marker sequence (Lys-Asp-Asp-Asp-Asp-Lys) that is located at the outside of protein surfaces. Adaptation to *B. subtilis* was performed by Kaltwasser *et al.*[34]. In Western blots, the C-terminal fusion to GFP can be detected very well, while the signal for the N-terminal fusion was weak. The His_10_-tag was developed and is used for affinity purification on Ni^2+^-NTA-columns and Western blotting [33]. Both the N- and C-terminal fusions to GFP were easily detectable in Western blots (Figure 7C). The Strep-tag II, which was optimized from the original Strep-tag to allow both N- and C-terminal fusion, can be used together with the streptavidin-derivative Strep-Tactin as its specific binding partner [35,36]. The expression of the N-terminal fusion seemed to be rather low (anti-GFP blot) and detection with anti-strep antibodies was weak. Nevertheless, Strep-tagII is the epitope of choice for co-immunoprecipitation in protein-protein interaction studies in *B. subtilis* due to the highly specific interaction with Strep-Tactin-covered magnetic beads or resins [58]. The HA-tag was named after the human influenza *h*em*a*gglutinin, a viral surface protein that is important for host cell infection [37]. The corresponding HA-tag was codon-optimized for *B. subtilis*[34] and worked very well for detection of C-terminally tagged GFP. The cMyc-tag is derived from the human *c-myc* proto-oncogene [38], which was also optimized for *B. subtilis*[34]. C-terminal fusion to *gfp* seemed to negatively influence the GFP-expression levels. However, the detection with anti-cMyc-antibodies worked well for both N- and C-terminal fusions.

In summary, all five epitope tags were shown to be functional. The ideal terminus for creating protein-tag fusions as well as the most suitable tag will have to be determined experimentally for each target protein and application. For such studies, the parts from the BioBrick Box offer a convenient and fast way for optimization of conditions and constructs.

## Conclusion

In this study, we described the construction and evaluation of a toolkit containing essential vectors, promoters and epitope-tags for standardized genetic assembly in *B. subtilis*, a biotechnological workhorse and Gram-positive model organism.

We modified and adapted five integrative *B. subtilis* vectors to the BioBrick standard. Three are empty vectors that are fully compatible to each other and allow their combination in one single strain due to different integration loci and resistance cassettes. Two additional reporter vectors harbor *lacZ* and *luxABCDE* cassettes for evaluating transcriptional promoter fusions. While β-galactosidase is already long established as a reporter and needs no further introduction, we conducted a detailed study of the *lux*-reporter system. We determined the half-life of the luciferase signal as 4.2 ± 0.3 min, making the reporter instable enough to directly monitor both induction and repression of promoters almost in real time, as revealed by our mathematical model for the *lux*-reporter system. This feature, together with the very low background activity and the possibility to determine promoter activities directly in growing cultures, makes this an ideal reporter system, especially since the *lux* cassette from *P. luminescence* present in pBS3C*lux* does not require the addition of any luciferase substrate.

Four constitutive promoters and the uninduced P_*liaI*_ offer an activity range of three orders of magnitude with stable expression levels in defined MCSE medium and during exponential growth phase in LB medium. We adopted the concept of RPUs for *B. subtilis* and propose the P_*liaG*_ promoter to be used as a reference standard for RPU determination in this organism. The *Bacillus* BioBick Box also contains two inducible promoters, P_*liaI*_ and P_*xylA*_, which were thoroughly evaluated with the inducers bacitracin and xylose, respectively. Using inducer concentrations over about three orders of magnitude, the two promoters revealed a dynamic range of 300- or 100-fold activity over background, respectively. P_*liaI*_ has a transient activity in complex and defined medium, whereas P_*xylA*_ displays sustained induction.

Finally, we evaluated five widely used epitope tags as N- and C-terminal fusions to GFP via Western blotting. All are functional, but results for the test case GFP indicate a context-dependence in immuno-detection: The FLAG- and HA-tag were only suitable for C-terminal fusions to GFP, whereas His_10_ and cMyc worked both for N- and C-terminal fusions.

Taken together, we provide a set of well-evaluated standardized tools for the work in *B. subtilis*. We hope that this toolkit will inspire not only future iGEM-teams to choose *B. subtilis* as their chassis organism, but also greatly facilitate the engineering of this versatile model organism for synthetic biology applications in the future.

## Methods

### Bacterial strains and growth conditions

*B. subtilis* and *E. coli* were routinely grown in Luria-Bertani (LB) medium (1% (w/v) tryptone, 0.5% (w/v) yeast extract, 1% (w/v) NaCl) at 37°C with agitation (200 rpm). All strains used in this study are listed in Additional file 3: Table S2. Selective media for *B. subtilis* contained kanamycin (10 μg ml^-1^), spectinomycin (100 μg ml^-1^), chloramphenicol (5 μg ml^-1^), or erythromycin in combination with lincomycin (1 μg ml^-1^; 25 μg ml^-1^ for mls^r^). Selective media for *E. coli* contained ampicillin (100 μg ml^-1^) or chloramphenicol (35 μg ml^-1^). Solid media additionally contained 1.5% (w/v) agar.

### Transformation

*E. coli* (XL1 blue, Stratagene) competent cells were prepared and transformed according to OpenWetWare [59]. Transformations of *B. subtilis* were carried out as described previously [21]. The integration of plasmids into the *B. subtilis* genome was checked on starch plates (*amyE*), with minimal medium (*thrC*) or colony PCR (*sacA*, *lacA*). Detailed protocols can be found in the Additional file 3 and [60].

### *M*OPS-based *c*hemically defined medium with *s*uccinate and glutamate (MCSE)

The chemically defined CSE medium (3.3 g l^-1^ (NH_4_)_2_SO_4_, 29 mM KH_2_PO_4_, 70 mM K_2_HPO_4_, 1 × III’-salts (100 × III’-salts: 0.232 g l^-1^ MnSO_4_ × 4 H_2_O, 12.3 g l^-1^ MgSO_4_ × 7 H_2_O), 50 mg l^-1^ Tryptophan, 22 mg l^-1^ammonium ferric citrate, 0.8% (w/v) K-glutamate, 0.6% (w/v) Na-succinate, up to 2.5% (w/v) C-source) is used for experiments with stable growth conditions and a defined C-source [61]. In long-term experiments (18 hours), we noticed a white precipitate disturbing the OD_600_-measurements in the microplate reader. We tested the use of a phosphate-reduced 3-(N-Morpholino)-Propanesulfonic acid (MOPS) buffer (10 × MOPS buffer: 83.72 g l^-1^ MOPS, 33 g l^-1^ (NH_4_)_2_SO_4_, 3.85 mM KH_2_PO_4_, 6.15 mM K_2_HPO_4_; adjusted to pH 7 with KOH), which did not cause precipitation. This is the composition of the newly developed MOPS-based MCSE medium: 1 × MOPS, 50 mg l^-1^ Tryptophan, 22 mg l^-1^ammonium ferric citrate, 1 × III’-salts, 0.8% (w/v) K-glutamate, 0.6% (w/v) Na-succinate, 0.2% (w/v) fructose. The C-source for MCSE can be varied according to the experimental requirements.

### DNA manipulation and plasmid construction

General cloning procedure, such as endonuclease restriction digest, ligation and PCR, was performed with enzymes and buffers from New England Biolabs (NEB; Ipswich, MA, USA) according to the respective protocols. For subsequent cloning, Phusion® polymerase was used for PCR amplifications, otherwise OneTaq® was the polymerase of choice. PCR-purification was performed with the *HiYield PCR Gel Extraction/PCR Clean-up Kit* (Süd-Laborbedarf GmbH (SLG), Gauting, Germany). Plasmid preparation for mutagenesis-PCR templates was performed with the *HiYield Plasmid Mini-Kit* (SLG) according to the manufacturer’s protocol, otherwise by alkaline lysis plasmid preparation. All plasmids created during this study are listed in Additional file 3: Table S1, all primer sequences are given in Additional file 3: Table S3.

### Vectors of the *Bacillus* BioBrick Box

In all vectors, the PstI site in the *bla* gene was removed by site-directed mutagenesis using the oligonucleotides TM2206 and TM2207. The resulting vector is marked with the auxiliary “*bla*mut”. Primer design and mutagenesis were performed according to the manufacturer’s instructions for the QuikChange® II Site-Directed Mutagenesis Kit (Agilent Technologies). All plasmids were test-digested to confirm removal of the restriction site.

To create the empty vector **pBS1C**, pDG1662*bla*mut was cut with PstI to remove one XbaI site and the spc^r^ outside of the integrative part. The 6 kb fragment was religated. The remaining PstI site was mutated via site-directed mutagenesis with the primers TM2845 and TM2846. To insert the MCS, the vector was cut with EcoRI. The MCS was amplified by PCR from pSB1C3 with the Primers TM2843 and TM2844, cut with EcoRI and BsaI (EcoRI-compatible overhang) and ligated into the vector. The remaining NgoMIV sites were removed by subsequent site-directed mutagenesis with TM3005 + TM3006, TM3011 + TM3012 and TM3013 + TM3014, respectively, resulting in pBS1C.

To create the empty vector **pBS2E**, pAX01 was cut with SacI. The 6.3 kb fragment was religated to remove the *xylR*-P_*xylA*_-fragment. The PstI site in *bla* was removed. The vector was cut with XbaI and the 6 kb fragment religated to reduce the amount of forbidden restriction sites. The lost terminator of *erm* was replaced by the PCR-amplified terminator with BsaI-overhangs (primers: TM2975 and TM2976). The vector was opened with XbaI and ligated with the PCR product cut with BsaI (XbaI-compatible overhang). The correct direction was checked by sequencing and removal of the XbaI-site was confirmed. Finally, the MCS was amplified from pSB1C3 (TM2608 and TM2609) with PstI and NsiI overhangs, cut with PstI and NsiI and ligated into the PstI-cut vector. Correct orientation of the insert was confirmed by restriction digest and sequencing. The remaining NgoMIV sites were removed by subsequent site-directed mutagenesis with TM3011 + TM3012 and TM3028 + TM3029, respectively, resulting in pBS2E.

To create the empty vector **pBS4S**, the *erm*-resistance outside of the integrative part of pDG1731 was removed via cut-ligation with MluI and BssHI, resulting in a 4.7 kb vector. The PstI site in *bla* was removed as described above. The PstI site in *thrB* was removed performing site-directed mutagenesis with the primers TM2835 and TM2836. The *spc*-promoter with upstream PstI-overhang was amplified with the primers TM2837 and TM2838 and cut with PstI and PciI. The MCS was cut from pSB1C3 with EcoRI and PstI. The vector was cut with EcoRI and PciI and the 4.3 kb-fragment ligated with both cut DNA-fragments. The remaining NgoMIV sites were removed by subsequent site-directed mutagenesis with TM3005 + TM3006 and TM3011 + TM3012, respectively, resulting in pBS4S.

To create the reporter vector **pBS1C*lacZ***, pAC6*bla*mut was cut with EcoRI and PstI. The primers TM2301 and TM2302 (1 pM) were mixed, heated (95°C, 10 min), re-annealed (50°C, 10 min) to become double stranded with 4 bp overhangs and ligated into the vector. The vector was cut with EcoRI and PstI and ligated with the MCS from pSB1C3 cut with EcoRI and PstI. A PstI site in the *E. coli* ori was removed by cutting with BglII and religation of the 11 kb fragment.

To create the reporter vector **pBS3C*lux***, the PstI site in the *sacA* locus of pAH328*bla*mut was removed by site-directed mutagenesis with the primers TM2885 and TM2886. Then, the XbaI site of *luxD* was removed similarly with the Primers TM2887 and TM2888. The MCS was amplified by PCR from pSB1C3 with the primers TM2843 and TM2884, cut with EcoRI and BsaI (SpeI-overhang) and ligated into the EcoRI/SpeI-cut vector.

### Promoters of the *Bacillus* BioBrick Box

The promoter J23101 was used from the parts registry [2]. The promoters P_*liaG*_, P_*lepA*_, P_*veg*_ and P_*liaI*_ were amplified from *B. subtilis* W168 genomic DNA using the primer pairs TM2891/TM2892, TM2899/TM2900, TM2903/TM2904 and TM2895/TM2896, respectively. P_*xylA*_ was amplified from pXT [39] with the primers TM2968 + TM2969. The PCR products were cut with EcoRI and SpeI, ligated into pSB1C3, pBS3C*lux* and pBS1C*lacZ* (P_*liaI*_ only) and verified by sequencing.

### Epitope-tags of the *Bacillus* BioBrick Box

All eptitope tags were synthesized with pre- and suffixes (Figure 7A) together as a single part in a plasmid from GeneArt (Life Technologies Corporation, USA). The plasmid was cut with EcoRI and PstI and, without purification steps, ligated into pSB1C3 cut with EcoRI and PstI. The products were checked by sequencing for their correct insert. *gfpmut1* was adapted to modified Freiburg standard by PCR with the primers TM2934/TM2935 and pGFPamy as template and cloned into pSB1C3 (both cut with EcoRI and SpeI). For translational fusion, the pSB1C3-tag-constructs were cut open with AgeI + PstI (tag is N-terminal) or EcoRI + NgoMIV (tag is C-terminal) and purified. *gfp* was cut with the corresponding restriction enzymes of the Freiburg standard, purified from an agarose gel and ligated into the vector. The tag-*gfp* or *gfp*-tag fusions as well as pBS0KPspac* were cut with EcoRI and PstI, purified from agarose gels, ligated and verified by sequencing.

### Luciferase assay

Luciferase activities of strains harbouring pBS3C*lux*-derivates were assayed using a Synergy™2 multi-mode microplate reader from BioTek**®** (Winooski, VT, USA). The reader was controlled using the software Gen5™. Culture volumes were 100 μl per well in 96-well plates (black walls, clear bottom; Greiner Bio-One, Frickenhausen, Germany), and incubation occurred at 37°C with agitation (intensity: medium). Cell growth was monitored by optical density at 600 nm wavelength (OD_600_). Raw luminescence output (relative luminescence units, RLU) was normalized to cell density by dividing each data-point by its corresponding corrected OD_600_ value (RLU/OD).

For constitutive promoters, LB or MCSE medium was inoculated 1:500 from overnight cultures of each strain. Cultures were incubated at 37°C with agitation and OD_600_ as well as luminescence were monitored every 10 min for at least 13 hours. For inducible promoters, 10 ml of LB or MCSE medium were inoculated 1:500 from overnight cultures and grown to OD_600_ = 0.2-0.5. Those pre-cultures were diluted to OD_600_ = 0.05 (MCSE) or 0.01 (LB), respectively, and transferred to eight (P_*liaI*_) or ten (P_*xylA*_) wells of a 96-well plate. The OD_600_ as well as luminescence were monitored every 10 min for one hour. At an OD_600_ ~ 0.1 (corresponding to OD_600_ ~ 0.4 in cuvettes of 1 cm light path length) 5 μl of the inducer was added to final concentrations of 0.1, 0.3, 1, 3, 10, 30 or 100 μg ml^-1^ Zn^2+^-bacitracin or 0.001, 0.003, 0.005, 0.01, 0.02, 0.03, 0.07, 0.2, 0.5% (w/v) xylose. To one culture-well, 5 μl of water was added as uninduced control. Cultures were incubated at 37°C with agitation and the OD_600_ as well as luminescence were monitored every 10 min for at least 13 hours.

### β-Galactosidase assay

LB medium (30 ml) was inoculated 1:100 from fresh overnight culture of strains harboring pBS1C*lacZ*-P_*liaI*_ or pBS1C*lacZ* without promoter and grown at 37°C, shaking at 200 rpm. At OD_600_ = 0.4-0.6, the culture was split into 3 ml samples and induced with 30 μl of Zn^2+^-bacitracin to the final concentrations of 0.1, 0.3, 1, 3, 10, 30 or 100 μg ml^-1^. To one sample, 30 μl of water was added as a negative control. After incubation for another 30 min, 2 ml of the samples were harvested by centrifugation (13000 × *g*, 1-3 min) and the pellets were stored at -20°C. For the assay, the pellets were resuspended in 1 ml of working buffer and assayed for β-galactosidase activity as described elsewhere, with normalization to cell density [62].

### Western blot analysis

To verify the functionality of the epitope tags, Western blot analyses of the strains TMB1920-TMB1929 were performed. LB medium (15 ml) was inoculated 1:100 from overnight culture and grown at 37°C and 200 rpm to OD_600_ ~ 0.5. Of this, 10 ml were harvested by centrifugation (8000 × *g*, 5 min) and the pellets stored at -20°C. Pellets were resuspended in 1 ml disruption buffer (50 mM Tris–HCl pH 7.5, 100 mM NaCl) and lysed by sonication. Samples (12 μl of lysate) were loaded per lane on two 12.5% SDS-polyacrylamide gels and SDS-PAGE was performed according standard procedure [60]. One gel was stained with colloidal coomassie, the other one was used for protein transfer to a PVDF membrane (Merck Millipore, Billerica, MA, USA) by submerged blotting procedure (Mini Trans-Blot Electrophoretic Transfer Cell (Bio-Rad, Hercules, CA, USA)). After protein transfer, the membranes were treated with the following antibodies and conditions. Detailed protocols can be found in the Additional file 3.

#### GFP

Probing with primary antibodies takes place with rabbit anti-GFP antibodies (1:3000, Epitomics, No. 1533). Horseradish-peroxidase (HRP)-conjugated anti-rabbit antibodies (1:2000, Promega, W401B) were used as secondary antibody. Hybridization of both antibodies was carried out in Blotto-buffer (2.5% (w/v) skim milk powder, 1 × TBS (50 mM Tris–HCl pH 7.6, 0.15 M NaCl)).

#### FLAG

Rabbit anti-FLAG (1:2000, Sigma, Anti-Flag polyclonal, F7425) and anti-rabbit-HRP (1:2000, Promega, W401B) were used in Blotto-buffer.

#### His_10_

Mouse-anti-Penta-His (1:2000, Qiagen, Penta-His, No. 34660) in 1 × TBS, 5% bovine serum albumin (BSA, Fraction V; Carl ROTH) and anti-mouse-HRP (1:2000, Promega, W402B1) in 1 × TBS, 10 % (w/v) skim milk powder were used.

#### StrepII

*Strep*-Tactin-HRP conjugate (IBA, Strep-Tactin-HRP conjugate, No. 2-1502-001) 1:100 in 1 × PBS (4 mM KH_2_PO_4_; 16 mM Na_2_HPO_4_; 115 mM NaCl) with 0.1% (w/v) Tween20 was used.

#### HA

Rabbit anti-HA (1:500, Sigma, H6908) in TBS, 0.05% (w/v) Tween20, 5% (w/v) skim milk powder and anti-rabbit-HRP (1:2000, Promega, W401B) in Blotto-buffer were used.

#### cMyc

Rabbit anti-Myc **(**1:2000, Abcan, ab9106) in TBS, 0.05% (w/v) Tween20, 5% (w/v) skim milk powder and anti-rabbit-HRP (1:2000, Promega, W401B) in Blotto-buffer were used.

Chemiluminescence signals were detected after addition of the HRP-substrate Ace Glow (Peqlab, Erlangen, Germany) using a Fusion™ imaging system (Peqlab).

### Determination of luciferase half-life

To determine the half-life of the output of the luciferase reporter system, *B. subtilis* harboring the P_*xyl*_-*luxABCDE* reporter construct was grown in CSE medium in the presence of 0.15% (w/v) xylose under the conditions described for luciferase assays with constitutive promoters. When luciferase activities reached approximately 10^5^ RLU/OD_600_ (early exponential phase), further protein synthesis was stopped by the addition of 500 μg ml^-1^ tetracycline, and luminescence and OD_600_ were monitored every 5 min for 1 h. The half-life of the luminescence output was determined from a fit of the data from eight replicate assays with an exponential decay function (Additional file 3: Figure S3).

### Data analysis of luciferase assays

For each individual sample, the optical density (OD_600_) and luminescence values (RLU) were background-corrected by subtracting the respective values measured for wells containing 100 μl of the corresponding medium. Only for the measurements of the constitutive promoters, in which wells were inoculated with cells right from the start of the experiment, the OD_600_ of pure medium was initially higher than the OD_600_ of culture-filled wells, such that the average values of culture-filled wells at t = 0 h were used as blank for OD_600_. In order to compensate for sample-to-sample variations in the growth lag of individual cultures, we aligned all individual measurements of the constitutive promoters on the time-axis, such that OD_600_ = 0.1 was shifted to t = 0 h. Subsequently RLU/OD_600_ values were calculated for individual measurements and for each condition both mean and standard deviation of RLU/OD_600_ values were determined from at least three biological replicates.

### Mathematical model for promoter activity

To get an estimate for the time-dependence of the promoter activity that results in a given luciferase activity, we set up a simple mathematical model for the expression of the Lux-system (Figure 6). For simplicity, the model assumes that one of the Lux proteins is rate-limiting for light production, such that the luminescence is proportional to this protein species. The corresponding differential equations for the concentration of *luxABCDE* mRNA, *m(t)*, and the rate-limiting Lux protein, *p(t)*, read

(1) $$\frac{d}{\mathrm{dt}}m\left(t\right)=\alpha \left(t\right)-\frac{\log\left(2\right)}{\tau_{m}}m\left(t\right)\;$$

and

(2) $$\frac{d}{\mathrm{dt}}p\left(t\right)=\beta m\left(t\right)-\frac{\log\left(2\right)}{\tau_{p}}p\left(t\right),$$

where α*(t)* is the time-dependent apparent transcription rate, β the translation rate and τ_*m*_ and τ_*p*_ the mRNA and protein half-lives, respectively. Although mRNA and protein stability might also depend on cell physiology, time depended changes in these parameters were ignored to avoid further unknown parameters. Likewise, we did not model the kinetics of inducer uptake/signal transduction explicitly, but subsumed these processes in the kinetics of the time-dependent apparent transcription rate. For simplicity, the translation rate was directly expressed in units of RLU/mRNA/min, such that *p(t)* has units of RLU. The model in Eqs. (1) and (2) was solved analytically for *p(t),* and then fitted to the experimental luminescence trajectories. To this end, all parameters except α*(t)* were fixed to the following values: τ_*p*_ = 4.2 min (experimentally determined in Additional file 3: Figure S3), β = 10^4^ RLU/mRNA/min (arbitrary, but adjusted to meet typical transcription rates), τ_*m*_ = 3, 5 and 10 min (typical values for bacterial mRNA half-lives [63]). Then α*(t)* was discretized into *N* intervals of constant transcription rate α_*i*_*(i = 1,…N)* (the length of the intervals was adjusted to ∆t = 10 min as in the luciferase assay). Finally, the α_*i*_ were estimated by fitting *p(t)* to the luminescence trajectories using a trust-region reflective Newton method for least-squares minimization (MATLAB, The MathWorks, Inc.).

## Competing interests

The authors declare that they have no competing interests.

## Authors’ contributions

TM, JR, KK, JB, TC, FD, JE and SK conceptualized the contents of the *Bacillus* BioBrick Box and designed strategies for the modification of the vectors. JR constructed all vectors. JR and KK cloned and evaluated the promoters. KK cloned the tag-*gfp*-fusions and performed all Western blots. SG and CS performed the analyses of P_*hom*_ activities and analyzed the influence of C-sources on P_*xyl*_ activation. GF performed the quantitative data analysis and mathematical modeling. JR, GF, SG and TM wrote the manuscript. TM supervised the 2012 iGEM team LMU-Munich and coordinated the experimental work. All authors read and approved the final manuscript.

## Authors’ information

Jara Radeck, Korinna Kraft, Julia Bartels, Tamara Cikovic, Franziska Dürr, Jennifer Emenegger and Simon Kelterborn were members of the 2012 iGEM-team LMU-Munich.

## Supplementary Material

Additional file 1 **GeneBank files of *Bacillus *** **BioBricks.** Click here for file

Additional file 2 Alignment of original and final vectors. Click here for file

Additional file 3 Supplemental Figures, Tables and Text. Click here for file
