# Supplementary material for: The Bacillus BioBrick Box: generation and evaluation of essential genetic building blocks for standardized work with Bacillus subtilis
Source: J Biol Eng. 2013 Dec 2;7:29. doi: 10.1186/1754-1611-7-29 (PMC4177231; doi:10.1186/1754-1611-7-29)
Supplement: Additional file 2 — Alignment of original and final vectors. [file 1754-1611-7-29-S2.docx]

Supplementary Information to

**Radeck *et al*. “The *Bacillus* BioBrick Box: Generation and Evaluation of Essential Genetic Building Blocks for Standardized Work with *Bacillus subtilis***

Additional file 2 [.docx]: Alignment of original and final vectors.

Content

[1. Alignment of Sequence_1: pBS1C*lacZ* with Sequence_2: pAC6 2](#_Toc372549808)

[2. Alignment of Sequence_1: pAH328 with Sequence_2: pBS3C*lux* 16](#_Toc372549809)

[3. Alignment of Sequence_1: pBS2E with Sequence_2: pAX01 31](#_Toc372549810)

[4. Alignment of Sequence_1: pBS1C with Sequence_2: pDG1662 42](#_Toc372549811)

[5. Alignment of Sequence_1: pBS4S with Sequence_2: pDG1731 52](#_Toc372549812)

## Alignment of Sequence_1: pBS1C*lacZ* with Sequence_2: pAC6

Similarity : 9859/9918 (99,41 %)

Seq_1 1 gaattcgcggccgcttctagatggccggcaccggttaatactagtagcggccgctgcagg 60

||||||#||#################################################||

Seq_2 1 gaattcccg-------------------------------------------------gg 11

Seq_1 61 gatccacagtacataaaaaaggagacatgacgatggtcgttttacaacgtgactgggtcg 120

||||||||||||||||||||||||||||||||||||||||||||||||||||||||||||

Seq_2 12 gatccacagtacataaaaaaggagacatgacgatggtcgttttacaacgtgactgggtcg 71

Seq_1 121 accgggaaaaccctggcgttacccaacttaatcgccttgcagcacatccccctttcgcca 180

||||||||||||||||||||||||||||||||||||||||||||||||||||||||||||

Seq_2 72 accgggaaaaccctggcgttacccaacttaatcgccttgcagcacatccccctttcgcca 131

Seq_1 181 gctggcgtaatagcgaagaggcccgcaccgatcgcccttcccaacagttgcgcagcctga 240

||||||||||||||||||||||||||||||||||||||||||||||||||||||||||||

Seq_2 132 gctggcgtaatagcgaagaggcccgcaccgatcgcccttcccaacagttgcgcagcctga 191

Seq_1 241 atggcgaatggcgctttgcctggtttccggcaccagaagcggtgccggaaagctggctgg 300

||||||||||||||||||||||||||||||||||||||||||||||||||||||||||||

Seq_2 192 atggcgaatggcgctttgcctggtttccggcaccagaagcggtgccggaaagctggctgg 251

Seq_1 301 agtgcgatcttcctgaggccgatactgtcgtcgtcccctcaaactggcagatgcacggtt 360

||||||||||||||||||||||||||||||||||||||||||||||||||||||||||||

Seq_2 252 agtgcgatcttcctgaggccgatactgtcgtcgtcccctcaaactggcagatgcacggtt 311

Seq_1 361 acgatgcgcccatctacaccaacgtaacctatcccattacggtcaatccgccgtttgttc 420

||||||||||||||||||||||||||||||||||||||||||||||||||||||||||||

Seq_2 312 acgatgcgcccatctacaccaacgtaacctatcccattacggtcaatccgccgtttgttc 371

Seq_1 421 ccacggagaatccgacgggttgttactcgctcacatttaatgttgatgaaagctggctac 480

||||||||||||||||||||||||||||||||||||||||||||||||||||||||||||

Seq_2 372 ccacggagaatccgacgggttgttactcgctcacatttaatgttgatgaaagctggctac 431

Seq_1 481 aggaaggccagacgcgaattatttttgatggcgttaactcggcgtttcatctgtggtgca 540

||||||||||||||||||||||||||||||||||||||||||||||||||||||||||||

Seq_2 432 aggaaggccagacgcgaattatttttgatggcgttaactcggcgtttcatctgtggtgca 491

Seq_1 541 acgggcgctgggtcggttacggccaggacagtcgtttgccgtctgaatttgacctgagcg 600

||||||||||||||||||||||||||||||||||||||||||||||||||||||||||||

Seq_2 492 acgggcgctgggtcggttacggccaggacagtcgtttgccgtctgaatttgacctgagcg 551

Seq_1 601 catttttacgcgccggagaaaaccgcctcgcggtgatggtgctgcgttggagtgacggca 660

||||||||||||||||||||||||||||||||||||||||||||||||||||||||||||

Seq_2 552 catttttacgcgccggagaaaaccgcctcgcggtgatggtgctgcgttggagtgacggca 611

Seq_1 661 gttatctggaagatcaggatatgtggcggatgagcggcattttccgtgacgtctcgttgc 720

||||||||||||||||||||||||||||||||||||||||||||||||||||||||||||

Seq_2 612 gttatctggaagatcaggatatgtggcggatgagcggcattttccgtgacgtctcgttgc 671

Seq_1 721 tgcataaaccgactacacaaatcagcgatttccatgttgccactcgctttaatgatgatt 780

||||||||||||||||||||||||||||||||||||||||||||||||||||||||||||

Seq_2 672 tgcataaaccgactacacaaatcagcgatttccatgttgccactcgctttaatgatgatt 731

Seq_1 781 tcagccgcgctgtactggaggctgaagttcagatgtgcggcgagttgcgtgactacctac 840

||||||||||||||||||||||||||||||||||||||||||||||||||||||||||||

Seq_2 732 tcagccgcgctgtactggaggctgaagttcagatgtgcggcgagttgcgtgactacctac 791

Seq_1 841 gggtaacagtttctttatggcagggtgaaacgcaggtcgccagcggcaccgcgcctttcg 900

||||||||||||||||||||||||||||||||||||||||||||||||||||||||||||

Seq_2 792 gggtaacagtttctttatggcagggtgaaacgcaggtcgccagcggcaccgcgcctttcg 851

Seq_1 901 gcggtgaaattatcgatgagcgtggtggttatgccgatcgcgtcacactacgtctgaacg 960

||||||||||||||||||||||||||||||||||||||||||||||||||||||||||||

Seq_2 852 gcggtgaaattatcgatgagcgtggtggttatgccgatcgcgtcacactacgtctgaacg 911

Seq_1 961 tcgaaaacccgaaactgtggagcgccgaaatcccgaatctctatcgtgcggtggttgaac 1020

||||||||||||||||||||||||||||||||||||||||||||||||||||||||||||

Seq_2 912 tcgaaaacccgaaactgtggagcgccgaaatcccgaatctctatcgtgcggtggttgaac 971

Seq_1 1021 tgcacaccgccgacggcacgctgattgaagcagaagcctgcgatgtcggtttccgcgagg 1080

||||||||||||||||||||||||||||||||||||||||||||||||||||||||||||

Seq_2 972 tgcacaccgccgacggcacgctgattgaagcagaagcctgcgatgtcggtttccgcgagg 1031

Seq_1 1081 tgcggattgaaaatggtctgctgctgctgaacggcaagccgttgctgattcgaggcgtta 1140

||||||||||||||||||||||||||||||||||||||||||||||||||||||||||||

Seq_2 1032 tgcggattgaaaatggtctgctgctgctgaacggcaagccgttgctgattcgaggcgtta 1091

Seq_1 1141 accgtcacgagcatcatcctctgcatggtcaggtcatggatgagcagacgatggtgcagg 1200

||||||||||||||||||||||||||||||||||||||||||||||||||||||||||||

Seq_2 1092 accgtcacgagcatcatcctctgcatggtcaggtcatggatgagcagacgatggtgcagg 1151

Seq_1 1201 atatcctgctgatgaagcagaacaactttaacgccgtgcgctgttcgcattatccgaacc 1260

||||||||||||||||||||||||||||||||||||||||||||||||||||||||||||

Seq_2 1152 atatcctgctgatgaagcagaacaactttaacgccgtgcgctgttcgcattatccgaacc 1211

Seq_1 1261 atccgctgtggtacacgctgtgcgaccgctacggcctgtatgtggtggatgaagccaata 1320

||||||||||||||||||||||||||||||||||||||||||||||||||||||||||||

Seq_2 1212 atccgctgtggtacacgctgtgcgaccgctacggcctgtatgtggtggatgaagccaata 1271

Seq_1 1321 ttgaaacccacggcatggtgccaatgaatcgtctgaccgatgatccgcgctggctaccgg 1380

||||||||||||||||||||||||||||||||||||||||||||||||||||||||||||

Seq_2 1272 ttgaaacccacggcatggtgccaatgaatcgtctgaccgatgatccgcgctggctaccgg 1331

Seq_1 1381 cgatgagcgaacgcgtaacgcgaatggtgcagcgcgatcgtaatcacccgagtgtgatca 1440

||||||||||||||||||||||||||||||||||||||||||||||||||||||||||||

Seq_2 1332 cgatgagcgaacgcgtaacgcgaatggtgcagcgcgatcgtaatcacccgagtgtgatca 1391

Seq_1 1441 tctggtcgctggggaatgaatcaggccacggcgctaatcacgacgcgctgtatcgctgga 1500

||||||||||||||||||||||||||||||||||||||||||||||||||||||||||||

Seq_2 1392 tctggtcgctggggaatgaatcaggccacggcgctaatcacgacgcgctgtatcgctgga 1451

Seq_1 1501 tcaaatctgtcgatccttcccgcccggtgcagtatgaaggcggcggagccgacaccacgg 1560

||||||||||||||||||||||||||||||||||||||||||||||||||||||||||||

Seq_2 1452 tcaaatctgtcgatccttcccgcccggtgcagtatgaaggcggcggagccgacaccacgg 1511

Seq_1 1561 ccaccgatattatttgcccgatgtacgcgcgcgtggatgaagaccagcccttcccggctg 1620

||||||||||||||||||||||||||||||||||||||||||||||||||||||||||||

Seq_2 1512 ccaccgatattatttgcccgatgtacgcgcgcgtggatgaagaccagcccttcccggctg 1571

Seq_1 1621 tgccgaaatggtccatcaaaaaatggctttcgctacctggagagacgcgcccgctgatcc 1680

||||||||||||||||||||||||||||||||||||||||||||||||||||||||||||

Seq_2 1572 tgccgaaatggtccatcaaaaaatggctttcgctacctggagagacgcgcccgctgatcc 1631

Seq_1 1681 tttgcgaatacgcccacgcgatgggtaacagtcttggcggtttcgctaaatactggcagg 1740

||||||||||||||||||||||||||||||||||||||||||||||||||||||||||||

Seq_2 1632 tttgcgaatacgcccacgcgatgggtaacagtcttggcggtttcgctaaatactggcagg 1691

Seq_1 1741 cgtttcgtcagtatccccgtttacagggcggcttcgtctgggactgggtggatcagtcgc 1800

||||||||||||||||||||||||||||||||||||||||||||||||||||||||||||

Seq_2 1692 cgtttcgtcagtatccccgtttacagggcggcttcgtctgggactgggtggatcagtcgc 1751

Seq_1 1801 tgattaaatatgatgaaaacggcaacccgtggtcggcttacggcggtgattttggcgata 1860

||||||||||||||||||||||||||||||||||||||||||||||||||||||||||||

Seq_2 1752 tgattaaatatgatgaaaacggcaacccgtggtcggcttacggcggtgattttggcgata 1811

Seq_1 1861 cgccgaacgatcgccagttctgtatgaacggtctggtctttgccgaccgcacgccgcatc 1920

||||||||||||||||||||||||||||||||||||||||||||||||||||||||||||

Seq_2 1812 cgccgaacgatcgccagttctgtatgaacggtctggtctttgccgaccgcacgccgcatc 1871

Seq_1 1921 cagcgctgacggaagcaaaacaccagcagcagtttttccagttccgtttatccgggcaaa 1980

||||||||||||||||||||||||||||||||||||||||||||||||||||||||||||

Seq_2 1872 cagcgctgacggaagcaaaacaccagcagcagtttttccagttccgtttatccgggcaaa 1931

Seq_1 1981 ccatcgaagtgaccagcgaatacctgttccgtcatagcgataacgagctcctgcactgga 2040

||||||||||||||||||||||||||||||||||||||||||||||||||||||||||||

Seq_2 1932 ccatcgaagtgaccagcgaatacctgttccgtcatagcgataacgagctcctgcactgga 1991

Seq_1 2041 tggtggcgctggatggtaagccgctggcaagcggtgaagtgcctctggatgtcgctccac 2100

||||||||||||||||||||||||||||||||||||||||||||||||||||||||||||

Seq_2 1992 tggtggcgctggatggtaagccgctggcaagcggtgaagtgcctctggatgtcgctccac 2051

Seq_1 2101 aaggtaaacagttgattgaactgcctgaactaccgcagccggagagcgccgggcaactct 2160

||||||||||||||||||||||||||||||||||||||||||||||||||||||||||||

Seq_2 2052 aaggtaaacagttgattgaactgcctgaactaccgcagccggagagcgccgggcaactct 2111

Seq_1 2161 ggctcacagtacgcgtagtgcaaccgaacgcgaccgcatggtcagaagccgggcacatca 2220

||||||||||||||||||||||||||||||||||||||||||||||||||||||||||||

Seq_2 2112 ggctcacagtacgcgtagtgcaaccgaacgcgaccgcatggtcagaagccgggcacatca 2171

Seq_1 2221 gcgcctggcagcagtggcgtctggcggaaaacctcagtgtgacgctccccgccgcgtccc 2280

||||||||||||||||||||||||||||||||||||||||||||||||||||||||||||

Seq_2 2172 gcgcctggcagcagtggcgtctggcggaaaacctcagtgtgacgctccccgccgcgtccc 2231

Seq_1 2281 acgccatcccgcatctgaccaccagcgaaatggatttttgcatcgagctgggtaataagc 2340

||||||||||||||||||||||||||||||||||||||||||||||||||||||||||||

Seq_2 2232 acgccatcccgcatctgaccaccagcgaaatggatttttgcatcgagctgggtaataagc 2291

Seq_1 2341 gttggcaatttaaccgccagtcaggctttctttcacagatgtggattggcgataaaaaac 2400

||||||||||||||||||||||||||||||||||||||||||||||||||||||||||||

Seq_2 2292 gttggcaatttaaccgccagtcaggctttctttcacagatgtggattggcgataaaaaac 2351

Seq_1 2401 aactgctgacgccgctgcgcgatcagttcacccgtgcaccgctggataacgacattggcg 2460

||||||||||||||||||||||||||||||||||||||||||||||||||||||||||||

Seq_2 2352 aactgctgacgccgctgcgcgatcagttcacccgtgcaccgctggataacgacattggcg 2411

Seq_1 2461 taagtgaagcgacccgcattgaccctaacgcctgggtcgaacgctggaaggcggcgggcc 2520

||||||||||||||||||||||||||||||||||||||||||||||||||||||||||||

Seq_2 2412 taagtgaagcgacccgcattgaccctaacgcctgggtcgaacgctggaaggcggcgggcc 2471

Seq_1 2521 attaccaggccgaagcagcgttgttgcagtgcacggcagatacacttgctgatgcggtgc 2580

||||||||||||||||||||||||||||||||||||||||||||||||||||||||||||

Seq_2 2472 attaccaggccgaagcagcgttgttgcagtgcacggcagatacacttgctgatgcggtgc 2531

Seq_1 2581 tgattacgaccgctcacgcgtggcagcatcaggggaaaaccttatttatcagccggaaaa 2640

||||||||||||||||||||||||||||||||||||||||||||||||||||||||||||

Seq_2 2532 tgattacgaccgctcacgcgtggcagcatcaggggaaaaccttatttatcagccggaaaa 2591

Seq_1 2641 cctaccggattgatggtagtggtcaaatggcgattaccgttgatgttgaagtggcgagcg 2700

||||||||||||||||||||||||||||||||||||||||||||||||||||||||||||

Seq_2 2592 cctaccggattgatggtagtggtcaaatggcgattaccgttgatgttgaagtggcgagcg 2651

Seq_1 2701 atacaccgcatccggcgcggattggcctgaactgccagctggcgcaggtagcagagcggg 2760

||||||||||||||||||||||||||||||||||||||||||||||||||||||||||||

Seq_2 2652 atacaccgcatccggcgcggattggcctgaactgccagctggcgcaggtagcagagcggg 2711

Seq_1 2761 taaactggctcggattagggccgcaagaaaactatcccgaccgccttactgccgcctgtt 2820

||||||||||||||||||||||||||||||||||||||||||||||||||||||||||||

Seq_2 2712 taaactggctcggattagggccgcaagaaaactatcccgaccgccttactgccgcctgtt 2771

Seq_1 2821 ttgaccgctgggatctgccattgtcagacatgtataccccgtacgtcttcccgagcgaaa 2880

||||||||||||||||||||||||||||||||||||||||||||||||||||||||||||

Seq_2 2772 ttgaccgctgggatctgccattgtcagacatgtataccccgtacgtcttcccgagcgaaa 2831

Seq_1 2881 acggtctgcgctgcgggacgcgcgaattgaattatggcccacaccagtggcgcggcgact 2940

||||||||||||||||||||||||||||||||||||||||||||||||||||||||||||

Seq_2 2832 acggtctgcgctgcgggacgcgcgaattgaattatggcccacaccagtggcgcggcgact 2891

Seq_1 2941 tccagttcaacatcagccgctacagtcaacagcaactgatggaaaccagccatcgccatc 3000

||||||||||||||||||||||||||||||||||||||||||||||||||||||||||||

Seq_2 2892 tccagttcaacatcagccgctacagtcaacagcaactgatggaaaccagccatcgccatc 2951

Seq_1 3001 tgctgcacgcggaagaaggcacatggctgaatatcgacggtttccatatggggattggtg 3060

||||||||||||||||||||||||||||||||||||||||||||||||||||||||||||

Seq_2 2952 tgctgcacgcggaagaaggcacatggctgaatatcgacggtttccatatggggattggtg 3011

Seq_1 3061 gcgacgactcctggagcccgtcagtatcggcggaattaattccagctgagcgccggtcgc 3120

||||||||||||||||||||||||||||||||||||||####||||||||||||||||||

Seq_2 3012 gcgacgactcctggagcccgtcagtatcggcggaatta----cagctgagcgccggtcgc 3067

Seq_1 3121 taccattaccagttggtctggtgtcaaaaataataataaccgggcaggccatgtctgccc 3180

||||||||||||||||||||||||||||||||||||||||||||||||||||||||||||

Seq_2 3068 taccattaccagttggtctggtgtcaaaaataataataaccgggcaggccatgtctgccc 3127

Seq_1 3181 gtatttcgcgtaaggaaatccattatgtactatttcgatcagaccagtttttaatttgtg 3240

||||||||||||||||||||||||||||||||||||||||||||||||||||||||||||

Seq_2 3128 gtatttcgcgtaaggaaatccattatgtactatttcgatcagaccagtttttaatttgtg 3187

Seq_1 3241 tgtttccatgtgtccagtttggaatactcttaacctcattggaaatcgcggcataatcac 3300

||||||||||||||||||||||||||||||||||||||||||||||||||||||||||||

Seq_2 3188 tgtttccatgtgtccagtttggaatactcttaacctcattggaaatcgcggcataatcac 3247

Seq_1 3301 tggtggtatgattgatgaccgcgtcaacaatgacctttatgccatattcttcagcggctg 3360

||||||||||||||||||||||||||||||||||||||||||||||||||||||||||||

Seq_2 3248 tggtggtatgattgatgaccgcgtcaacaatgacctttatgccatattcttcagcggctg 3307

Seq_1 3361 cacacatttctttaaattcttgttcagtacctaagtaacggttgccaatttgatacgatg 3420

||||||||||||||||||||||||||||||||||||||||||||||||||||||||||||

Seq_2 3308 cacacatttctttaaattcttgttcagtacctaagtaacggttgccaatttgatacgatg 3367

Seq_1 3421 tcggctgatacagccagtaccagttcgacatgcttttatctccttgattcccttccttta 3480

||||||||||||||||||||||||||||||||||||||||||||||||||||||||||||

Seq_2 3368 tcggctgatacagccagtaccagttcgacatgcttttatctccttgattcccttccttta 3427

Seq_1 3481 cttggttaatcggagatgtctgaatggctgtatatcctgcatcatgaatatccttcatat 3540

||||||||||||||||||||||||||||||||||||||||||||||||||||||||||||

Seq_2 3428 cttggttaatcggagatgtctgaatggctgtatatcctgcatcatgaatatccttcatat 3487

Seq_1 3541 tgtgttttaacgtattgaacgaccaattccatgcatgaagaatggttccgcttttgatcg 3600

||||||||||||||||||||||||||||||||||||||||||||||||||||||||||||

Seq_2 3488 tgtgttttaacgtattgaacgaccaattccatgcatgaagaatggttccgcttttgatcg 3547

Seq_1 3601 acggtgctgtaagctcattcgatttgttcgccgtttcagcactcgcagccgccggtcctg 3660

||||||||||||||||||||||||||||||||||||||||||||||||||||||||||||

Seq_2 3548 acggtgctgtaagctcattcgatttgttcgccgtttcagcactcgcagccgccggtcctg 3607

Seq_1 3661 ccagaaccaaatgaaacagcaataaaaatccagcgaataacggcagtaaagaggttttga 3720

||||||||||||||||||||||||||||||||||||||||||||||||||||||||||||

Seq_2 3608 ccagaaccaaatgaaacagcaataaaaatccagcgaataacggcagtaaagaggttttga 3667

Seq_1 3721 atcgttttgcaaacattcttgacactccttatttgattttttgaagacttacttcggagt 3780

||||||||||||||||||||||||||||||||||||||||||||||||||||||||||||

Seq_2 3668 atcgttttgcaaacattcttgacactccttatttgattttttgaagacttacttcggagt 3727

Seq_1 3781 caaaaatccctcttacttcattcttccgcttcctcctttcaaaccgatgtgaagactgga 3840

||||||||||||||||||||||||||||||||||||||||||||||||||||||||||||

Seq_2 3728 caaaaatccctcttacttcattcttccgcttcctcctttcaaaccgatgtgaagactgga 3787

Seq_1 3841 gaattttgttaattcttgaagacgaaagggcctcgtgatacgcctatttttataggttaa 3900

||||||||||||||||||||||||||||||||||||||||||||||||||||||||||||

Seq_2 3788 gaattttgttaattcttgaagacgaaagggcctcgtgatacgcctatttttataggttaa 3847

Seq_1 3901 tgtcatgataataatggtttcttagacgtcaggtggcacttttcggggaaatgtgcgcgg 3960

||||||||||||||||||||||||||||||||||||||||||||||||||||||||||||

Seq_2 3848 tgtcatgataataatggtttcttagacgtcaggtggcacttttcggggaaatgtgcgcgg 3907

Seq_1 3961 aacccctatttgtttatttttctaaatacattcaaatatgtatccgctcatgagacaata 4020

||||||||||||||||||||||||||||||||||||||||||||||||||||||||||||

Seq_2 3908 aacccctatttgtttatttttctaaatacattcaaatatgtatccgctcatgagacaata 3967

Seq_1 4021 accctgataaatgcttcaataatattgaaaaaggaagagtatgagtattcaacatttccg 4080

||||||||||||||||||||||||||||||||||||||||||||||||||||||||||||

Seq_2 3968 accctgataaatgcttcaataatattgaaaaaggaagagtatgagtattcaacatttccg 4027

Seq_1 4081 tgtcgcccttattcccttttttgcggcattttgccttcctgtttttgctcacccagaaac 4140

||||||||||||||||||||||||||||||||||||||||||||||||||||||||||||

Seq_2 4028 tgtcgcccttattcccttttttgcggcattttgccttcctgtttttgctcacccagaaac 4087

Seq_1 4141 gctggtgaaagtaaaagatgctgaagatcagttgggtgcacgagtgggttacatcgaact 4200

||||||||||||||||||||||||||||||||||||||||||||||||||||||||||||

Seq_2 4088 gctggtgaaagtaaaagatgctgaagatcagttgggtgcacgagtgggttacatcgaact 4147

Seq_1 4201 ggatctcaacagcggtaagatccttgagagttttcgccccgaagaacgttttccaatgat 4260

||||||||||||||||||||||||||||||||||||||||||||||||||||||||||||

Seq_2 4148 ggatctcaacagcggtaagatccttgagagttttcgccccgaagaacgttttccaatgat 4207

Seq_1 4261 gagcacttttaaagttctgctatgtggcgcggtattatcccgtgttgacgccgggcaaga 4320

||||||||||||||||||||||||||||||||||||||||||||||||||||||||||||

Seq_2 4208 gagcacttttaaagttctgctatgtggcgcggtattatcccgtgttgacgccgggcaaga 4267

Seq_1 4321 gcaactcggtcgccgcatacactattctcagaatgacttggttgagtactcaccagtcac 4380

||||||||||||||||||||||||||||||||||||||||||||||||||||||||||||

Seq_2 4268 gcaactcggtcgccgcatacactattctcagaatgacttggttgagtactcaccagtcac 4327

Seq_1 4381 agaaaagcatcttacggatggcatgacagtaagagaattatgcagtgctgccataaccat 4440

||||||||||||||||||||||||||||||||||||||||||||||||||||||||||||

Seq_2 4328 agaaaagcatcttacggatggcatgacagtaagagaattatgcagtgctgccataaccat 4387

Seq_1 4441 gagtgataacactgcggccaacttacttctgacaacgatcggaggaccgaaggagctaac 4500

||||||||||||||||||||||||||||||||||||||||||||||||||||||||||||

Seq_2 4388 gagtgataacactgcggccaacttacttctgacaacgatcggaggaccgaaggagctaac 4447

Seq_1 4501 cgcttttttgcacaacatgggggatcatgtaactcgccttgatcgttgggaaccggagct 4560

||||||||||||||||||||||||||||||||||||||||||||||||||||||||||||

Seq_2 4448 cgcttttttgcacaacatgggggatcatgtaactcgccttgatcgttgggaaccggagct 4507

Seq_1 4561 gaatgaagccataccaaacgacgagcgtgacaccacgatgccggcagcaatggcaacaac 4620

||||||||||||||||||||||||||||||||||||||||||#|||||||||||||||||

Seq_2 4508 gaatgaagccataccaaacgacgagcgtgacaccacgatgcctgcagcaatggcaacaac 4567

Seq_1 4621 gttgcgcaaactattaactggcgaactacttactctagcttcccggcaacaattaataga 4680

||||||||||||||||||||||||||||||||||||||||||||||||||||||||||||

Seq_2 4568 gttgcgcaaactattaactggcgaactacttactctagcttcccggcaacaattaataga 4627

Seq_1 4681 ctggatggaggcggataaagttgcaggaccacttctgcgctcggcccttccggctggctg 4740

||||||||||||||||||||||||||||||||||||||||||||||||||||||||||||

Seq_2 4628 ctggatggaggcggataaagttgcaggaccacttctgcgctcggcccttccggctggctg 4687

Seq_1 4741 gtttattgctgataaatctggagccggtgagcgtgggtctcgcggtatcattgcagcact 4800

||||||||||||||||||||||||||||||||||||||||||||||||||||||||||||

Seq_2 4688 gtttattgctgataaatctggagccggtgagcgtgggtctcgcggtatcattgcagcact 4747

Seq_1 4801 ggggccagatggtaagccctcccgtatcgtagttatctacacgacggggagtcaggcaac 4860

||||||||||||||||||||||||||||||||||||||||||||||||||||||||||||

Seq_2 4748 ggggccagatggtaagccctcccgtatcgtagttatctacacgacggggagtcaggcaac 4807

Seq_1 4861 tatggatgaacgaaatagacagatcgctgagataggtgcctcactgattaagcattggta 4920

||||||||||||||||||||||||||||||||||||||||||||||||||||||||||||

Seq_2 4808 tatggatgaacgaaatagacagatcgctgagataggtgcctcactgattaagcattggta 4867

Seq_1 4921 actgtcagaccaagtttactcatatatactttagattgatttaaaacttcatttttaatt 4980

||||||||||||||||||||||||||||||||||||||||||||||||||||||||||||

Seq_2 4868 actgtcagaccaagtttactcatatatactttagattgatttaaaacttcatttttaatt 4927

Seq_1 4981 taaaaggatctaggtgaagatcctttttgataatctcatgaccaaaatcccttaacgtga 5040

||||||||||||||||||||||||||||||||||||||||||||||||||||||||||||

Seq_2 4928 taaaaggatctaggtgaagatcctttttgataatctcatgaccaaaatcccttaacgtga 4987

Seq_1 5041 gttttcgttccactgagcgtcagaccccgtagaaaagatcaaaggatcttcttgagatcc 5100

||||||||||||||||||||||||||||||||||||||||||||||||||||||||||||

Seq_2 4988 gttttcgttccactgagcgtcagaccccgtagaaaagatcaaaggatcttcttgagatcc 5047

Seq_1 5101 tttttttctgcgcgtaatctgctgcttgcaaacaaaaaaaccaccgctaccagcggtggt 5160

||||||||||||||||||||||||||||||||||||||||||||||||||||||||||||

Seq_2 5048 tttttttctgcgcgtaatctgctgcttgcaaacaaaaaaaccaccgctaccagcggtggt 5107

Seq_1 5161 ttgtttgccggatcaagagctaccaactctttttccgaaggtaactggcttcagcagagc 5220

||||||||||||||||||||||||||||||||||||||||||||||||||||||||||||

Seq_2 5108 ttgtttgccggatcaagagctaccaactctttttccgaaggtaactggcttcagcagagc 5167

Seq_1 5221 gcagataccaaatactgtccttctagtgtagccgtagttaggccaccacttcaagaactc 5280

||||||||||||||||||||||||||||||||||||||||||||||||||||||||||||

Seq_2 5168 gcagataccaaatactgtccttctagtgtagccgtagttaggccaccacttcaagaactc 5227

Seq_1 5281 tgtagcaccgcctacatacctcgctctgctaatcctgttaccagtggctgctgccagtgg 5340

||||||||||||||||||||||||||||||||||||||||||||||||||||||||||||

Seq_2 5228 tgtagcaccgcctacatacctcgctctgctaatcctgttaccagtggctgctgccagtgg 5287

Seq_1 5341 cgataagtcgtgtcttaccgggttggactcaagacgatagttaccggataaggcgcagcg 5400

||||||||||||||||||||||||||||||||||||||||||||||||||||||||||||

Seq_2 5288 cgataagtcgtgtcttaccgggttggactcaagacgatagttaccggataaggcgcagcg 5347

Seq_1 5401 gtcgggctgaacggggggttcgtgcacacagcccagcttggagcgaacgacctacaccga 5460

||||||||||||||||||||||||||||||||||||||||||||||||||||||||||||

Seq_2 5348 gtcgggctgaacggggggttcgtgcacacagcccagcttggagcgaacgacctacaccga 5407

Seq_1 5461 actgagatacctacagcgtgagctatgagaaagcgccacgcttcccgaagggagaaaggc 5520

||||||||||||||||||||||||||||||||||||||||||||||||||||||||||||

Seq_2 5408 actgagatacctacagcgtgagctatgagaaagcgccacgcttcccgaagggagaaaggc 5467

Seq_1 5521 ggacaggtatccggtaagcggcagggtcggaacaggagagcgcacgagggagcttccagg 5580

||||||||||||||||||||||||||||||||||||||||||||||||||||||||||||

Seq_2 5468 ggacaggtatccggtaagcggcagggtcggaacaggagagcgcacgagggagcttccagg 5527

Seq_1 5581 gggaaacgcctggtatctttatagtcctgtcgggtttcgccacctctgacttgagcgtcg 5640

||||||||||||||||||||||||||||||||||||||||||||||||||||||||||||

Seq_2 5528 gggaaacgcctggtatctttatagtcctgtcgggtttcgccacctctgacttgagcgtcg 5587

Seq_1 5641 atttttgtgatgctcgtcaggggggcggagcctatggaaaaacgccagcaacgcggcctt 5700

||||||||||||||||||||||||||||||||||||||||||||||||||||||||||||

Seq_2 5588 atttttgtgatgctcgtcaggggggcggagcctatggaaaaacgccagcaacgcggcctt 5647

Seq_1 5701 tttacggttcctggccttttgctggccttttgctcacatgttctttcctgcgttatcccc 5760

||||||||||||||||||||||||||||||||||||||||||||||||||||||||||||

Seq_2 5648 tttacggttcctggccttttgctggccttttgctcacatgttctttcctgcgttatcccc 5707

Seq_1 5761 tgattctgtggataaccgtattaccgcctttgagtgagctgataccgctcgccgcagccg 5820

||||||||||||||||||||||||||||||||||||||||||||||||||||||||||||

Seq_2 5708 tgattctgtggataaccgtattaccgcctttgagtgagctgataccgctcgccgcagccg 5767

Seq_1 5821 aacgaccgagcgcagcgagtcagtgagcgaggaagcggaagagcgcctgatgcggtattt 5880

||||||||||||||||||||||||||||||||||||||||||||||||||||||||||||

Seq_2 5768 aacgaccgagcgcagcgagtcagtgagcgaggaagcggaagagcgcctgatgcggtattt 5827

Seq_1 5881 tctccttacgcatctgtgcggtatttcacaccgcatatggtgcactctcagtacaatctg 5940

||||||||||||||||||||||||||||||||||||||||||||||||||||||||||||

Seq_2 5828 tctccttacgcatctgtgcggtatttcacaccgcatatggtgcactctcagtacaatctg 5887

Seq_1 5941 ctctgatgccgcatagttaagccagtatacactccgctatcgctacgtgactgggtcatg 6000

||||||||||||||||||||||||||||||||||||||||||||||||||||||||||||

Seq_2 5888 ctctgatgccgcatagttaagccagtatacactccgctatcgctacgtgactgggtcatg 5947

Seq_1 6001 gctgcgccccgacacccgccaacacccgctgacgcgccctgacgggcttgtctgctcccg 6060

||||||||||||||||||||||||||||||||||||||||||||||||||||||||||||

Seq_2 5948 gctgcgccccgacacccgccaacacccgctgacgcgccctgacgggcttgtctgctcccg 6007

Seq_1 6061 gcatccgcttacagacaagctgtgaccgtctccgggagctgcatgtgtcagaggttttca 6120

||||||||||||||||||||||||||||||||||||||||||||||||||||||||||||

Seq_2 6008 gcatccgcttacagacaagctgtgaccgtctccgggagctgcatgtgtcagaggttttca 6067

Seq_1 6121 ccgtcatcaccgaaacgcgcgaggcagctgcggtaaagctcatcagcgtggtcgtgaagc 6180

||||||||||||||||||||||||||||||||||||||||||||||||||||||||||||

Seq_2 6068 ccgtcatcaccgaaacgcgcgaggcagctgcggtaaagctcatcagcgtggtcgtgaagc 6127

Seq_1 6181 gattcacagatgtctgcctgttcatccgcgtccagctcgttgagtttctccagaagcgtt 6240

||||||||||||||||||||||||||||||||||||||||||||||||||||||||||||

Seq_2 6128 gattcacagatgtctgcctgttcatccgcgtccagctcgttgagtttctccagaagcgtt 6187

Seq_1 6241 aatgtctggcttctgataaagcgggccatgttaagggcggttttttcctgtttggtcact 6300

||||||||||||||||||||||||||||||||||||||||||||||||||||||||||||

Seq_2 6188 aatgtctggcttctgataaagcgggccatgttaagggcggttttttcctgtttggtcact 6247

Seq_1 6301 gatgcctccgtgtaagggggatttctgttcatgggggtaatgataccgatgaaacgagag 6360

||||||||||||||||||||||||||||||||||||||||||||||||||||||||||||

Seq_2 6248 gatgcctccgtgtaagggggatttctgttcatgggggtaatgataccgatgaaacgagag 6307

Seq_1 6361 aggatgctcacgatacgggttactgatgatgaacatgcccggttactggaacgttgtgag 6420

||||||||||||||||||||||||||||||||||||||||||||||||||||||||||||

Seq_2 6308 aggatgctcacgatacgggttactgatgatgaacatgcccggttactggaacgttgtgag 6367

Seq_1 6421 ggtaaacaactggcggtatggatgcggcgggaccagagaaaaatcactcagggtcaatgc 6480

||||||||||||||||||||||||||||||||||||||||||||||||||||||||||||

Seq_2 6368 ggtaaacaactggcggtatggatgcggcgggaccagagaaaaatcactcagggtcaatgc 6427

Seq_1 6481 cagcgcttcgttaatacagatgtaggtgttccacagggtagccagcagcatcctgcgatg 6540

||||||||||||||||||||||||||||||||||||||||||||||||||||||||||||

Seq_2 6428 cagcgcttcgttaatacagatgtaggtgttccacagggtagccagcagcatcctgcgatg 6487

Seq_1 6541 cagatccggaacataatggtgcagggcgctgacttccgcgtttccagactttacgaaaca 6600

||||||||||||||||||||||||||||||||||||||||||||||||||||||||||||

Seq_2 6488 cagatccggaacataatggtgcagggcgctgacttccgcgtttccagactttacgaaaca 6547

Seq_1 6601 cggaaaccgaagaccattcatgttgttgctcaggtcgcagacgttttgcagcagcagtcg 6660

||||||||||||||||||||||||||||||||||||||||||||||||||||||||||||

Seq_2 6548 cggaaaccgaagaccattcatgttgttgctcaggtcgcagacgttttgcagcagcagtcg 6607

Seq_1 6661 cttcacgttcgctcgcgtatcggtgattcattctgctaaccagtaaggcaaccccgccag 6720

||||||||||||||||||||||||||||||||||||||||||||||||||||||||||||

Seq_2 6608 cttcacgttcgctcgcgtatcggtgattcattctgctaaccagtaaggcaaccccgccag 6667

Seq_1 6721 cctagccgggtcctcaacgacaggagcacgatcatgcgcacccgtggccaggacccaacg 6780

||||||||||||||||||||||||||||||||||||||||||||||||||||||||||||

Seq_2 6668 cctagccgggtcctcaacgacaggagcacgatcatgcgcacccgtggccaggacccaacg 6727

Seq_1 6781 ctgcccgagatgcgccgcgtgcggctgctggagatggcggacgcgatggatatgttctgc 6840

||||||||||||||||||||||||||||||||||||||||||||||||||||||||||||

Seq_2 6728 ctgcccgagatgcgccgcgtgcggctgctggagatggcggacgcgatggatatgttctgc 6787

Seq_1 6841 caagggttggtttgcgcattcacagttctccgcaagaattgattggctccaattcttgga 6900

||||||||||||||||||||||||||||||||||||||||||||||||||||||||||||

Seq_2 6788 caagggttggtttgcgcattcacagttctccgcaagaattgattggctccaattcttgga 6847

Seq_1 6901 gtggtgaatccgttagcgaggtgccgccggcttccattcaggtcgaggtggcccggctcc 6960

||||||||||||||||||||||||||||||||||||||||||||||||||||||||||||

Seq_2 6848 gtggtgaatccgttagcgaggtgccgccggcttccattcaggtcgaggtggcccggctcc 6907

Seq_1 6961 atgcaccgcgacgcaacgcggggaggcagacaaggtatagggcggcgcctacaatccatg 7020

||||||||||||||||||||||||||||||||||||||||||||||||||||||||||||

Seq_2 6908 atgcaccgcgacgcaacgcggggaggcagacaaggtatagggcggcgcctacaatccatg 6967

Seq_1 7021 ccaacccgttccatgtgctcgccgaggcggcataaatcgccgtgacgatcagcggtccag 7080

||||||||||||||||||||||||||||||||||||||||||||||||||||||||||||

Seq_2 6968 ccaacccgttccatgtgctcgccgaggcggcataaatcgccgtgacgatcagcggtccag 7027

Seq_1 7081 tgatcgaagttaggctggtaagagccgcgagcgatccttgaagctgtccctgatggtcgt 7140

||||||||||||||||||||||||||||||||||||||||||||||||||||||||||||

Seq_2 7028 tgatcgaagttaggctggtaagagccgcgagcgatccttgaagctgtccctgatggtcgt 7087

Seq_1 7141 catctacctgcctggacagcatggcctgcaacgcgggcatcccgatgccgccggaagcga 7200

||||||||||||||||||||||||||||||||||||||||||||||||||||||||||||

Seq_2 7088 catctacctgcctggacagcatggcctgcaacgcgggcatcccgatgccgccggaagcga 7147

Seq_1 7201 gaagaatcataatggggaaggccatccagcctcgcgtcgnnnnnnnnnnnnnnnnnnnnn 7260

||||||||||||||||||||||||||||||||||||||||||||||||||||||||||||

Seq_2 7148 gaagaatcataatggggaaggccatccagcctcgcgtcgnnnnnnnnnnnnnnnnnnnnn 7207

Seq_1 7261 nnnnnnnnnnnnnnnnnnnnnnnnnnnnnnnnnnnnnnnnnnnnnnnnnnnnnnnnnnnn 7320

||||||||||||||||||||||||||||||||||||||||||||||||||||||||||||

Seq_2 7208 nnnnnnnnnnnnnnnnnnnnnnnnnnnnnnnnnnnnnnnnnnnnnnnnnnnnnnnnnnnn 7267

Seq_1 7321 nnnnnnnnnnnnnnnnnnnnnnnnnnnnngctccggcgcaaatgcagacaatatcagcat 7380

||||||||||||||||||||||||||||||||||||||||||||||||||||||||||||

Seq_2 7268 nnnnnnnnnnnnnnnnnnnnnnnnnnnnngctccggcgcaaatgcagacaatatcagcat 7327

Seq_1 7381 ccttgcagggtatgtttctctttgatgtctttttgtttgtgaagtatttcacatttatat 7440

||||||||||||||||||||||||||||||||||||||||||||||||||||||||||||

Seq_2 7328 ccttgcagggtatgtttctctttgatgtctttttgtttgtgaagtatttcacatttatat 7387

Seq_1 7441 tgtgcaacacttcacaaacttttgcaagagaaaagttttgtctgatttatgaacaaaaaa 7500

||||||||||||||||||||||||||||||||||||||||||||||||||||||||||||

Seq_2 7388 tgtgcaacacttcacaaacttttgcaagagaaaagttttgtctgatttatgaacaaaaaa 7447

Seq_1 7501 gaaaccatcattgatggtttctttcggtaagtcccgtctagccttgccctcaatggggaa 7560

||||||||||||||||||||||||||||||||||||||||||||||||||||||||||||

Seq_2 7448 gaaaccatcattgatggtttctttcggtaagtcccgtctagccttgccctcaatggggaa 7507

Seq_1 7561 gagaaccgcttaagcccgagtcattatataaaccatttagcacgtaatcaaagccaggct 7620

||||||||||||||||||||||||||||||||||||||||||||||||||||||||||||

Seq_2 7508 gagaaccgcttaagcccgagtcattatataaaccatttagcacgtaatcaaagccaggct 7567

Seq_1 7621 gattctgaccgggcacttgggcgctgccattattaaaaatcacttttgcgttggttgtat 7680

||||||||||||||||||||||||||||||||||||||||||||||||||||||||||||

Seq_2 7568 gattctgaccgggcacttgggcgctgccattattaaaaatcacttttgcgttggttgtat 7627

Seq_1 7681 ccgtgtccgcaggcagcgtcagcgtgtaaattccgtctgcatttttagtcattggttttc 7740

||||||||||||||||||||||||||||||||||||||||||||||||||||||||||||

Seq_2 7628 ccgtgtccgcaggcagcgtcagcgtgtaaattccgtctgcatttttagtcattggttttc 7687

Seq_1 7741 caggccaagatccggtcaattcaattactcggctcccatcatgtttatagatataagcat 7800

||||||||||||||||||||||||||||||||||||||||||||||||||||||||||||

Seq_2 7688 caggccaagatccggtcaattcaattactcggctcccatcatgtttatagatataagcat 7747

Seq_1 7801 ttacctggctccaatgattcggattttgatagccgatggttttggccgacgctggatctc 7860

||||||||||||||||||||||||||||||||||||||||||||||||||||||||||||

Seq_2 7748 ttacctggctccaatgattcggattttgatagccgatggttttggccgacgctggatctc 7807

Seq_1 7861 ttttaacaaaactgtatttctcggtcctcgttacaccatcactgttcgttccttttaaca 7920

||||||||||||||||||||||||||||||||||||||||||||||||||||||||||||

Seq_2 7808 ttttaacaaaactgtatttctcggtcctcgttacaccatcactgttcgttccttttaaca 7867

Seq_1 7921 tgatggtgtatgttttgccaaattggatctccttttccgattgtgaattgatctccatcc 7980

||||||||||||||||||||||||||||||||||||||||||||||||||||||||||||

Seq_2 7868 tgatggtgtatgttttgccaaattggatctccttttccgattgtgaattgatctccatcc 7927

Seq_1 7981 ttaaacgcctgtcgtctggtccattattgatttgataaacggcttttgttgtattcgcat 8040

||||||||||||||||||||||||||||||||||||||||||||||||||||||||||||

Seq_2 7928 ttaaacgcctgtcgtctggtccattattgatttgataaacggcttttgttgtattcgcat 7987

Seq_1 8041 ctgcacgcaaggtaatcgtcagttgatcattgaaagaatgtgttacacctgttttgtaat 8100

||||||||||||||||||||||||||||||||||||||||||||||||||||||||||||

Seq_2 7988 ctgcacgcaaggtaatcgtcagttgatcattgaaagaatgtgttacacctgttttgtaat 8047

Seq_1 8101 tctcaaggaaaacatgaggcgcttttgcaatatcatcaggataaagcacagctacagacc 8160

||||||||||||||||||||||||||||||||||||||||||||||||||||||||||||

Seq_2 8048 tctcaaggaaaacatgaggcgcttttgcaatatcatcaggataaagcacagctacagacc 8107

Seq_1 8161 tggcattgatcgtgcctgtcagtttaccatcgttcacttgaaatgaacccgctccagctt 8220

||||||||||||||||||||||||||||||||||||||||||||||||||||||||||||

Seq_2 8108 tggcattgatcgtgcctgtcagtttaccatcgttcacttgaaatgaacccgctccagctt 8167

Seq_1 8221 tattgtcatacctgccatcaggcaattttgttgccgtattgatagagacagaggatgaac 8280

||||||||||||||||||||||||||||||||||||||||||||||||||||||||||||

Seq_2 8168 tattgtcatacctgccatcaggcaattttgttgccgtattgatagagacagaggatgaac 8227

Seq_1 8281 ctgcatttgccagcacaacgccatgtgagccgcgctgattcataaatatctggttgtttc 8340

||||||||||||||||||||||||||||||||||||||||||||||||||||||||||||

Seq_2 8228 ctgcatttgccagcacaacgccatgtgagccgcgctgattcataaatatctggttgtttc 8287

Seq_1 8341 cattcgggttcgagagttcctcaggctgtccagccatcacattgtgaaatctattgaccg 8400

||||||||||||||||||||||||||||||||||||||||||||||||||||||||||||

Seq_2 8288 cattcgggttcgagagttcctcaggctgtccagccatcacattgtgaaatctattgaccg 8347

Seq_1 8401 cagtgatagcctgatcttcaaataaagcactcccgcgatcgcctatttggcttttccccc 8460

||||||||||||||||||||||||||||||||||||||||||||||||||||||||||||

Seq_2 8348 cagtgatagcctgatcttcaaataaagcactcccgcgatcgcctatttggcttttccccc 8407

Seq_1 8461 agatctggggaacctcacaccatttccgcctccctcaggtctggaaaagaaaagaggcgt 8520

||||||||||||||||||||||||||||||||||||||||||||||||||||||||||||

Seq_2 8408 agatctggggaacctcacaccatttccgcctccctcaggtctggaaaagaaaagaggcgt 8467

Seq_1 8521 actgcctgaacgagaagctatcaccgcccagcctaaacggatatcatcatcgctcatcca 8580

||||||||||||||||||||||||||||||||||||||||||||||||||||||||||||

Seq_2 8468 actgcctgaacgagaagctatcaccgcccagcctaaacggatatcatcatcgctcatcca 8527

Seq_1 8581 tgtcgatcgacgctctcccttatgcgactcctgcattaggaagcagcccagtagtaggtt 8640

||||||####||||||||||||||||||||||||||||||||||||||||||||||||||

Seq_2 8528 tgtcga----cgctctcccttatgcgactcctgcattaggaagcagcccagtagtaggtt 8583

Seq_1 8641 gaggccgttgagcaccgccgccgcaaggaatggtgcatgcaaggagatggcgcccaacag 8700

||||||||||||||||||||||||||||||||||||||||||||||||||||||||||||

Seq_2 8584 gaggccgttgagcaccgccgccgcaaggaatggtgcatgcaaggagatggcgcccaacag 8643

Seq_1 8701 tcccccggccacggggcctgccaccatacccacgccgaaacaagcgctcatgagcccgaa 8760

||||||||||||||||||||||||||||||||||||||||||||||||||||||||||||

Seq_2 8644 tcccccggccacggggcctgccaccatacccacgccgaaacaagcgctcatgagcccgaa 8703

Seq_1 8761 gtggcgagcccgatcttccccatcggtgatgtcggcgatataggcgccagcaaccgcacc 8820

||||||||||||||||||||||||||||||||||||||||||||||||||||||||||||

Seq_2 8704 gtggcgagcccgatcttccccatcggtgatgtcggcgatataggcgccagcaaccgcacc 8763

Seq_1 8821 tgtggcgccggtgatgccggccacgatgcgtccggcgtagaggatctggagctgtaatat 8880

||||||||||||||||||||||||||||||||||||||||||||||||||||||||||||

Seq_2 8764 tgtggcgccggtgatgccggccacgatgcgtccggcgtagaggatctggagctgtaatat 8823

Seq_1 8881 aaaaaccttcttcaactaacggggcaggttagtgacattagaaaaccgactgtaaaaagt 8940

||||||||||||||||||||||||||||||||||||||||||||||||||||||||||||

Seq_2 8824 aaaaaccttcttcaactaacggggcaggttagtgacattagaaaaccgactgtaaaaagt 8883

Seq_1 8941 acagtcggcattatctcatattataaaagccagtcattaggcctatctgacaattcctga 9000

||||||||||||||||||||||||||||||||||||||||||||||||||||||||||||

Seq_2 8884 acagtcggcattatctcatattataaaagccagtcattaggcctatctgacaattcctga 8943

Seq_1 9001 atagagttcataaacaatcctgcatgataaccatcacaaacagaatgatgtacctgtaaa 9060

||||||||||||||||||||||||||||||||||||||||||||||||||||||||||||

Seq_2 8944 atagagttcataaacaatcctgcatgataaccatcacaaacagaatgatgtacctgtaaa 9003

Seq_1 9061 gatagcggtaaatatattgaattacctttattaatgaattttcctgctgtaataatgggt 9120

||||||||||||||||||||||||||||||||||||||||||||||||||||||||||||

Seq_2 9004 gatagcggtaaatatattgaattacctttattaatgaattttcctgctgtaataatgggt 9063

Seq_1 9121 agaaggtaattactattattattgatatttaagttaaacccagtaaatgaagtccatgga 9180

||||||||||||||||||||||||||||||||||||||||||||||||||||||||||||

Seq_2 9064 agaaggtaattactattattattgatatttaagttaaacccagtaaatgaagtccatgga 9123

Seq_1 9181 ataatagaaagagaaaaagcattttcaggtataggtgttttgggaaacaatttccccgaa 9240

||||||||||||||||||||||||||||||||||||||||||||||||||||||||||||

Seq_2 9124 ataatagaaagagaaaaagcattttcaggtataggtgttttgggaaacaatttccccgaa 9183

Seq_1 9241 ccattatatttctctacatcagaaaggtataaatcataaaactctttgaagtcattcttt 9300

||||||||||||||||||||||||||||||||||||||||||||||||||||||||||||

Seq_2 9184 ccattatatttctctacatcagaaaggtataaatcataaaactctttgaagtcattcttt 9243

Seq_1 9301 acaggagtccaaataccagagaatgttttagatacaccatcaaaaattgtataaagtggc 9360

||||||||||||||||||||||||||||||||||||||||||||||||||||||||||||

Seq_2 9244 acaggagtccaaataccagagaatgttttagatacaccatcaaaaattgtataaagtggc 9303

Seq_1 9361 tctaacttatcccaataacctaactctccgtcgctattgtaaccagttctaaaagctgta 9420

||||||||||||||||||||||||||||||||||||||||||||||||||||||||||||

Seq_2 9304 tctaacttatcccaataacctaactctccgtcgctattgtaaccagttctaaaagctgta 9363

Seq_1 9421 tttgagtttatcacccttgtcactaagaaaataaatgcagggtaaaatttatatccttct 9480

||||||||||||||||||||||||||||||||||||||||||||||||||||||||||||

Seq_2 9364 tttgagtttatcacccttgtcactaagaaaataaatgcagggtaaaatttatatccttct 9423

Seq_1 9481 tgttttatgtttcggtataaaacactaatatcaatttctgtggttatactaaaagtcgtt 9540

||||||||||||||||||||||||||||||||||||||||||||||||||||||||||||

Seq_2 9424 tgttttatgtttcggtataaaacactaatatcaatttctgtggttatactaaaagtcgtt 9483

Seq_1 9541 tgttggttcaaataatgattaaatatctcttttctcttccaattgtctaaatcaatttta 9600

||||||||||||||||||||||||||||||||||||||||||||||||||||||||||||

Seq_2 9484 tgttggttcaaataatgattaaatatctcttttctcttccaattgtctaaatcaatttta 9543

Seq_1 9601 ttaaagttcatttgatatgcctcctaaatttttatctaaagtgaatttaggaggcttact 9660

||||||||||||||||||||||||||||||||||||||||||||||||||||||||||||

Seq_2 9544 ttaaagttcatttgatatgcctcctaaatttttatctaaagtgaatttaggaggcttact 9603

Seq_1 9661 tgtctgctttcttcattagaatcaatccttttttaaaagtcaatattactgtaacataaa 9720

||||||||||||||||||||||||||||||||||||||||||||||||||||||||||||

Seq_2 9604 tgtctgctttcttcattagaatcaatccttttttaaaagtcaatattactgtaacataaa 9663

Seq_1 9721 tatatattttaaaaatatcccactttatccaattttcgtttgttgaactaatgggtgctt 9780

||||||||||||||||||||||||||||||||||||||||||||||||||||||||||||

Seq_2 9664 tatatattttaaaaatatcccactttatccaattttcgtttgttgaactaatgggtgctt 9723

Seq_1 9781 tagttgaagaataaagaccacattaaaaaatgtggtcttttgtgtttttttaaaggattt 9840

||||||||||||||||||||||||||||||||||||||||||||||||||||||||||||

Seq_2 9724 tagttgaagaataaagaccacattaaaaaatgtggtcttttgtgtttttttaaaggattt 9783

Seq_1 9841 gagcgtagcgaaaaatccttttctttcttatcttgataataagggtaactattgccgatg 9900

||||||||||||||||||||||||||||||||||||||||||||||||||||||||||||

Seq_2 9784 gagcgtagcgaaaaatccttttctttcttatcttgataataagggtaactattgccgatg 9843

Seq_1 9901 ataagctgtcaaacatga 9918

||||||||||||||||||

Seq_2 9844 ataagctgtcaaacatga 9861

**Features [Seq_1]:**

lacZ_CDS : [87 : 3150 - CW]

**amyE'_CDS : [3736 : 3203 - CCW]**

**misc_feature : [3850 : 3216 - CCW]**

**bla_CDS : [4061 : 4921 - CW]**

**ori_misc_feature : [5025 : 5636 - CW]**

**'amyE_CDS : [8377 : 7550 - CCW]**

**misc_feature : [8465 : 7550 - CCW]**

**misc_feature : [8591 : 8466 - CCW]**

**cat_CDS : [9611 : 8961 - CCW]**

**cat check rev : [9688 : 9662 - CCW]**

Features [Seq_2]:

lacZ_CDS : [38 : 3097 - CW]

**amyE'_CDS complement : [3150 : 3683 - CW]**

**(null)_misc_feature complement : [3163 : 3797 - CW]**

**bla_CDS : [4008 : 4868 - CW]**

**'amyE_CDS complement : [7497 : 8324 - CW]**

**(null)_misc_feature complement : [7497 : 8534 - CW]**

**cat_CDS complement : [8904 : 9554 - CW]**

**cat check rev_misc_signal complement : [9605 : 9631 - CW]**

## Alignment of Sequence_1: pAH328 with Sequence_2: pBS3C*lux*

Similarity : 10585/10649 (99,40 %)

Seq_1 1 gaattc---------------------acagagctctcggcggccgcataa--------- 30

||||||###########################||####||||||||###|#########

Seq_2 1 gaattcgcggccgcttctagagcagtttatatactagtagcggccgctgcagtccggcaa 60

Seq_1 31 ----------------ctagtaaggtcgacaggaggactctctatgaaatttggaaactt 74

################||||||||||||||||||||||||||||||||||||||||||||

Seq_2 61 aaaagggcaaggtgtcctagtaaggtcgacaggaggactctctatgaaatttggaaactt 120

Seq_1 75 tttgcttacataccaacctccccaattttctcaaacagaggtaatgaaacgtttggttaa 134

||||||||||||||||||||||||||||||||||||||||||||||||||||||||||||

Seq_2 121 tttgcttacataccaacctccccaattttctcaaacagaggtaatgaaacgtttggttaa 180

Seq_1 135 attaggtcgcatctctgaggagtgtggttttgataccgtatggttactggagcatcattt 194

||||||||||||||||||||||||||||||||||||||||||||||||||||||||||||

Seq_2 181 attaggtcgcatctctgaggagtgtggttttgataccgtatggttactggagcatcattt 240

Seq_1 195 cacggagtttggtttgcttggtaacccttatgtcgctgctgcatatttacttggcgcgac 254

||||||||||||||||||||||||||||||||||||||||||||||||||||||||||||

Seq_2 241 cacggagtttggtttgcttggtaacccttatgtcgctgctgcatatttacttggcgcgac 300

Seq_1 255 taaaaaattgaatgtaggaactgccgctattgttcttcccacagcccatccagtacgcca 314

||||||||||||||||||||||||||||||||||||||||||||||||||||||||||||

Seq_2 301 taaaaaattgaatgtaggaactgccgctattgttcttcccacagcccatccagtacgcca 360

Seq_1 315 acttgaagatgtgaatttattggatcaaatgtcaaaaggacgatttcggtttggtatttg 374

||||||||||||||||||||||||||||||||||||||||||||||||||||||||||||

Seq_2 361 acttgaagatgtgaatttattggatcaaatgtcaaaaggacgatttcggtttggtatttg 420

Seq_1 375 ccgagggctttacaacaaggactttcgcgtattcggcacagatatgaataacagtcgcgc 434

||||||||||||||||||||||||||||||||||||||||||||||||||||||||||||

Seq_2 421 ccgagggctttacaacaaggactttcgcgtattcggcacagatatgaataacagtcgcgc 480

Seq_1 435 cttagcggaatgctggtacgggctgataaagaatggcatgacagagggatatatggaagc 494

||||||||||||||||||||||||||||||||||||||||||||||||||||||||||||

Seq_2 481 cttagcggaatgctggtacgggctgataaagaatggcatgacagagggatatatggaagc 540

Seq_1 495 tgataatgaacatatcaagttccataaggtaaaagtaaaccccgcggcgtatagcagagg 554

||||||||||||||||||||||||||||||||||||||||||||||||||||||||||||

Seq_2 541 tgataatgaacatatcaagttccataaggtaaaagtaaaccccgcggcgtatagcagagg 600

Seq_1 555 tggcgcaccggtttatgtggtggctgaatcagcttcgacgactgagtgggctgctcaatt 614

||||||||||||||||||||||||||||||||||||||||||||||||||||||||||||

Seq_2 601 tggcgcaccggtttatgtggtggctgaatcagcttcgacgactgagtgggctgctcaatt 660

Seq_1 615 tggcctaccgatgatattaagttggattataaatactaacgaaaagaaagcacaacttga 674

||||||||||||||||||||||||||||||||||||||||||||||||||||||||||||

Seq_2 661 tggcctaccgatgatattaagttggattataaatactaacgaaaagaaagcacaacttga 720

Seq_1 675 gctttataatgaagtggctcaagaatatgggcacgatattcataatatcgaccattgctt 734

||||||||||||||||||||||||||||||||||||||||||||||||||||||||||||

Seq_2 721 gctttataatgaagtggctcaagaatatgggcacgatattcataatatcgaccattgctt 780

Seq_1 735 atcatatataacatctgtagatcatgactcaattaaagcgaaagagatttgccggaaatt 794

||||||||||||||||||||||||||||||||||||||||||||||||||||||||||||

Seq_2 781 atcatatataacatctgtagatcatgactcaattaaagcgaaagagatttgccggaaatt 840

Seq_1 795 tctggggcattggtatgattcttatgtgaatgctacgactatttttgatgattcagacca 854

||||||||||||||||||||||||||||||||||||||||||||||||||||||||||||

Seq_2 841 tctggggcattggtatgattcttatgtgaatgctacgactatttttgatgattcagacca 900

Seq_1 855 aacaagaggttatgatttcaataaagggcagtggcgtgactttgtattaaaaggacataa 914

||||||||||||||||||||||||||||||||||||||||||||||||||||||||||||

Seq_2 901 aacaagaggttatgatttcaataaagggcagtggcgtgactttgtattaaaaggacataa 960

Seq_1 915 agatactaatcgccgtattgattacagttacgaaatcaatcccgtgggaacgccgcagga 974

||||||||||||||||||||||||||||||||||||||||||||||||||||||||||||

Seq_2 961 agatactaatcgccgtattgattacagttacgaaatcaatcccgtgggaacgccgcagga 1020

Seq_1 975 atgtattgacataattcaaaaagacattgatgctacaggaatatcaaatatttgttgtgg 1034

||||||||||||||||||||||||||||||||||||||||||||||||||||||||||||

Seq_2 1021 atgtattgacataattcaaaaagacattgatgctacaggaatatcaaatatttgttgtgg 1080

Seq_1 1035 atttgaagctaatggaacagtagacgaaattattgcttccatgaagctcttccagtctga 1094

||||||||||||||||||||||||||||||||||||||||||||||||||||||||||||

Seq_2 1081 atttgaagctaatggaacagtagacgaaattattgcttccatgaagctcttccagtctga 1140

Seq_1 1095 tgtcatgccatttcttaaagaaaaacaacgttcgctattatattagctaaggaggtaaag 1154

||||||||||||||||||||||||||||||||||||||||||||||||||||||||||||

Seq_2 1141 tgtcatgccatttcttaaagaaaaacaacgttcgctattatattagctaaggaggtaaag 1200

Seq_1 1155 aaatgaaatttggattgttcttccttaacttcatcaattcaacaactgttcaagaacaaa 1214

||||||||||||||||||||||||||||||||||||||||||||||||||||||||||||

Seq_2 1201 aaatgaaatttggattgttcttccttaacttcatcaattcaacaactgttcaagaacaaa 1260

Seq_1 1215 gtatagttcgcatgcaggaaataacggagtatgttgataagttgaattttgaacagattt 1274

||||||||||||||||||||||||||||||||||||||||||||||||||||||||||||

Seq_2 1261 gtatagttcgcatgcaggaaataacggagtatgttgataagttgaattttgaacagattt 1320

Seq_1 1275 tagtgtatgaaaatcatttttcagataatggtgttgtcggcgctcctctgactgtttctg 1334

||||||||||||||||||||||||||||||||||||||||||||||||||||||||||||

Seq_2 1321 tagtgtatgaaaatcatttttcagataatggtgttgtcggcgctcctctgactgtttctg 1380

Seq_1 1335 gttttctgctcggtttaacagagaaaattaaaattggttcattaaatcacatcattacaa 1394

||||||||||||||||||||||||||||||||||||||||||||||||||||||||||||

Seq_2 1381 gttttctgctcggtttaacagagaaaattaaaattggttcattaaatcacatcattacaa 1440

Seq_1 1395 ctcatcatcctgtcgccatagcggaggaagcttgcttattggatcagttaagtgaaggga 1454

||||||||||||||||||||||||||||||||||||||||||||||||||||||||||||

Seq_2 1441 ctcatcatcctgtcgccatagcggaggaagcttgcttattggatcagttaagtgaaggga 1500

Seq_1 1455 gatttattttagggtttagtgattgcgaaaaaaaagatgaaatgcatttttttaatcgcc 1514

||||||||||||||||||||||||||||||||||||||||||||||||||||||||||||

Seq_2 1501 gatttattttagggtttagtgattgcgaaaaaaaagatgaaatgcatttttttaatcgcc 1560

Seq_1 1515 cggttgaatatcaacagcaactatttgaagagtgttatgaaatcattaacgatgctttaa 1574

||||||||||||||||||||||||||||||||||||||||||||||||||||||||||||

Seq_2 1561 cggttgaatatcaacagcaactatttgaagagtgttatgaaatcattaacgatgctttaa 1620

Seq_1 1575 caacaggctattgtaatccagataacgatttttatagcttccctaaaatatctgtaaatc 1634

||||||||||||||||||||||||||||||||||||||||||||||||||||||||||||

Seq_2 1621 caacaggctattgtaatccagataacgatttttatagcttccctaaaatatctgtaaatc 1680

Seq_1 1635 cccatgcttatacgccaggcggacctcggaaatatgtaacagcaaccagtcatcatattg 1694

||||||||||||||||||||||||||||||||||||||||||||||||||||||||||||

Seq_2 1681 cccatgcttatacgccaggcggacctcggaaatatgtaacagcaaccagtcatcatattg 1740

Seq_1 1695 ttgagtgggcggccaaaaaaggtattcctctcatctttaagtgggatgattctaatgatg 1754

||||||||||||||||||||||||||||||||||||||||||||||||||||||||||||

Seq_2 1741 ttgagtgggcggccaaaaaaggtattcctctcatctttaagtgggatgattctaatgatg 1800

Seq_1 1755 ttagatatgaatatgctgaaagatataaagccgttgcggataaatatgacgttgacctat 1814

||||||||||||||||||||||||||||||||||||||||||||||||||||||||||||

Seq_2 1801 ttagatatgaatatgctgaaagatataaagccgttgcggataaatatgacgttgacctat 1860

Seq_1 1815 cagagatagaccatcagttaatgatattagttaactataacgaagatagtaataaagcta 1874

||||||||||||||||||||||||||||||||||||||||||||||||||||||||||||

Seq_2 1861 cagagatagaccatcagttaatgatattagttaactataacgaagatagtaataaagcta 1920

Seq_1 1875 aacaagagacgcgtgcatttattagtgattatgttcttgaaatgcaccctaatgaaaatt 1934

||||||||||||||||||||||||||||||||||||||||||||||||||||||||||||

Seq_2 1921 aacaagagacgcgtgcatttattagtgattatgttcttgaaatgcaccctaatgaaaatt 1980

Seq_1 1935 tcgaaaataaacttgaagaaataattgcagaaaacgctgtcggaaattatacggagtgta 1994

||||||||||||||||||||||||||||||||||||||||||||||||||||||||||||

Seq_2 1981 tcgaaaataaacttgaagaaataattgcagaaaacgctgtcggaaattatacggagtgta 2040

Seq_1 1995 taactgcggctaagttggcaattgaaaagtgtggtgcgaaaagtgtattgctgtcctttg 2054

||||||||||||||||||||||||||||||||||||||||||||||||||||||||||||

Seq_2 2041 taactgcggctaagttggcaattgaaaagtgtggtgcgaaaagtgtattgctgtcctttg 2100

Seq_1 2055 aaccaatgaatgatttgatgagccaaaaaaatgtaatcaatattgttgatgataatatta 2114

||||||||||||||||||||||||||||||||||||||||||||||||||||||||||||

Seq_2 2101 aaccaatgaatgatttgatgagccaaaaaaatgtaatcaatattgttgatgataatatta 2160

Seq_1 2115 agaagtaccacatggaatatacctaataggtaccaggaggaaggcaaatatgactaaaaa 2174

||||||||||||||||||||||||||||||||||||||||||||||||||||||||||||

Seq_2 2161 agaagtaccacatggaatatacctaataggtaccaggaggaaggcaaatatgactaaaaa 2220

Seq_1 2175 aatttcattcattattaacggccaggttgaaatctttcccgaaagtgatgatttagtgca 2234

||||||||||||||||||||||||||||||||||||||||||||||||||||||||||||

Seq_2 2221 aatttcattcattattaacggccaggttgaaatctttcccgaaagtgatgatttagtgca 2280

Seq_1 2235 atccattaattttggtgataatagtgtttacctgccaatattgaatgactctcatgtaaa 2294

||||||||||||||||||||||||||||||||||||||||||||||||||||||||||||

Seq_2 2281 atccattaattttggtgataatagtgtttacctgccaatattgaatgactctcatgtaaa 2340

Seq_1 2295 aaacattattgattgtaatggaaataacgaattacggttgcataacattgtcaattttct 2354

||||||||||||||||||||||||||||||||||||||||||||||||||||||||||||

Seq_2 2341 aaacattattgattgtaatggaaataacgaattacggttgcataacattgtcaattttct 2400

Seq_1 2355 ctatacggtagggcaaagatggaaaaatgaagaatactcaagacgcaggacatacattcg 2414

||||||||||||||||||||||||||||||||||||||||||||||||||||||||||||

Seq_2 2401 ctatacggtagggcaaagatggaaaaatgaagaatactcaagacgcaggacatacattcg 2460

Seq_1 2415 tgacttaaaaaaatatatgggatattcagaagaaatggctaagctagaggccaattggat 2474

||||||||||||||||||||||||||||||||||||||||||||||||||||||||||||

Seq_2 2461 tgacttaaaaaaatatatgggatattcagaagaaatggctaagctagaggccaattggat 2520

Seq_1 2475 atctatgattttatgttctaaaggcggcctttatgatgttgtagaaaatgaacttggttc 2534

||||||||||||||||||||||||||||||||||||||||||||||||||||||||||||

Seq_2 2521 atctatgattttatgttctaaaggcggcctttatgatgttgtagaaaatgaacttggttc 2580

Seq_1 2535 tcgccatatcatggatgaatggctacctcaggatgaaagttatgttcgggcttttccgaa 2594

||||||||||||||||||||||||||||||||||||||||||||||||||||||||||||

Seq_2 2581 tcgccatatcatggatgaatggctacctcaggatgaaagttatgttcgggcttttccgaa 2640

Seq_1 2595 aggtaaatctgtacatctgttggcaggtaatgttccattatctgggatcatgtctatatt 2654

||||||||||||||||||||||||||||||||||||||||||||||||||||||||||||

Seq_2 2641 aggtaaatctgtacatctgttggcaggtaatgttccattatctgggatcatgtctatatt 2700

Seq_1 2655 acgcgcaattttaactaagaatcagtgtattataaaaacatcgtcaaccgatccttttac 2714

||||||||||||||||||||||||||||||||||||||||||||||||||||||||||||

Seq_2 2701 acgcgcaattttaactaagaatcagtgtattataaaaacatcgtcaaccgatccttttac 2760

Seq_1 2715 cgctaatgcattagcgttaagttttattgatgtagaccctaatcatccgataacgcgctc 2774

||||||||||||||||||||||||||||||||||||||||||||||||||||||||||||

Seq_2 2761 cgctaatgcattagcgttaagttttattgatgtagaccctaatcatccgataacgcgctc 2820

Seq_1 2775 tttatctgttatatattggccccaccaaggtgatacatcactcgcaaaagaaattatgcg 2834

||||||||||||||||||||||||||||||||||||||||||||||||||||||||||||

Seq_2 2821 tttatctgttatatattggccccaccaaggtgatacatcactcgcaaaagaaattatgcg 2880

Seq_1 2835 acatgcggatgttattgtcgcttggggagggccagatgcgattaattgggcggtagagca 2894

||||||||||||||||||||||||||||||||||||||||||||||||||||||||||||

Seq_2 2881 acatgcggatgttattgtcgcttggggagggccagatgcgattaattgggcggtagagca 2940

Seq_1 2895 tgcgccatcttatgctgatgtgattaaatttggttctaaaaagagtctttgcattatcga 2954

||||||||||||||||||||||||||||||||||||||||||||||||||||||||||||

Seq_2 2941 tgcgccatcttatgctgatgtgattaaatttggttctaaaaagagtctttgcattatcga 3000

Seq_1 2955 taatcctgttgatttgacgtccgcagcgacaggtgcggctcatgatgtttgtttttacga 3014

||||||||||||||||||||||||||||||||||||||||||||||||||||||||||||

Seq_2 3001 taatcctgttgatttgacgtccgcagcgacaggtgcggctcatgatgtttgtttttacga 3060

Seq_1 3015 tcagcgagcttgtttttctgcccaaaacatatattacatgggaaatcattatgaggaatt 3074

||||||||||||||||||||||||||||||||||||||||||||||||||||||||||||

Seq_2 3061 tcagcgagcttgtttttctgcccaaaacatatattacatgggaaatcattatgaggaatt 3120

Seq_1 3075 taagttagcgttgatagaaaaacttaatctatatgcgcatatattaccgaatgccaaaaa 3134

||||||||||||||||||||||||||||||||||||||||||||||||||||||||||||

Seq_2 3121 taagttagcgttgatagaaaaacttaatctatatgcgcatatattaccgaatgccaaaaa 3180

Seq_1 3135 agattttgatgaaaaggcggcctattctttagttcaaaaagaaagcttgtttgctggatt 3194

||||||||||||||||||||||||||||||||||||||||||||||||||||||||||||

Seq_2 3181 agattttgatgaaaaggcggcctattctttagttcaaaaagaaagcttgtttgctggatt 3240

Seq_1 3195 aaaagtagaggtggatattcatcaacgttggatgattattgagtcaaatgcaggtgtgga 3254

||||||||||||||||||||||||||||||||||||||||||||||||||||||||||||

Seq_2 3241 aaaagtagaggtggatattcatcaacgttggatgattattgagtcaaatgcaggtgtgga 3300

Seq_1 3255 atttaatcaaccacttggcagatgtgtgtaccttcatcacgtcgataatattgagcaaat 3314

||||||||||||||||||||||||||||||||||||||||||||||||||||||||||||

Seq_2 3301 atttaatcaaccacttggcagatgtgtgtaccttcatcacgtcgataatattgagcaaat 3360

Seq_1 3315 attgccttatgttcaaaaaaataagacgcaaaccatatctatttttccttgggagtcatc 3374

||||||||||||||||||||||||||||||||||||||||||||||||||||||||||||

Seq_2 3361 attgccttatgttcaaaaaaataagacgcaaaccatatctatttttccttgggagtcatc 3420

Seq_1 3375 atttaaatatcgagatgcgttagcattaaaaggtgcggaaaggattgtagaagcaggaat 3434

||||||||||||||||||||||||||||||||||||||||||||||||||||||||||||

Seq_2 3421 atttaaatatcgagatgcgttagcattaaaaggtgcggaaaggattgtagaagcaggaat 3480

Seq_1 3435 gaataacatatttcgagttggtggatctcatgacggaatgagaccgttgcaacgattagt 3494

||||||||||||||||||||||||||||||||||||||||||||||||||||||||||||

Seq_2 3481 gaataacatatttcgagttggtggatctcatgacggaatgagaccgttgcaacgattagt 3540

Seq_1 3495 gacatatatttctcatgaaaggccatctaactatacggctaaggatgttgcggttgaaat 3554

||||||||||||||||||||||||||||||||||||||||||||||||||||||||||||

Seq_2 3541 gacatatatttctcatgaaaggccatctaactatacggctaaggatgttgcggttgaaat 3600

Seq_1 3555 agaacagactcgattcctggaagaagataagttccttgtatttgtcccataatggaggta 3614

||||||||||||||||||||||||||||||||||||||||||||||||||||||||||||

Seq_2 3601 agaacagactcgattcctggaagaagataagttccttgtatttgtcccataatggaggta 3660

Seq_1 3615 aaagtatggaaaatgaatcaaaatataaaaccatcgaccacgttatttgtgttgaaggaa 3674

||||||||||||||||||||||||||||||||||||||||||||||||||||||||||||

Seq_2 3661 aaagtatggaaaatgaatcaaaatataaaaccatcgaccacgttatttgtgttgaaggaa 3720

Seq_1 3675 ataaaaaaattcatgtttgggaaacgctgccagaagaaaacagcccaaagagaaagaatg 3734

||||||||||||||||||||||||||||||||||||||||||||||||||||||||||||

Seq_2 3721 ataaaaaaattcatgtttgggaaacgctgccagaagaaaacagcccaaagagaaagaatg 3780

Seq_1 3735 ccattattattgcgtctggttttgcccgcaggatggatcattttgctggtctggcggaat 3794

||||||||||||||||||||||||||||||||||||||||||||||||||||||||||||

Seq_2 3781 ccattattattgcgtctggttttgcccgcaggatggatcattttgctggtctggcggaat 3840

Seq_1 3795 atttatcgcggaatggatttcatgtgatccgctatgattcgcttcaccacgttggattga 3854

||||||||||||||||||||||||||||||||||||||||||||||||||||||||||||

Seq_2 3841 atttatcgcggaatggatttcatgtgatccgctatgattcgcttcaccacgttggattga 3900

Seq_1 3855 gttcagggacaattgatgaatttacaatgtctataggaaagcagagcttgttagcagtgg 3914

||||||||||||||||||||||||||||||||||||||||||||||||||||||||||||

Seq_2 3901 gttcagggacaattgatgaatttacaatgtctataggaaagcagagcttgttagcagtgg 3960

Seq_1 3915 ttgattggttaactacacgaaaaataaataacttcggtatgttggcttcaagcttatctg 3974

||||||||||||||||||||||||||||||||||||||||||||||||||||||||||||

Seq_2 3961 ttgattggttaactacacgaaaaataaataacttcggtatgttggcttcaagcttatctg 4020

Seq_1 3975 cgcggatagcttatgcaagcctatctgaaatcaatgcttcgtttttaatcaccgcagtcg 4034

||||||||||||||||||||||||||||||||||||||||||||||||||||||||||||

Seq_2 4021 cgcggatagcttatgcaagcctatctgaaatcaatgcttcgtttttaatcaccgcagtcg 4080

Seq_1 4035 gtgttgttaacttaagatattctcttgaaagagctttagggtttgattatctcagtctac 4094

||||||||||||||||||||||||||||||||||||||||||||||||||||||||||||

Seq_2 4081 gtgttgttaacttaagatattctcttgaaagagctttagggtttgattatctcagtctac 4140

Seq_1 4095 ccattaatgaattgccggataatctagattttgaaggccataaattgggtgctgaagtct 4154

|||||||||||||||||||||||||#||||||||||||||||||||||||||||||||||

Seq_2 4141 ccattaatgaattgccggataatcttgattttgaaggccataaattgggtgctgaagtct 4200

Seq_1 4155 ttgcgagagattgtcttgattttggttgggaagatttagcttctacaattaataacatga 4214

||||||||||||||||||||||||||||||||||||||||||||||||||||||||||||

Seq_2 4201 ttgcgagagattgtcttgattttggttgggaagatttagcttctacaattaataacatga 4260

Seq_1 4215 tgtatcttgatataccgtttattgcttttactgcaaataacgataattgggtcaagcaag 4274

||||||||||||||||||||||||||||||||||||||||||||||||||||||||||||

Seq_2 4261 tgtatcttgatataccgtttattgcttttactgcaaataacgataattgggtcaagcaag 4320

Seq_1 4275 atgaagttatcacattgttatcaaatattcgtagtaatcgatgcaagatatattctttgt 4334

||||||||||||||||||||||||||||||||||||||||||||||||||||||||||||

Seq_2 4321 atgaagttatcacattgttatcaaatattcgtagtaatcgatgcaagatatattctttgt 4380

Seq_1 4335 taggaagttcgcatgacttgagtgaaaatttagtggtcctgcgcaatttttatcaatcgg 4394

||||||||||||||||||||||||||||||||||||||||||||||||||||||||||||

Seq_2 4381 taggaagttcgcatgacttgagtgaaaatttagtggtcctgcgcaatttttatcaatcgg 4440

Seq_1 4395 ttacgaaagccgctatcgcgatggataatgatcatctggatattgatgttgatattactg 4454

||||||||||||||||||||||||||||||||||||||||||||||||||||||||||||

Seq_2 4441 ttacgaaagccgctatcgcgatggataatgatcatctggatattgatgttgatattactg 4500

Seq_1 4455 aaccgtcatttgaacatttaactattgcgacagtcaatgaacgccgaatgagaattgaga 4514

||||||||||||||||||||||||||||||||||||||||||||||||||||||||||||

Seq_2 4501 aaccgtcatttgaacatttaactattgcgacagtcaatgaacgccgaatgagaattgaga 4560

Seq_1 4515 ttgaaaatcaagcaatttctctgtcttaaagaatcctgaggaggaaaacaggtatgactt 4574

||||||||||||||||||||||||||||||||||||||||||||||||||||||||||||

Seq_2 4561 ttgaaaatcaagcaatttctctgtcttaaagaatcctgaggaggaaaacaggtatgactt 4620

Seq_1 4575 catatgttgataaacaagaaattacagcaagctcagaaattgatgatttgattttttcga 4634

||||||||||||||||||||||||||||||||||||||||||||||||||||||||||||

Seq_2 4621 catatgttgataaacaagaaattacagcaagctcagaaattgatgatttgattttttcga 4680

Seq_1 4635 gcgatccattagtgtggtcttacgacgagcaggaaaaaatcagaaagaaacttgtgcttg 4694

||||||||||||||||||||||||||||||||||||||||||||||||||||||||||||

Seq_2 4681 gcgatccattagtgtggtcttacgacgagcaggaaaaaatcagaaagaaacttgtgcttg 4740

Seq_1 4695 atgcatttcgtaatcattataaacattgtcgagaatatcgtcactactgtcaggcacaca 4754

||||||||||||||||||||||||||||||||||||||||||||||||||||||||||||

Seq_2 4741 atgcatttcgtaatcattataaacattgtcgagaatatcgtcactactgtcaggcacaca 4800

Seq_1 4755 aagtagatgacaatattacggaaattgatgacatacctgtattcccaacatcggttttta 4814

||||||||||||||||||||||||||||||||||||||||||||||||||||||||||||

Seq_2 4801 aagtagatgacaatattacggaaattgatgacatacctgtattcccaacatcggttttta 4860

Seq_1 4815 agtttactcgcttattaacttctcaggaaaacgagattgaaagttggtttaccagtagcg 4874

||||||||||||||||||||||||||||||||||||||||||||||||||||||||||||

Seq_2 4861 agtttactcgcttattaacttctcaggaaaacgagattgaaagttggtttaccagtagcg 4920

Seq_1 4875 gcacgaatggtttaaaaagtcaggtggcgcgtgacagattaagtattgagagactcttag 4934

||||||||||||||||||||||||||||||||||||||||||||||||||||||||||||

Seq_2 4921 gcacgaatggtttaaaaagtcaggtggcgcgtgacagattaagtattgagagactcttag 4980

Seq_1 4935 gctctgtgagttatggcatgaaatatgttggtagttggtttgatcatcaaatagaattag 4994

||||||||||||||||||||||||||||||||||||||||||||||||||||||||||||

Seq_2 4981 gctctgtgagttatggcatgaaatatgttggtagttggtttgatcatcaaatagaattag 5040

Seq_1 4995 tcaatttgggaccagatagatttaatgctcataatatttggtttaaatatgttatgagtt 5054

||||||||||||||||||||||||||||||||||||||||||||||||||||||||||||

Seq_2 5041 tcaatttgggaccagatagatttaatgctcataatatttggtttaaatatgttatgagtt 5100

Seq_1 5055 tggtggaattgttatatcctacgacatttaccgtaacagaagaacgaatagattttgtta 5114

||||||||||||||||||||||||||||||||||||||||||||||||||||||||||||

Seq_2 5101 tggtggaattgttatatcctacgacatttaccgtaacagaagaacgaatagattttgtta 5160

Seq_1 5115 aaacattgaatagtcttgaacgaataaaaaatcaagggaaagatctttgtcttattggtt 5174

||||||||||||||||||||||||||||||||||||||||||||||||||||||||||||

Seq_2 5161 aaacattgaatagtcttgaacgaataaaaaatcaagggaaagatctttgtcttattggtt 5220

Seq_1 5175 cgccatactttatttatttactctgccattatatgaaagataaaaaaatctcattttctg 5234

||||||||||||||||||||||||||||||||||||||||||||||||||||||||||||

Seq_2 5221 cgccatactttatttatttactctgccattatatgaaagataaaaaaatctcattttctg 5280

Seq_1 5235 gagataaaagcctttatatcataaccggaggcggctggaaaagttacgaaaaagaatctc 5294

||||||||||||||||||||||||||||||||||||||||||||||||||||||||||||

Seq_2 5281 gagataaaagcctttatatcataaccggaggcggctggaaaagttacgaaaaagaatctc 5340

Seq_1 5295 tgaaacgtgatgatttcaatcatcttttatttgatactttcaatctcagtgatattagtc 5354

||||||||||||||||||||||||||||||||||||||||||||||||||||||||||||

Seq_2 5341 tgaaacgtgatgatttcaatcatcttttatttgatactttcaatctcagtgatattagtc 5400

Seq_1 5355 agatccgagatatatttaatcaagttgaactcaacacttgtttctttgaggatgaaatgc 5414

||||||||||||||||||||||||||||||||||||||||||||||||||||||||||||

Seq_2 5401 agatccgagatatatttaatcaagttgaactcaacacttgtttctttgaggatgaaatgc 5460

Seq_1 5415 agcgtaaacatgttccgccgtgggtatatgcgcgagcgcttgatcctgaaacgttgaaac 5474

||||||||||||||||||||||||||||||||||||||||||||||||||||||||||||

Seq_2 5461 agcgtaaacatgttccgccgtgggtatatgcgcgagcgcttgatcctgaaacgttgaaac 5520

Seq_1 5475 ctgtacctgatggaacgccggggttgatgagttatatggatgcgtcagcaaccagttatc 5534

||||||||||||||||||||||||||||||||||||||||||||||||||||||||||||

Seq_2 5521 ctgtacctgatggaacgccggggttgatgagttatatggatgcgtcagcaaccagttatc 5580

Seq_1 5535 cagcatttattgttaccgatgatgtcgggataattagcagagaatatggtaagtatcccg 5594

||||||||||||||||||||||||||||||||||||||||||||||||||||||||||||

Seq_2 5581 cagcatttattgttaccgatgatgtcgggataattagcagagaatatggtaagtatcccg 5640

Seq_1 5595 gcgtgctcgttgaaattttacgtcgcgtcaatacgaggacgcagaaagggtgtgctttaa 5654

||||||||||||||||||||||||||||||||||||||||||||||||||||||||||||

Seq_2 5641 gcgtgctcgttgaaattttacgtcgcgtcaatacgaggacgcagaaagggtgtgctttaa 5700

Seq_1 5655 gcttaaccgaagcgtttgatagttgatatcctgcaggatcctgagcgccggtcgctacca 5714

|||||||||||||||||||||||||||||||#||||||||||||||||||||||||||||

Seq_2 5701 gcttaaccgaagcgtttgatagttgatatccagcaggatcctgagcgccggtcgctacca 5760

Seq_1 5715 ttaccagttggtctggtgtcaaaaataataataaccgggcaggccatgtctgcccgtatt 5774

||||||||||||||||||||||||||||||||||||||||||||||||||||||||||||

Seq_2 5761 ttaccagttggtctggtgtcaaaaataataataaccgggcaggccatgtctgcccgtatt 5820

Seq_1 5775 tcgcgtaaggaaatccattatgtactatttcgatcagaccagtttttaatttgtgtgttt 5834

||||||||||||||||||||||||||||||||||||||||||||||||||||||||||||

Seq_2 5821 tcgcgtaaggaaatccattatgtactatttcgatcagaccagtttttaatttgtgtgttt 5880

Seq_1 5835 ccatgtgtccagtttggcgcgccccatccgaccattgactgccactggattcactgcatg 5894

||||||||||||||||||||||||||||||||||||||||||||||||||||||||||||

Seq_2 5881 ccatgtgtccagtttggcgcgccccatccgaccattgactgccactggattcactgcatg 5940

Seq_1 5895 acgctgccgagacagctgacgctttcaggacagaagctgattcagcagccgctgcctgag 5954

||||||||||||||||||||||||||||||||||||||||||||||||||||||||||||

Seq_2 5941 acgctgccgagacagctgacgctttcaggacagaagctgattcagcagccgctgcctgag 6000

Seq_1 5955 ctaaaagccatgcgcagaaatgagaaaaaaatacacatcaacatgcatggatcatctggt 6014

||||||||||||||||||||||||||||||||||||||||||||||||||||||||||||

Seq_2 6001 ctaaaagccatgcgcagaaatgagaaaaaaatacacatcaacatgcatggatcatctggt 6060

Seq_1 6015 gcgcttccagtggaaaaacctgaaagaactgagattctactggaagacattcatacggag 6074

||||||||||||||||||||||||||||||||||||||||||||||||||||||||||||

Seq_2 6061 gcgcttccagtggaaaaacctgaaagaactgagattctactggaagacattcatacggag 6120

Seq_1 6075 tctggcttttcaatcagtatccgcggaacggctacgttttccttccataaagacgagggg 6134

||||||||||||||||||||||||||||||||||||||||||||||||||||||||||||

Seq_2 6121 tctggcttttcaatcagtatccgcggaacggctacgttttccttccataaagacgagggg 6180

Seq_1 6135 attgttacgctggaacgaaagagctttgacggaaaaagaacagaagcgagacattgccgc 6194

||||||||||||||||||||||||||||||||||||||||||||||||||||||||||||

Seq_2 6181 attgttacgctggaacgaaagagctttgacggaaaaagaacagaagcgagacattgccgc 6240

Seq_1 6195 atcaaggatttgcataccgtacacatgtttctcgacgcgtcatctgtggaaatctttatc 6254

||||||||||||||||||||||||||||||||||||||||||||||||||||||||||||

Seq_2 6241 atcaaggatttgcataccgtacacatgtttctcgacgcgtcatctgtggaaatctttatc 6300

Seq_1 6255 aataacggagaagaggtctttagtgcaagatattttcctttcccgggaaatcatgaagta 6314

||||||||||||||||||||||||||||||||||||||||||||||||||||||||||||

Seq_2 6301 aataacggagaagaggtctttagtgcaagatattttcctttcccgggaaatcatgaagta 6360

Seq_1 6315 acagccagtgcgaccgggaaatcctggcgtaatagcgaagaggcccgcaccgatcgccct 6374

||||||||||||||||||||||||||||||||||||||||||||||||||||||||||||

Seq_2 6361 acagccagtgcgaccgggaaatcctggcgtaatagcgaagaggcccgcaccgatcgccct 6420

Seq_1 6375 tcccaacagttgcgcagcctgaatggcgaatggcgcctgatgcggtattttctccttacg 6434

||||||||||||||||||||||||||||||||||||||||||||||||||||||||||||

Seq_2 6421 tcccaacagttgcgcagcctgaatggcgaatggcgcctgatgcggtattttctccttacg 6480

Seq_1 6435 catctgtgcggtatttcacaccgcatatggtgcactctcagtacaatctgctctgatgcc 6494

||||||||||||||||||||||||||||||||||||||||||||||||||||||||||||

Seq_2 6481 catctgtgcggtatttcacaccgcatatggtgcactctcagtacaatctgctctgatgcc 6540

Seq_1 6495 gcatagttaagccagccccgacacccgccaacacccgctgacgcgccctgacgggcttgt 6554

||||||||||||||||||||||||||||||||||||||||||||||||||||||||||||

Seq_2 6541 gcatagttaagccagccccgacacccgccaacacccgctgacgcgccctgacgggcttgt 6600

Seq_1 6555 ctgctcccggcatccgcttacagacaagctgtgaccgtctccgggagctgcatgtgtcag 6614

||||||||||||||||||||||||||||||||||||||||||||||||||||||||||||

Seq_2 6601 ctgctcccggcatccgcttacagacaagctgtgaccgtctccgggagctgcatgtgtcag 6660

Seq_1 6615 aggttttcaccgtcatcaccgaaacgcgcgagacgaaagggcctcgtgatacgcctattt 6674

||||||||||||||||||||||||||||||||||||||||||||||||||||||||||||

Seq_2 6661 aggttttcaccgtcatcaccgaaacgcgcgagacgaaagggcctcgtgatacgcctattt 6720

Seq_1 6675 ttataggttaatgtcatgataataatggtttcttagacgtcaggtggcacttttcgggga 6734

||||||||||||||||||||||||||||||||||||||||||||||||||||||||||||

Seq_2 6721 ttataggttaatgtcatgataataatggtttcttagacgtcaggtggcacttttcgggga 6780

Seq_1 6735 aatgtgcgcggaacccctatttgtttatttttctaaatacattcaaatatgtatccgctc 6794

||||||||||||||||||||||||||||||||||||||||||||||||||||||||||||

Seq_2 6781 aatgtgcgcggaacccctatttgtttatttttctaaatacattcaaatatgtatccgctc 6840

Seq_1 6795 atgagacaataaccctgataaatgcttcaataatattgaaaaaggaagagtatgagtatt 6854

||||||||||||||||||||||||||||||||||||||||||||||||||||||||||||

Seq_2 6841 atgagacaataaccctgataaatgcttcaataatattgaaaaaggaagagtatgagtatt 6900

Seq_1 6855 caacatttccgtgtcgcccttattcccttttttgcggcattttgccttcctgtttttgct 6914

||||||||||||||||||||||||||||||||||||||||||||||||||||||||||||

Seq_2 6901 caacatttccgtgtcgcccttattcccttttttgcggcattttgccttcctgtttttgct 6960

Seq_1 6915 cacccagaaacgctggtgaaagtaaaagatgctgaagatcagttgggtgcacgagtgggt 6974

||||||||||||||||||||||||||||||||||||||||||||||||||||||||||||

Seq_2 6961 cacccagaaacgctggtgaaagtaaaagatgctgaagatcagttgggtgcacgagtgggt 7020

Seq_1 6975 tacatcgaactggatctcaacagcggtaagatccttgagagttttcgccccgaagaacgt 7034

||||||||||||||||||||||||||||||||||||||||||||||||||||||||||||

Seq_2 7021 tacatcgaactggatctcaacagcggtaagatccttgagagttttcgccccgaagaacgt 7080

Seq_1 7035 tttccaatgatgagcacttttaaagttctgctatgtggcgcggtattatcccgtattgac 7094

||||||||||||||||||||||||||||||||||||||||||||||||||||||||||||

Seq_2 7081 tttccaatgatgagcacttttaaagttctgctatgtggcgcggtattatcccgtattgac 7140

Seq_1 7095 gccgggcaagagcaactcggtcgccgcatacactattctcagaatgacttggttgagtac 7154

||||||||||||||||||||||||||||||||||||||||||||||||||||||||||||

Seq_2 7141 gccgggcaagagcaactcggtcgccgcatacactattctcagaatgacttggttgagtac 7200

Seq_1 7155 tcaccagtcacagaaaagcatcttacggatggcatgacagtaagagaattatgcagtgct 7214

||||||||||||||||||||||||||||||||||||||||||||||||||||||||||||

Seq_2 7201 tcaccagtcacagaaaagcatcttacggatggcatgacagtaagagaattatgcagtgct 7260

Seq_1 7215 gccataaccatgagtgataacactgcggccaacttacttctgacaacgatcggaggaccg 7274

||||||||||||||||||||||||||||||||||||||||||||||||||||||||||||

Seq_2 7261 gccataaccatgagtgataacactgcggccaacttacttctgacaacgatcggaggaccg 7320

Seq_1 7275 aaggagctaaccgcttttttgcacaacatgggggatcatgtaactcgccttgatcgttgg 7334

||||||||||||||||||||||||||||||||||||||||||||||||||||||||||||

Seq_2 7321 aaggagctaaccgcttttttgcacaacatgggggatcatgtaactcgccttgatcgttgg 7380

Seq_1 7335 gaaccggagctgaatgaagccataccaaacgacgagcgtgacaccacgatgcctgtagca 7394

||||||||||||||||||||||||||||||||||||||||||||||||||||||||||||

Seq_2 7381 gaaccggagctgaatgaagccataccaaacgacgagcgtgacaccacgatgcctgtagca 7440

Seq_1 7395 atggcaacaacgttgcgcaaactattaactggcgaactacttactctagcttcccggcaa 7454

||||||||||||||||||||||||||||||||||||||||||||||||||||||||||||

Seq_2 7441 atggcaacaacgttgcgcaaactattaactggcgaactacttactctagcttcccggcaa 7500

Seq_1 7455 caattaatagactggatggaggcggataaagttgcaggaccacttctgcgctcggccctt 7514

||||||||||||||||||||||||||||||||||||||||||||||||||||||||||||

Seq_2 7501 caattaatagactggatggaggcggataaagttgcaggaccacttctgcgctcggccctt 7560

Seq_1 7515 ccggctggctggtttattgctgataaatctggagccggtgagcgtgggtctcgcggtatc 7574

||||||||||||||||||||||||||||||||||||||||||||||||||||||||||||

Seq_2 7561 ccggctggctggtttattgctgataaatctggagccggtgagcgtgggtctcgcggtatc 7620

Seq_1 7575 attgcagcactggggccagatggtaagccctcccgtatcgtagttatctacacgacgggg 7634

||||||||||||||||||||||||||||||||||||||||||||||||||||||||||||

Seq_2 7621 attgcagcactggggccagatggtaagccctcccgtatcgtagttatctacacgacgggg 7680

Seq_1 7635 agtcaggcaactatggatgaacgaaatagacagatcgctgagataggtgcctcactgatt 7694

||||||||||||||||||||||||||||||||||||||||||||||||||||||||||||

Seq_2 7681 agtcaggcaactatggatgaacgaaatagacagatcgctgagataggtgcctcactgatt 7740

Seq_1 7695 aagcattggtaactgtcagaccaagtttactcatatatactttagattgatttaaaactt 7754

||||||||||||||||||||||||||||||||||||||||||||||||||||||||||||

Seq_2 7741 aagcattggtaactgtcagaccaagtttactcatatatactttagattgatttaaaactt 7800

Seq_1 7755 catttttaatttaaaaggatctaggtgaagatcctttttgataatctcatgaccaaaatc 7814

||||||||||||||||||||||||||||||||||||||||||||||||||||||||||||

Seq_2 7801 catttttaatttaaaaggatctaggtgaagatcctttttgataatctcatgaccaaaatc 7860

Seq_1 7815 ccttaacgtgagttttcgttccactgagcgtcagaccccgtagaaaagatcaaaggatct 7874

||||||||||||||||||||||||||||||||||||||||||||||||||||||||||||

Seq_2 7861 ccttaacgtgagttttcgttccactgagcgtcagaccccgtagaaaagatcaaaggatct 7920

Seq_1 7875 tcttgagatcctttttttctgcgcgtaatctgctgcttgcaaacaaaaaaaccaccgcta 7934

||||||||||||||||||||||||||||||||||||||||||||||||||||||||||||

Seq_2 7921 tcttgagatcctttttttctgcgcgtaatctgctgcttgcaaacaaaaaaaccaccgcta 7980

Seq_1 7935 ccagcggtggtttgtttgccggatcaagagctaccaactctttttccgaaggtaactggc 7994

||||||||||||||||||||||||||||||||||||||||||||||||||||||||||||

Seq_2 7981 ccagcggtggtttgtttgccggatcaagagctaccaactctttttccgaaggtaactggc 8040

Seq_1 7995 ttcagcagagcgcagataccaaatactgtccttctagtgtagccgtagttaggccaccac 8054

||||||||||||||||||||||||||||||||||||||||||||||||||||||||||||

Seq_2 8041 ttcagcagagcgcagataccaaatactgtccttctagtgtagccgtagttaggccaccac 8100

Seq_1 8055 ttcaagaactctgtagcaccgcctacatacctcgctctgctaatcctgttaccagtggct 8114

||||||||||||||||||||||||||||||||||||||||||||||||||||||||||||

Seq_2 8101 ttcaagaactctgtagcaccgcctacatacctcgctctgctaatcctgttaccagtggct 8160

Seq_1 8115 gctgccagtggcgataagtcgtgtcttaccgggttggactcaagacgatagttaccggat 8174

||||||||||||||||||||||||||||||||||||||||||||||||||||||||||||

Seq_2 8161 gctgccagtggcgataagtcgtgtcttaccgggttggactcaagacgatagttaccggat 8220

Seq_1 8175 aaggcgcagcggtcgggctgaacggggggttcgtgcacacagcccagcttggagcgaacg 8234

||||||||||||||||||||||||||||||||||||||||||||||||||||||||||||

Seq_2 8221 aaggcgcagcggtcgggctgaacggggggttcgtgcacacagcccagcttggagcgaacg 8280

Seq_1 8235 acctacaccgaactgagatacctacagcgtgagctatgagaaagcgccacgcttcccgaa 8294

||||||||||||||||||||||||||||||||||||||||||||||||||||||||||||

Seq_2 8281 acctacaccgaactgagatacctacagcgtgagctatgagaaagcgccacgcttcccgaa 8340

Seq_1 8295 gggagaaaggcggacaggtatccggtaagcggcagggtcggaacaggagagcgcacgagg 8354

||||||||||||||||||||||||||||||||||||||||||||||||||||||||||||

Seq_2 8341 gggagaaaggcggacaggtatccggtaagcggcagggtcggaacaggagagcgcacgagg 8400

Seq_1 8355 gagcttccagggggaaacgcctggtatctttatagtcctgtcgggtttcgccacctctga 8414

||||||||||||||||||||||||||||||||||||||||||||||||||||||||||||

Seq_2 8401 gagcttccagggggaaacgcctggtatctttatagtcctgtcgggtttcgccacctctga 8460

Seq_1 8415 cttgagcgtcgatttttgtgatgctcgtcaggggggcggagcctatggaaaaacgccagc 8474

||||||||||||||||||||||||||||||||||||||||||||||||||||||||||||

Seq_2 8461 cttgagcgtcgatttttgtgatgctcgtcaggggggcggagcctatggaaaaacgccagc 8520

Seq_1 8475 aacgcggcctttttacggttcctggccttttgctggccttttgctcacatgttctttcct 8534

||||||||||||||||||||||||||||||||||||||||||||||||||||||||||||

Seq_2 8521 aacgcggcctttttacggttcctggccttttgctggccttttgctcacatgttctttcct 8580

Seq_1 8535 gcgttatcccctgattctgtggataaccgtattaccgcctttgagtgagctgataccgct 8594

||||||||||||||||||||||||||||||||||||||||||||||||||||||||||||

Seq_2 8581 gcgttatcccctgattctgtggataaccgtattaccgcctttgagtgagctgataccgct 8640

Seq_1 8595 cgccgcagccgaacgaccgagcgcagcgagtcagtgagcgaggaagcggaagagcgccca 8654

||||||||||||||||||||||||||||||||||||||||||||||||||||||||||||

Seq_2 8641 cgccgcagccgaacgaccgagcgcagcgagtcagtgagcgaggaagcggaagagcgccca 8700

Seq_1 8655 atacgcaaaccgcctctccccgcgcgttggccgattcattaatgcaggacagcacatgac 8714

||||||||||||||||||||||||||||||||||||||||||||||||||||||||||||

Seq_2 8701 atacgcaaaccgcctctccccgcgcgttggccgattcattaatgcaggacagcacatgac 8760

Seq_1 8715 caggagcttcgtcgccgggcttatgaagaagtggagaaaaaagagcccatcgctaacagc 8774

||||||||||||||||||||||||||||||||||||||||||||||||||||||||||||

Seq_2 8761 caggagcttcgtcgccgggcttatgaagaagtggagaaaaaagagcccatcgctaacagc 8820

Seq_1 8775 gatccgcaccgccagcattttcatatcatgccgccggttgggctgctgaatgacccgaat 8834

||||||||||||||||||||||||||||||||||||||||||||||||||||||||||||

Seq_2 8821 gatccgcaccgccagcattttcatatcatgccgccggttgggctgctgaatgacccgaat 8880

Seq_1 8835 ggcgtgatttattggaagggcagctatcatgtattctttcagtggcagccgtttcagacg 8894

||||||||||||||||||||||||||||||||||||||||||||||||||||||||||||

Seq_2 8881 ggcgtgatttattggaagggcagctatcatgtattctttcagtggcagccgtttcagacg 8940

Seq_1 8895 gggcacggcgcaaaattttgggggcattatacgacacaggatgttgtgaattggaagcgg 8954

||||||||||||||||||||||||||||||||||||||||||||||||||||||||||||

Seq_2 8941 gggcacggcgcaaaattttgggggcattatacgacacaggatgttgtgaattggaagcgg 9000

Seq_1 8955 gaagagattgcgctggctccgagtgattggtttgataaaaacggctgctactcgggcagc 9014

||||||||||||||||||||||||||||||||||||||||||||||||||||||||||||

Seq_2 9001 gaagagattgcgctggctccgagtgattggtttgataaaaacggctgctactcgggcagc 9060

Seq_1 9015 gctgtcacgaaagacgatcggctctatcttttttacacaggaaatgtcagggatcaggat 9074

||||||||||||||||||||||||||||||||||||||||||||||||||||||||||||

Seq_2 9061 gctgtcacgaaagacgatcggctctatcttttttacacaggaaatgtcagggatcaggat 9120

Seq_1 9075 ggaaatcgggaaacgtatcaatgccttgctgtttctgacgacgggctgtcctttgagaaa 9134

||||||||||||||||||||||||||||||||||||||||||||||||||||||||||||

Seq_2 9121 ggaaatcgggaaacgtatcaatgccttgctgtttctgacgacgggctgtcctttgagaaa 9180

Seq_1 9135 aagggtgtcgtcgcccgccttccggaagcgatattaacggcgcacttttcgcgatccgaa 9194

||||||||||||||||||||||||||||||||||||||||||||||||||||||||||||

Seq_2 9181 aagggtgtcgtcgcccgccttccggaagcgatattaacggcgcacttttcgcgatccgaa 9240

Seq_1 9195 gtatgggagcatgaaggcacatggtatatggtgattggtgcgcaaacagagaatttgaaa 9254

||||||||||||||||||||||||||||||||||||||||||||||||||||||||||||

Seq_2 9241 gtatgggagcatgaaggcacatggtatatggtgattggtgcgcaaacagagaatttgaaa 9300

Seq_1 9255 gggcaggctgtgttgtttgcttctgataacctgacagagtggagatttcttggcccgata 9314

||||||||||||||||||||||||||||||||||||||||||||||||||||||||||||

Seq_2 9301 gggcaggctgtgttgtttgcttctgataacctgacagagtggagatttcttggcccgata 9360

Seq_1 9315 accggcgcgggcttcaacgggctggacgattttggcgcgcctaaacggatcaaggagatg 9374

||||||||||||||||||||||||||||||||||||||||||||||||||||||||||||

Seq_2 9361 accggcgcgggcttcaacgggctggacgattttggcgcgcctaaacggatcaaggagatg 9420

Seq_1 9375 gcgcccaacagtcccccggccacggggcctgccaccatacccacgccgaaacaagcgctc 9434

||||||||||||||||||||||||||||||||||||||||||||||||||||||||||||

Seq_2 9421 gcgcccaacagtcccccggccacggggcctgccaccatacccacgccgaaacaagcgctc 9480

Seq_1 9435 atgagcccgaagtggcgagcccgatcttccccatcggtgatgtcggcgatataggcgcca 9494

||||||||||||||||||||||||||||||||||||||||||||||||||||||||||||

Seq_2 9481 atgagcccgaagtggcgagcccgatcttccccatcggtgatgtcggcgatataggcgcca 9540

Seq_1 9495 gcaaccgcacctgtggcgccggtgatgccggccacgatgcgtccggcgtagaggatctgg 9554

||||||||||||||||||||||||||||||||||||||||||||||||||||||||||||

Seq_2 9541 gcaaccgcacctgtggcgccggtgatgccggccacgatgcgtccggcgtagaggatctgg 9600

Seq_1 9555 agctgtaatataaaaaccttcttcaactaacggggcaggttagtgacattagaaaaccga 9614

||||||||||||||||||||||||||||||||||||||||||||||||||||||||||||

Seq_2 9601 agctgtaatataaaaaccttcttcaactaacggggcaggttagtgacattagaaaaccga 9660

Seq_1 9615 ctgtaaaaagtacagtcggcattatctcatattataaaagccagtcattaggcctatctg 9674

||||||||||||||||||||||||||||||||||||||||||||||||||||||||||||

Seq_2 9661 ctgtaaaaagtacagtcggcattatctcatattataaaagccagtcattaggcctatctg 9720

Seq_1 9675 acaattcctgaatagagttcataaacaatcctgcatgataaccatcacaaacagaatgat 9734

||||||||||||||||||||||||||||||||||||||||||||||||||||||||||||

Seq_2 9721 acaattcctgaatagagttcataaacaatcctgcatgataaccatcacaaacagaatgat 9780

Seq_1 9735 gtacctgtaaagatagcggtaaatatattgaattacctttattaatgaattttcctgctg 9794

||||||||||||||||||||||||||||||||||||||||||||||||||||||||||||

Seq_2 9781 gtacctgtaaagatagcggtaaatatattgaattacctttattaatgaattttcctgctg 9840

Seq_1 9795 taataatgggtagaaggtaattactattattattgatatttaagttaaacccagtaaatg 9854

||||||||||||||||||||||||||||||||||||||||||||||||||||||||||||

Seq_2 9841 taataatgggtagaaggtaattactattattattgatatttaagttaaacccagtaaatg 9900

Seq_1 9855 aagtccatggaataatagaaagagaaaaagcattttcaggtataggtgttttgggaaaca 9914

||||||||||||||||||||||||||||||||||||||||||||||||||||||||||||

Seq_2 9901 aagtccatggaataatagaaagagaaaaagcattttcaggtataggtgttttgggaaaca 9960

Seq_1 9915 atttccccgaaccattatatttctctacatcagaaaggtataaatcataaaactctttga 9974

||||||||||||||||||||||||||||||||||||||||||||||||||||||||||||

Seq_2 9961 atttccccgaaccattatatttctctacatcagaaaggtataaatcataaaactctttga 10020

Seq_1 9975 agtcattctttacaggagtccaaataccagagaatgttttagatacaccatcaaaaattg 10034

||||||||||||||||||||||||||||||||||||||||||||||||||||||||||||

Seq_2 10021 agtcattctttacaggagtccaaataccagagaatgttttagatacaccatcaaaaattg 10080

Seq_1 10035 tataaagtggctctaacttatcccaataacctaactctccgtcgctattgtaaccagttc 10094

||||||||||||||||||||||||||||||||||||||||||||||||||||||||||||

Seq_2 10081 tataaagtggctctaacttatcccaataacctaactctccgtcgctattgtaaccagttc 10140

Seq_1 10095 taaaagctgtatttgagtttatcacccttgtcactaagaaaataaatgcagggtaaaatt 10154

||||||||||||||||||||||||||||||||||||||||||||||||||||||||||||

Seq_2 10141 taaaagctgtatttgagtttatcacccttgtcactaagaaaataaatgcagggtaaaatt 10200

Seq_1 10155 tatatccttcttgttttatgtttcggtataaaacactaatatcaatttctgtggttatac 10214

||||||||||||||||||||||||||||||||||||||||||||||||||||||||||||

Seq_2 10201 tatatccttcttgttttatgtttcggtataaaacactaatatcaatttctgtggttatac 10260

Seq_1 10215 taaaagtcgtttgttggttcaaataatgattaaatatctcttttctcttccaattgtcta 10274

||||||||||||||||||||||||||||||||||||||||||||||||||||||||||||

Seq_2 10261 taaaagtcgtttgttggttcaaataatgattaaatatctcttttctcttccaattgtcta 10320

Seq_1 10275 aatcaattttattaaagttcatttgatatgcctcctaaatttttatctaaagtgaattta 10334

||||||||||||||||||||||||||||||||||||||||||||||||||||||||||||

Seq_2 10321 aatcaattttattaaagttcatttgatatgcctcctaaatttttatctaaagtgaattta 10380

Seq_1 10335 ggaggcttacttgtctgctttcttcattagaatcaatccttttttaaaagtcaatattac 10394

||||||||||||||||||||||||||||||||||||||||||||||||||||||||||||

Seq_2 10381 ggaggcttacttgtctgctttcttcattagaatcaatccttttttaaaagtcaatattac 10440

Seq_1 10395 tgtaacataaatatatattttaaaaatatcccactttatccaattttcgtttgttgaact 10454

||||||||||||||||||||||||||||||||||||||||||||||||||||||||||||

Seq_2 10441 tgtaacataaatatatattttaaaaatatcccactttatccaattttcgtttgttgaact 10500

Seq_1 10455 aatgggtgctttagttgaagaataaagaccacattaaaaaatgtggtcttttgtgttttt 10514

||||||||||||||||||||||||||||||||||||||||||||||||||||||||||||

Seq_2 10501 aatgggtgctttagttgaagaataaagaccacattaaaaaatgtggtcttttgtgttttt 10560

Seq_1 10515 ttaaaggatttgagcgtagcgaaaaatccttttctttcttatcttgataataagggtaac 10574

||||||||||||||||||||||||||||||||||||||||||||||||||||||||||||

Seq_2 10561 ttaaaggatttgagcgtagcgaaaaatccttttctttcttatcttgataataagggtaac 10620

Seq_1 10575 tattgccgatgataagctgtcaaacatga 10603

|||||||||||||||||||||||||||||

Seq_2 10621 tattgccgatgataagctgtcaaacatga 10649

**Features [Seq_1]:**

sacA(front)_CDS : [50 : 841 - CW]

**cat_CDS : [1661 : 999 - CCW]**

**MCS_misc_feature : [1957 : 2000 - CW]**

**RBS : [2002 : 2006 - CW]**

**luxA_CDS : [2014 : 3096 - CW]**

**luxB_CDS : [3113 : 4096 - CW]**

**luxC_CDS : [4120 : 5562 - CW]**

**luxD_CDS : [5576 : 6499 - CW]**

**luxE_CDS : [6524 : 7636 - CW]**

**sacA_CDS : [7821 : 8278 - CW]**

**bla_CDS : [8790 : 9662 - CW]**

Features [Seq_2]:

MCS''_misc_feature : [ : - CW]

**RFP insert _misc_feature : [25 : 30 - CW]**

**'MCS_misc_feature : [77 : 90 - CW]**

**RBS : [92 : 96 - CW]**

**luxA_CDS : [104 : 1186 - CW]**

**luxB_CDS : [1203 : 2186 - CW]**

**luxC_CDS : [2210 : 3652 - CW]**

**luxD_CDS : [3666 : 4589 - CW]**

**luxE_CDS : [4614 : 5726 - CW]**

**sacA_CDS : [5707 : 6546 - CW]**

**bla_CDS : [6880 : 7752 - CW]**

**ori_-35_signal : [7856 : 8467 - CW]**

**sacA(front)_CDS : [8743 : 9534 - CW]**

**cat_CDS : [10354 : 9692 - CCW]**

## Alignment of Sequence_1: pBS2E with Sequence_2: pAX01

Similarity : 6149/7781 (79,03 %)

Seq_1 1 ------------------------------------------------------------ 0

############################################################

Seq_2 1 actagtttggaccatttgtcatttccccctttgatttaagtgaacaagtttatccatcaa 60

Seq_1 1 ------------------------------------------------------------ 0

############################################################

Seq_2 61 ctatcttaattgagttagtttgtttatccaataaactaactttatctcatcatatacaaa 120

Seq_1 1 ------------------------------------------------------------ 0

############################################################

Seq_2 121 ataaatgtttatttcaatgttttttttagaaaatttagttataatattagatatgatact 180

Seq_1 1 ------------------------------------------------------------ 0

############################################################

Seq_2 181 tttaaatatctaattcaagcttcaaaaaacaccaacttagttcggtggataaacaaagga 240

Seq_1 1 ------------------------------------------------------------ 0

############################################################

Seq_2 241 gtggttattattcaaattgcagatcaagctttagtaaaaaaaatgaatcaaaaattaata 300

Seq_1 1 ------------------------------------------------------------ 0

############################################################

Seq_2 301 ttagatgaaattttgaagaactcccctgtctccagggcaactctctctgagattacagga 360

Seq_1 1 ------------------------------------------------------------ 0

############################################################

Seq_2 361 ttaaacaagtctactgtctcctctcaagtaaatacactgcttgaaaaagattttattttt 420

Seq_1 1 ------------------------------------------------------------ 0

############################################################

Seq_2 421 gaaattggggcagggcaatctagaggcggcagaagacctgtaatgcttgtttttaataag 480

Seq_1 1 ------------------------------------------------gaattcgcggcc 12

################################################|#|#|#####||

Seq_2 481 aatgcaggctactcgattggtattgatataggagtcgactatcttaacggaattctaacc 540

Seq_1 13 gcttctagagcatatactagtagcggccg------------------------------- 41

|##|####||#|#|||#||#|##||###################################

Seq_2 541 gacttagaaggaaatattattctcgagaagacttctgacttgtctagttcttccgctagt 600

Seq_1 42 ------------------------------------------------------------ 41

############################################################

Seq_2 601 gaagtaaaagagattttatttgcacttattcatggttttgtaacccatatgcctgagtcc 660

Seq_1 42 ------------------------------------------------------------ 41

############################################################

Seq_2 661 ccttatggtctagtcggaataggaatttgtgttccaggccttgtagatcgtcatcagcaa 720

Seq_1 42 ------------------------------------------------------------ 41

############################################################

Seq_2 721 attattttcatgcctaacttaaattggaatatcaaagatttgcagtttttaattgagagt 780

Seq_1 42 ------------------------------------------------------------ 41

############################################################

Seq_2 781 gagtttaatgttccggtttttgttgaaaatgaagctaatgcaggagcatacggtgaaaaa 840

Seq_1 42 ------------------------------------------------------------ 41

############################################################

Seq_2 841 gtatttggtatgacaaaaaactatgaaaacatcgtttacatcagtattaatatcggaatt 900

Seq_1 42 ------------------------------------------------------------ 41

############################################################

Seq_2 901 ggaactggacttgttattaacaacgaattgtataaaggtgttcagggtttttctggggaa 960

Seq_1 42 ------------------------------------------------------------ 41

############################################################

Seq_2 961 atgggtcatatgacgatagattttaatggacccaaatgcagctgtggaaatcgaggctgt 1020

Seq_1 42 ------------------------------------------------------------ 41

############################################################

Seq_2 1021 tgggaattatatgcttctgaaaaagcgttactggcttcgctctctaaagaagaaaagaat 1080

Seq_1 42 ------------------------------------------------------------ 41

############################################################

Seq_2 1081 atttctcgaaaagagattgtggaacgcgcaaataaaaatgatgtagaaatgttaaatgca 1140

Seq_1 42 ------------------------------------------------------------ 41

############################################################

Seq_2 1141 cttcaaaactttggcttttatatcggaattggattaaccaatatccttaatacatttgat 1200

Seq_1 42 ------------------------------------------------------------ 41

############################################################

Seq_2 1201 atagaagctgttatcttgagaaatcatataattgaatctcatcccattgttttaaatacg 1260

Seq_1 42 ------------------------------------------------------------ 41

############################################################

Seq_2 1261 attaaaaacgaagtttcttctagagtccattctcatttagacaataaatgtgaactattg 1320

Seq_1 42 ------------------------------------------------------------ 41

############################################################

Seq_2 1321 ccttcttcgttaggaaaaaatgcacctgctttaggagcggtttctatcgttattgattct 1380

Seq_1 42 ------------------------------------------------------------ 41

############################################################

Seq_2 1381 tttttaagtgttacccctataagttaggagctccccgggacgttcttgccattgctgcat 1440

Seq_1 42 --------------------------------------------------ctgcagagat 51

##################################################||||||||||

Seq_2 1441 aaaaaacgcccggcggcaaccgagcgttctgaattaattaatcatcgcgactgcagagat 1500

Seq_1 52 atcgatttcaagctatatttggagttgagcctcttgaaacggacaccctgtatccgaagg 111

||||||||||||||||||||||||||||||||||||||||||||||||||||||||||||

Seq_2 1501 atcgatttcaagctatatttggagttgagcctcttgaaacggacaccctgtatccgaagg 1560

Seq_1 112 atcgaaacgctgtcagctaccgcagccaaatatatgaaatgaaggattatgcaaccgtga 171

||||||||||||||||||||||||||||||||||||||||||||||||||||||||||||

Seq_2 1561 atcgaaacgctgtcagctaccgcagccaaatatatgaaatgaaggattatgcaaccgtga 1620

Seq_1 172 ttgatgtaaagacagcttcagtggaagcggtgtatcaagaagatttttatgcgcgcacgc 231

||||||||||||||||||||||||||||||||||||||||||||||||||||||||||||

Seq_2 1621 ttgatgtaaagacagcttcagtggaagcggtgtatcaagaagatttttatgcgcgcacgc 1680

Seq_1 232 cagcggtcacaagccatgagtatcagcagggcaaggcgtattttatcggcgcgcgtttgg 291

||||||||||||||||||||||||||||||||||||||||||||||||||||||||||||

Seq_2 1681 cagcggtcacaagccatgagtatcagcagggcaaggcgtattttatcggcgcgcgtttgg 1740

Seq_1 292 aggatcaatttcagcgtgatttctatgagggtctgatcacagacctgtctctctctccag 351

||||||||||||||||||||||||||||||||||||||||||||||||||||||||||||

Seq_2 1741 aggatcaatttcagcgtgatttctatgagggtctgatcacagacctgtctctctctccag 1800

Seq_1 352 tttttccggttcggcacggaaaaggcgtctccgtacaagcgaggcaggatcaggacaatg 411

||||||||||||||||||||||||||||||||||||||||||||||||||||||||||||

Seq_2 1801 tttttccggttcggcacggaaaaggcgtctccgtacaagcgaggcaggatcaggacaatg 1860

Seq_1 412 attatatttttgtcatgaatttcacggaagaaaaacagctggtcacgtttgatcagagtg 471

||||||||||||||||||||||||||||||||||||||||||||||||||||||||||||

Seq_2 1861 attatatttttgtcatgaatttcacggaagaaaaacagctggtcacgtttgatcagagtg 1920

Seq_1 472 tgaaggacataatgacaggagacatattgtcaggcgacctgacgatggaaaagtatgaag 531

||||||||||||||||||||||||||||||||||||||||||||||||||||||||||||

Seq_2 1921 tgaaggacataatgacaggagacatattgtcaggcgacctgacgatggaaaagtatgaag 1980

Seq_1 532 tgagaattgtcgtaaacacacattagcccatcattcttgaagacgaaagggcctcgtgat 591

||||||||||||||||||||||||||||||||||||||||||||||||||||||||||||

Seq_2 1981 tgagaattgtcgtaaacacacattagcccatcattcttgaagacgaaagggcctcgtgat 2040

Seq_1 592 acgcctatttttataggttaatgtcatgataataatggtttcttagacgtcaggtggcac 651

||||||||||||||||||||||||||||||||||||||||||||||||||||||||||||

Seq_2 2041 acgcctatttttataggttaatgtcatgataataatggtttcttagacgtcaggtggcac 2100

Seq_1 652 ttttcggggaaatgtgcgcggaacccctatttgtttatttttctaaatacattcaaatat 711

||||||||||||||||||||||||||||||||||||||||||||||||||||||||||||

Seq_2 2101 ttttcggggaaatgtgcgcggaacccctatttgtttatttttctaaatacattcaaatat 2160

Seq_1 712 gtatccgctcatgagacaataaccctgataaatgcttcaataatattgaaaaaggaagag 771

||||||||||||||||||||||||||||||||||||||||||||||||||||||||||||

Seq_2 2161 gtatccgctcatgagacaataaccctgataaatgcttcaataatattgaaaaaggaagag 2220

Seq_1 772 tatgagtattcaacatttccgtgtcgcccttattcccttttttgcggcattttgccttcc 831

||||||||||||||||||||||||||||||||||||||||||||||||||||||||||||

Seq_2 2221 tatgagtattcaacatttccgtgtcgcccttattcccttttttgcggcattttgccttcc 2280

Seq_1 832 tgtttttgctcacccagaaacgctggtgaaagtaaaagatgctgaagatcagttgggtgc 891

||||||||||||||||||||||||||||||||||||||||||||||||||||||||||||

Seq_2 2281 tgtttttgctcacccagaaacgctggtgaaagtaaaagatgctgaagatcagttgggtgc 2340

Seq_1 892 acgagtgggttacatcgaactggatctcaacagcggtaagatccttgagagttttcgccc 951

||||||||||||||||||||||||||||||||||||||||||||||||||||||||||||

Seq_2 2341 acgagtgggttacatcgaactggatctcaacagcggtaagatccttgagagttttcgccc 2400

Seq_1 952 cgaagaacgttttccaatgatgagcacttttaaagttctgctatgtggcgcggtattatc 1011

||||||||||||||||||||||||||||||||||||||||||||||||||||||||||||

Seq_2 2401 cgaagaacgttttccaatgatgagcacttttaaagttctgctatgtggcgcggtattatc 2460

Seq_1 1012 ccgtgttgacgccgggcaagagcaactcggtcgccgcatacactattctcagaatgactt 1071

||||||||||||||||||||||||||||||||||||||||||||||||||||||||||||

Seq_2 2461 ccgtgttgacgccgggcaagagcaactcggtcgccgcatacactattctcagaatgactt 2520

Seq_1 1072 ggttgagtactcaccagtcacagaaaagcatcttacggatggcatgacagtaagagaatt 1131

||||||||||||||||||||||||||||||||||||||||||||||||||||||||||||

Seq_2 2521 ggttgagtactcaccagtcacagaaaagcatcttacggatggcatgacagtaagagaatt 2580

Seq_1 1132 atgcagtgctgccataaccatgagtgataacactgcggccaacttacttctgacaacgat 1191

||||||||||||||||||||||||||||||||||||||||||||||||||||||||||||

Seq_2 2581 atgcagtgctgccataaccatgagtgataacactgcggccaacttacttctgacaacgat 2640

Seq_1 1192 cggaggaccgaaggagctaaccgcttttttgcacaacatgggggatcatgtaactcgcct 1251

||||||||||||||||||||||||||||||||||||||||||||||||||||||||||||

Seq_2 2641 cggaggaccgaaggagctaaccgcttttttgcacaacatgggggatcatgtaactcgcct 2700

Seq_1 1252 tgatcgttgggaaccggagctgaatgaagccataccaaacgacgagcgtgacaccacgat 1311

||||||||||||||||||||||||||||||||||||||||||||||||||||||||||||

Seq_2 2701 tgatcgttgggaaccggagctgaatgaagccataccaaacgacgagcgtgacaccacgat 2760

Seq_1 1312 gccggcagcaatggcaacaacgttgcgcaaactattaactggcgaactacttactctagc 1371

|||#||||||||||||||||||||||||||||||||||||||||||||||||||||||||

Seq_2 2761 gcctgcagcaatggcaacaacgttgcgcaaactattaactggcgaactacttactctagc 2820

Seq_1 1372 ttcccggcaacaattaatagactggatggaggcggataaagttgcaggaccacttctgcg 1431

||||||||||||||||||||||||||||||||||||||||||||||||||||||||||||

Seq_2 2821 ttcccggcaacaattaatagactggatggaggcggataaagttgcaggaccacttctgcg 2880

Seq_1 1432 ctcggcccttccggctggctggtttattgctgataaatctggagccggtgagcgtgggtc 1491

||||||||||||||||||||||||||||||||||||||||||||||||||||||||||||

Seq_2 2881 ctcggcccttccggctggctggtttattgctgataaatctggagccggtgagcgtgggtc 2940

Seq_1 1492 tcgcggtatcattgcagcactggggccagatggtaagccctcccgtatcgtagttatcta 1551

||||||||||||||||||||||||||||||||||||||||||||||||||||||||||||

Seq_2 2941 tcgcggtatcattgcagcactggggccagatggtaagccctcccgtatcgtagttatcta 3000

Seq_1 1552 cacgacggggagtcaggcaactatggatgaacgaaatagacagatcgctgagataggtgc 1611

||||||||||||||||||||||||||||||||||||||||||||||||||||||||||||

Seq_2 3001 cacgacggggagtcaggcaactatggatgaacgaaatagacagatcgctgagataggtgc 3060

Seq_1 1612 ctcactgattaagcattggtaactgtcagaccaagtttactcatatatactttagattga 1671

||||||||||||||||||||||||||||||||||||||||||||||||||||||||||||

Seq_2 3061 ctcactgattaagcattggtaactgtcagaccaagtttactcatatatactttagattga 3120

Seq_1 1672 tttaaaacttcatttttaatttaaaaggatctaggtgaagatcctttttgataatctcat 1731

||||||||||||||||||||||||||||||||||||||||||||||||||||||||||||

Seq_2 3121 tttaaaacttcatttttaatttaaaaggatctaggtgaagatcctttttgataatctcat 3180

Seq_1 1732 gaccaaaatcccttaacgtgagttttcgttccactgagcgtcagaccccgtagaaaagat 1791

||||||||||||||||||||||||||||||||||||||||||||||||||||||||||||

Seq_2 3181 gaccaaaatcccttaacgtgagttttcgttccactgagcgtcagaccccgtagaaaagat 3240

Seq_1 1792 caaaggatcttcttgagatcctttttttctgcgcgtaatctgctgcttgcaaacaaaaaa 1851

||||||||||||||||||||||||||||||||||||||||||||||||||||||||||||

Seq_2 3241 caaaggatcttcttgagatcctttttttctgcgcgtaatctgctgcttgcaaacaaaaaa 3300

Seq_1 1852 accaccgctaccagcggtggtttgtttgccggatcaagagctaccaactctttttccgaa 1911

||||||||||||||||||||||||||||||||||||||||||||||||||||||||||||

Seq_2 3301 accaccgctaccagcggtggtttgtttgccggatcaagagctaccaactctttttccgaa 3360

Seq_1 1912 ggtaactggcttcagcagagcgcagataccaaatactgtccttctagtgtagccgtagtt 1971

||||||||||||||||||||||||||||||||||||||||||||||||||||||||||||

Seq_2 3361 ggtaactggcttcagcagagcgcagataccaaatactgtccttctagtgtagccgtagtt 3420

Seq_1 1972 aggccaccacttcaagaactctgtagcaccgcctacatacctcgctctgctaatcctgtt 2031

||||||||||||||||||||||||||||||||||||||||||||||||||||||||||||

Seq_2 3421 aggccaccacttcaagaactctgtagcaccgcctacatacctcgctctgctaatcctgtt 3480

Seq_1 2032 accagtggctgctgccagtggcgataagtcgtgtcttaccgggttggactcaagacgata 2091

||||||||||||||||||||||||||||||||||||||||||||||||||||||||||||

Seq_2 3481 accagtggctgctgccagtggcgataagtcgtgtcttaccgggttggactcaagacgata 3540

Seq_1 2092 gttaccggataaggcgcagcggtcgggctgaacggggggttcgtgcacacagcccagctt 2151

||||||||||||||||||||||||||||||||||||||||||||||||||||||||||||

Seq_2 3541 gttaccggataaggcgcagcggtcgggctgaacggggggttcgtgcacacagcccagctt 3600

Seq_1 2152 ggagcgaacgacctacaccgaactgagatacctacagcgtgagcattgagaaagcgccac 2211

||||||||||||||||||||||||||||||||||||||||||||||||||||||||||||

Seq_2 3601 ggagcgaacgacctacaccgaactgagatacctacagcgtgagcattgagaaagcgccac 3660

Seq_1 2212 gcttcccgaagggagaaaggcggacaggtatccggtaagcggcagggtcggaacaggaga 2271

||||||||||||||||||||||||||||||||||||||||||||||||||||||||||||

Seq_2 3661 gcttcccgaagggagaaaggcggacaggtatccggtaagcggcagggtcggaacaggaga 3720

Seq_1 2272 gcgcacgagggagcttccagggggaaacgcctggtatctttatagtcctgtcgggtttcg 2331

||||||||||||||||||||||||||||||||||||||||||||||||||||||||||||

Seq_2 3721 gcgcacgagggagcttccagggggaaacgcctggtatctttatagtcctgtcgggtttcg 3780

Seq_1 2332 ccacctctgacttgagcgtcgatttttgtgatgctcgtcaggggggcggagcctatggaa 2391

||||||||||||||||||||||||||||||||||||||||||||||||||||||||||||

Seq_2 3781 ccacctctgacttgagcgtcgatttttgtgatgctcgtcaggggggcggagcctatggaa 3840

Seq_1 2392 aaacgccagcaacgcggcctttttacggttcctggccttttgctggccttttgctcacat 2451

||||||||||||||||||||||||||||||||||||||||||||||||||||||||||||

Seq_2 3841 aaacgccagcaacgcggcctttttacggttcctggccttttgctggccttttgctcacat 3900

Seq_1 2452 gttctttcctgcgttatcccctgattctgtggataaccgtattaccgcctttgagtgagc 2511

||||||||||||||||||||||||||||||||||||||||||||||||||||||||||||

Seq_2 3901 gttctttcctgcgttatcccctgattctgtggataaccgtattaccgcctttgagtgagc 3960

Seq_1 2512 tgataccgctcgccgcagccgaacgaccgagcgcagcgagtcagtgagcgaggaagcgga 2571

||||||||||||||||||||||||||||||||||||||||||||||||||||||||||||

Seq_2 3961 tgataccgctcgccgcagccgaacgaccgagcgcagcgagtcagtgagcgaggaagcgga 4020

Seq_1 2572 agagcgcctgatgcggtattttctccttacgcatctgtgcggtatttcacaccgcatatg 2631

||||||||||||||||||||||||||||||||||||||||||||||||||||||||||||

Seq_2 4021 agagcgcctgatgcggtattttctccttacgcatctgtgcggtatttcacaccgcatatg 4080

Seq_1 2632 gtgcactctcagtacaatctgctctgatgccgcatagttaagccagtatacactccgcta 2691

||||||||||||||||||||||||||||||||||||||||||||||||||||||||||||

Seq_2 4081 gtgcactctcagtacaatctgctctgatgccgcatagttaagccagtatacactccgcta 4140

Seq_1 2692 tcgctacgtgactgggtcatggctgcgccccgacacccgccaacacccgctgacgcgccc 2751

||||||||||||||||||||||||||||||||||||||||||||||||||||||||||||

Seq_2 4141 tcgctacgtgactgggtcatggctgcgccccgacacccgccaacacccgctgacgcgccc 4200

Seq_1 2752 tgacgggcttgtctgctcccggcatccgcttacagacaagctgtgaccgtctccgggagc 2811

||||||||||||||||||||||||||||||||||||||||||||||||||||||||||||

Seq_2 4201 tgacgggcttgtctgctcccggcatccgcttacagacaagctgtgaccgtctccgggagc 4260

Seq_1 2812 tgcatgtgtcagaggttttcaccgtcatcaccgaaacgcgcgaggcagctgcggtaaagc 2871

||||||||||||||||||||||||||||||||||||||||||||||||||||||||||||

Seq_2 4261 tgcatgtgtcagaggttttcaccgtcatcaccgaaacgcgcgaggcagctgcggtaaagc 4320

Seq_1 2872 tcatcagcgtggtcgtgaagcgattcacagatgtctgcctgttcatccgcgtccagctcg 2931

||||||||||||||||||||||||||||||||||||||||||||||||||||||||||||

Seq_2 4321 tcatcagcgtggtcgtgaagcgattcacagatgtctgcctgttcatccgcgtccagctcg 4380

Seq_1 2932 ttgagtttctccagaagcgttaatgtctggcttctgataaagcgggccatgttaagggcg 2991

||||||||||||||||||||||||||||||||||||||||||||||||||||||||||||

Seq_2 4381 ttgagtttctccagaagcgttaatgtctggcttctgataaagcgggccatgttaagggcg 4440

Seq_1 2992 gttttttcctgtttggtcacttgatgcctccgtgtaagggggaatttctgttcatggggg 3051

||||||||||||||||||||||||||||||||||||||||||||||||||||||||||||

Seq_2 4441 gttttttcctgtttggtcacttgatgcctccgtgtaagggggaatttctgttcatggggg 4500

Seq_1 3052 taatgataccgatgaaacgagagaggatgctcacgatacgggttactgatgatgaacatg 3111

||||||||||||||||||||||||||||||||||||||||||||||||||||||||||||

Seq_2 4501 taatgataccgatgaaacgagagaggatgctcacgatacgggttactgatgatgaacatg 4560

Seq_1 3112 cccggttactggaacgttgtgagggtaaacaactggcggtatggatgcggcgggaccaga 3171

||||||||||||||||||||||||||||||||||||||||||||||||||||||||||||

Seq_2 4561 cccggttactggaacgttgtgagggtaaacaactggcggtatggatgcggcgggaccaga 4620

Seq_1 3172 gaaaaatcactcagggtcaatgccagcgcttcgttaatacagatgtaggtgttccacagg 3231

||||||||||||||||||||||||||||||||||||||||||||||||||||||||||||

Seq_2 4621 gaaaaatcactcagggtcaatgccagcgcttcgttaatacagatgtaggtgttccacagg 4680

Seq_1 3232 gtagccagcagcatcctgcgatgcagatccggaacataatggtgcagggcgctgacttcc 3291

||||||||||||||||||||||||||||||||||||||||||||||||||||||||||||

Seq_2 4681 gtagccagcagcatcctgcgatgcagatccggaacataatggtgcagggcgctgacttcc 4740

Seq_1 3292 gcgtttccagactttacgaaacacggaaaccgaagaccattcatgttgttgctcaggtcg 3351

||||||||||||||||||||||||||||||||||||||||||||||||||||||||||||

Seq_2 4741 gcgtttccagactttacgaaacacggaaaccgaagaccattcatgttgttgctcaggtcg 4800

Seq_1 3352 cagacgttttgcagcagcagtcgcttcacgttcgctcgcgtatcggtgattcattctgct 3411

||||||||||||||||||||||||||||||||||||||||||||||||||||||||||||

Seq_2 4801 cagacgttttgcagcagcagtcgcttcacgttcgctcgcgtatcggtgattcattctgct 4860

Seq_1 3412 aaccagtaaggcaaccccgccagcctagccgggtcctcaacgacaggagcacgatcatgc 3471

||||||||||||||||||||||||||||||||||||||||||||||||||||||||||||

Seq_2 4861 aaccagtaaggcaaccccgccagcctagccgggtcctcaacgacaggagcacgatcatgc 4920

Seq_1 3472 gcacccgtggccaggacccaacgctgcccgagatgcgccgcgtgcggctgctggagatgg 3531

||||||||||||||||||||||||||||||||||||||||||||||||||||||||||||

Seq_2 4921 gcacccgtggccaggacccaacgctgcccgagatgcgccgcgtgcggctgctggagatgg 4980

Seq_1 3532 cggacgcgatggatatgttctgccaagggttggtttgcgcattcacagttctccgcaaga 3591

||||||||||||||||||||||||||||||||||||||||||||||||||||||||||||

Seq_2 4981 cggacgcgatggatatgttctgccaagggttggtttgcgcattcacagttctccgcaaga 5040

Seq_1 3592 attgattggctccaattcttggagtggtgaatccgttagcgaggtgccgccagcttccat 3651

|||||||||||||||||||||||||||||||||||||||||||||||||||#||||||||

Seq_2 5041 attgattggctccaattcttggagtggtgaatccgttagcgaggtgccgccggcttccat 5100

Seq_1 3652 tcaggtcgaggtggcccggctccatgcaccgcgacgcaacgcggggaggcagacaaggta 3711

||||||||||||||||||||||||||||||||||||||||||||||||||||||||||||

Seq_2 5101 tcaggtcgaggtggcccggctccatgcaccgcgacgcaacgcggggaggcagacaaggta 5160

Seq_1 3712 tagggcggcgcctacaatccatgccaacccgttccatgtgctcgccgaggcggcataaat 3771

||||||||||||||||||||||||||||||||||||||||||||||||||||||||||||

Seq_2 5161 tagggcggcgcctacaatccatgccaacccgttccatgtgctcgccgaggcggcataaat 5220

Seq_1 3772 cgccgtgacgatcagcggtccagtgatcgaagttaggctggtaagagccgcgagcgatcc 3831

||||||||||||||||||||||||||||||||||||||||||||||||||||||||||||

Seq_2 5221 cgccgtgacgatcagcggtccagtgatcgaagttaggctggtaagagccgcgagcgatcc 5280

Seq_1 3832 ttgaagctgtccctgatggtcgtcatctacctgcctggacagcatggcctgcaacgcggg 3891

||||||||||||||||||||||||||||||||||||||||||||||||||||||||||||

Seq_2 5281 ttgaagctgtccctgatggtcgtcatctacctgcctggacagcatggcctgcaacgcggg 5340

Seq_1 3892 catcccgatgccgccggaagcgagaagaatcataatggggaaggccatccagcctcgcgt 3951

||||||||||||||||||||||||||||||||||||||||||||||||||||||||||||

Seq_2 5341 catcccgatgccgccggaagcgagaagaatcataatggggaaggccatccagcctcgcgt 5400

Seq_1 3952 cgcgactaagaaaatgccgtcaaatccgctcgccatgacttcactaacgatgcctttgaa 4011

||||||||||||||||||||||||||||||||||||||||||||||||||||||||||||

Seq_2 5401 cgcgactaagaaaatgccgtcaaatccgctcgccatgacttcactaacgatgcctttgaa 5460

Seq_1 4012 aatcttcaagttcttttctactaattcaaggcgtgtctcaccaggtttttggtttgctcc 4071

||||||||||||||||||||||||||||||||||||||||||||||||||||||||||||

Seq_2 5461 aatcttcaagttcttttctactaattcaaggcgtgtctcaccaggtttttggtttgctcc 5520

Seq_1 4072 ggcgcaaatgcagacaatatcaggatggggtgatgtcaaagcttgaaaaaacgcacgtaa 4131

||||||||||||||||||||||||||||||||||||||||||||||||||||||||||||

Seq_2 5521 ggcgcaaatgcagacaatatcaggatggggtgatgtcaaagcttgaaaaaacgcacgtaa 5580

Seq_1 4132 caaaagcaaaatttatgctccatgggggagactacaaccccgatcagtggctggatcggc 4191

||||||||||||||||||||||||||||||||||||||||||||||||||||||||||||

Seq_2 5581 caaaagcaaaatttatgctccatgggggagactacaaccccgatcagtggctggatcggc 5640

Seq_1 4192 ccgatattttagctgacgatatcaaactgatgaagctttctcatacgaatacgttttctg 4251

||||||||||||||||||||||||||||||||||||||||||||||||||||||||||||

Seq_2 5641 ccgatattttagctgacgatatcaaactgatgaagctttctcatacgaatacgttttctg 5700

Seq_1 4252 tcggcatttttgcatggagcgcacttgagccggaggagggcgtatatcaatttgaatggc 4311

||||||||||||||||||||||||||||||||||||||||||||||||||||||||||||

Seq_2 5701 tcggcatttttgcatggagcgcacttgagccggaggagggcgtatatcaatttgaatggc 5760

Seq_1 4312 tggatgatatttttgagcggattcacagtataggcggccgggtcatattagcaacgccga 4371

||||||||||||||||||||||||||||||||||||||||||||||||||||||||||||

Seq_2 5761 tggatgatatttttgagcggattcacagtataggcggccgggtcatattagcaacgccga 5820

Seq_1 4372 gcggagcccgtccggcctggctgtcgcaaacctatccggaagttttgcgcgtcaatgcct 4431

||||||||||||||||||||||||||||||||||||||||||||||||||||||||||||

Seq_2 5821 gcggagcccgtccggcctggctgtcgcaaacctatccggaagttttgcgcgtcaatgcct 5880

Seq_1 4432 cccgcgtcaaacagctgcacggcggaaggcacaaccactgcctcacatctaaagtctacc 4491

||||||||||||||||||||||||||||||||||||||||||||||||||||||||||||

Seq_2 5881 cccgcgtcaaacagctgcacggcggaaggcacaaccactgcctcacatctaaagtctacc 5940

Seq_1 4492 gagaaaaaacacggcacatcaaccgcttattagcagaacgatacggacatcacccggcgc 4551

||||||||||||||||||||||||||||||||||||||||||||||||||||||||||||

Seq_2 5941 gagaaaaaacacggcacatcaaccgcttattagcagaacgatacggacatcacccggcgc 6000

Seq_1 4552 tgttaatgtggcacatttcaaacgaatacgggggagattgccactgtgaatcgatgcggc 4611

||||||||||||||||||||||||||||||||||||||||||||||||||||||||||||

Seq_2 6001 tgttaatgtggcacatttcaaacgaatacgggggagattgccactgtgaatcgatgcggc 6060

Seq_1 4612 cgctctag---------------------------------------------------- 4619

||||||||####################################################

Seq_2 6061 cgctctagagcaacgttcttgccattgctgcataaaaaacgcccggcggcaaccgagcgt 6120

Seq_1 4620 ----------------------------------------------------------ga 4621

##########################################################||

Seq_2 6121 tctgaattaattaatcatcgggaagatcttcatcaccgaaacgcggcaggcagctctaga 6180

Seq_1 4622 gttaacaagagtttgtagaaacgcaaaaaggccatccgtcaggatggccttctgcttagc 4681

||||||||||||||||||||||||||||||||||||||||||||||||||||||||||||

Seq_2 6181 gttaacaagagtttgtagaaacgcaaaaaggccatccgtcaggatggccttctgcttagc 6240

Seq_1 4682 tagagcggcggatttgtcctactcaggagagcgttcaccgacaaacaacagataaaacga 4741

||||||||||||||||||||||||||||||||||||||||||||||||||||||||||||

Seq_2 6241 tagagcggcggatttgtcctactcaggagagcgttcaccgacaaacaacagataaaacga 6300

Seq_1 4742 aaggcccagtctttcgactgagcctttcgttttatttgatgcctcaagctagagagtc-- 4799

||||||||||||||||||||||||||||||||||||||||||||||||||||||||||##

Seq_2 6301 aaggcccagtctttcgactgagcctttcgttttatttgatgcctcaagctagagagtcat 6360

Seq_1 4800 ------------------------------ctagagtctagggacctctttagctccttg 4829

##############################||||||||||||||||||||||||||||||

Seq_2 6361 taccagatctcactgcagagatccccgggtctagagtctagggacctctttagctccttg 6420

Seq_1 4830 gaagctgtcagtagtatacctaataatttatctacattccctttagtaacgtgtaacttt 4889

||||||||||||||||||||||||||||||||||||||||||||||||||||||||||||

Seq_2 6421 gaagctgtcagtagtatacctaataatttatctacattccctttagtaacgtgtaacttt 6480

Seq_1 4890 ccaaatttacaaaagcgactcatagaattatttcctcccgttaaataatagataactatt 4949

||||||||||||||||||||||||||||||||||||||||||||||||||||||||||||

Seq_2 6481 ccaaatttacaaaagcgactcatagaattatttcctcccgttaaataatagataactatt 6540

Seq_1 4950 aaaaatagacaatacttgctcataagtaacggtacttaaattgtttactttggcgtgttt 5009

||||||||||||||||||||||||||||||||||||||||||||||||||||||||||||

Seq_2 6541 aaaaatagacaatacttgctcataagtaacggtacttaaattgtttactttggcgtgttt 6600

Seq_1 5010 cattgcttgatgaaactgatttttagtaaacagttgacgatattctcgattgacccattt 5069

||||||||||||||||||||||||||||||||||||||||||||||||||||||||||||

Seq_2 6601 cattgcttgatgaaactgatttttagtaaacagttgacgatattctcgattgacccattt 6660

Seq_1 5070 tgaaacaaagtacgtatatagcttccaatatttatctggaacatctgtggtatggcgggt 5129

||||||||||||||||||||||||||||||||||||||||||||||||||||||||||||

Seq_2 6661 tgaaacaaagtacgtatatagcttccaatatttatctggaacatctgtggtatggcgggt 6720

Seq_1 5130 aagttttattaagacactgtttacttttggtttaggatgaaagcattccgctggcagctt 5189

||||||||||||||||||||||||||||||||||||||||||||||||||||||||||||

Seq_2 6721 aagttttattaagacactgtttacttttggtttaggatgaaagcattccgctggcagctt 6780

Seq_1 5190 aagcaattgctgaatcgagacttgagtgtgcaagagcaaccctagtgttcggtgaatatc 5249

||||||||||||||||||||||||||||||||||||||||||||||||||||||||||||

Seq_2 6781 aagcaattgctgaatcgagacttgagtgtgcaagagcaaccctagtgttcggtgaatatc 6840

Seq_1 5250 caaggtacgcttgtagaatccttcttcaacaatcagatagatgtcagacgcatggctttc 5309

||||||||||||||||||||||||||||||||||||||||||||||||||||||||||||

Seq_2 6841 caaggtacgcttgtagaatccttcttcaacaatcagatagatgtcagacgcatggctttc 6900

Seq_1 5310 aaaaaccacttttttaataatttgtgtgcttaaatggtaaggaatactcccaacaatttt 5369

||||||||||||||||||||||||||||||||||||||||||||||||||||||||||||

Seq_2 6901 aaaaaccacttttttaataatttgtgtgcttaaatggtaaggaatactcccaacaatttt 6960

Seq_1 5370 atacctctgtttgttagggaattgaaactgtagaatatcttggtgaattaaagtgacacg 5429

||||||||||||||||||||||||||||||||||||||||||||||||||||||||||||

Seq_2 6961 atacctctgtttgttagggaattgaaactgtagaatatcttggtgaattaaagtgacacg 7020

Seq_1 5430 agtattcagttttaatttttctgacgataagttgaatagatgactgtctaattcaataga 5489

||||||||||||||||||||||||||||||||||||||||||||||||||||||||||||

Seq_2 7021 agtattcagttttaatttttctgacgataagttgaatagatgactgtctaattcaataga 7080

Seq_1 5490 cgttacctgtttacttattttagccagtttcgtcgttaaatgccctttacctgttccaat 5549

||||||||||||||||||||||||||||||||||||||||||||||||||||||||||||

Seq_2 7081 cgttacctgtttacttattttagccagtttcgtcgttaaatgccctttacctgttccaat 7140

Seq_1 5550 ttcgtaaacggtatcggtttcttttaaattcaattgttttattatttggttgagtacttt 5609

||||||||||||||||||||||||||||||||||||||||||||||||||||||||||||

Seq_2 7141 ttcgtaaacggtatcggtttcttttaaattcaattgttttattatttggttgagtacttt 7200

Seq_1 5610 ttcactcgttaaaaagttttgagaatattttatatttttgttcatgtaatcactccttct 5669

||||||||||||||||||||||||||||||||||||||||||||||||||||||||||||

Seq_2 7201 ttcactcgttaaaaagttttgagaatattttatatttttgttcatgtaatcactccttct 7260

Seq_1 5670 taattacaaatttttagcatctaatttaacttcaattcctattatacaaaattttaagat 5729

||||||||||||||||||||||||||||||||||||||||||||||||||||||||||||

Seq_2 7261 taattacaaatttttagcatctaatttaacttcaattcctattatacaaaattttaagat 7320

Seq_1 5730 actgcactatcaacacactcttaagtttgcttctaagtcttatttccataacttctttta 5789

||||||||||||||||||||||||||||||||||||||||||||||||||||||||||||

Seq_2 7321 actgcactatcaacacactcttaagtttgcttctaagtcttatttccataacttctttta 7380

Seq_1 5790 cgtttccgccattctttgctgtttcgatttttatgatatggtgcaagtcagcacgaacac 5849

||||||||||||||||||||||||||||||||||||||||||||||||||||||||||||

Seq_2 7381 cgtttccgccattctttgctgtttcgatttttatgatatggtgcaagtcagcacgaacac 7440

Seq_1 5850 gaaccgtcttatctcccattatatctttttttgcactgattggtgtatcatttcgttttt 5909

||||||||||||||||||||||||||||||||||||||||||||||||||||||||||||

Seq_2 7441 gaaccgtcttatctcccattatatctttttttgcactgattggtgtatcatttcgttttt 7500

Seq_1 5910 cttttgtgctagaggatcaattcttgaagacgaaagggcctcgtgatacgcctattttta 5969

||||||||||||||||||||||||||||||||||||||||||||||||||||||||||||

Seq_2 7501 cttttgtgctagaggatcaattcttgaagacgaaagggcctcgtgatacgcctattttta 7560

Seq_1 5970 taggttaatgtcatgataataatggtttcttagacgtcaggtggcacttttcggggaaat 6029

||||||||||||||||||||||||||||||||||||||||||||||||||||||||||||

Seq_2 7561 taggttaatgtcatgataataatggtttcttagacgtcaggtggcacttttcggggaaat 7620

Seq_1 6030 gtgcgcggaacccctatttgtttatttttctaaatacattcaaatatgtatccgctcatg 6089

||||||||||||||||||||||||||||||||||||||||||||||||||||||||||||

Seq_2 7621 gtgcgcggaacccctatttgtttatttttctaaatacattcaaatatgtatccgctcatg 7680

Seq_1 6090 agacaataaccctgataaatgcttcaataatattgaaaaaggaagagtgcggccgcccgc 6149

||||||||||||||||||||||||||||||||||||||||||||||||||||||||||||

Seq_2 7681 agacaataaccctgataaatgcttcaataatattgaaaaaggaagagtgcggccgcccgc 7740

Seq_1 6150 gggagctccccgggacgttcttgccattgctgcataaaaaacgcccggcggcaaccgagc 6209

||||||||####################################################

Seq_2 7741 gggagctc---------------------------------------------------- 7748

Seq_1 6210 gttctgaattaatt-aatcatcgcgactgcatctttcgctaaggatgatttctg 6262

###########||###||##||#|###|##||#|||#|#||#############

Seq_2 7749 --------gg-atcccat-ttccccctttgatttttagatatc----------- 7781

**Features [Seq_1]:**

'lacA_CDS : [150 : 557 - CW]

**bla_CDS : [773 : 1633 - CW]**

**ori_misc_feature : [1737 : 2350 - CW]**

**lacA'_CDS : [4104 : 4619 - CW]**

**T1T2_misc_feature : [4776 : 4647 - CCW]**

**T1T2 : [4776 : 4647 - CCW]**

**erm_CDS : [5654 : 4917 - CCW]**

**Term_misc_feature : [6214 : 6187 - CCW]**

Features [Seq_2]:

PxylA_misc_feature complement : [7773 : 7781 - CW]

**PxylA_misc_feature complement : [7773 : 141 - CW]**

**xylR_CDS : [283 : 1407 - CW]**

**Term_misc_feature complement : [1443 : 1470 - CW]**

**'lacA_CDS : [1599 : 2006 - CW]**

**bla_CDS : [2222 : 3082 - CW]**

**lacA'_CDS : [5553 : 6068 - CW]**

**T1 T2_misc_feature complement : [6206 : 6335 - CW]**

**T1 T2_misc_signal complement : [6206 : 6335 - CW]**

**erm_CDS complement : [6508 : 7245 - CW]**

**rbs_misc_signal complement : [7759 : 7767 - CW]**

## Alignment of Sequence_1: pBS1C with Sequence_2: pDG1662

Similarity : 6038/6982 (86,48 %)

Seq_1 1 gaattcgcggccgcttctagataaggagttattttactagtagcggccgctgcagtccgg 60

||||||######################################################

Seq_2 1 gaattc------------------------------------------------------ 1

Seq_1 61 caaaaaagggcaaggtgtcaattctcatgtttgacagcttatcatcggcaatagttaccc 120

########################||||||||||||||||||||||||||||||||||||

Seq_2 2 ------------------------tcatgtttgacagcttatcatcggcaatagttaccc 42

Seq_1 121 ttattatcaagataagaaagaaaaggatttttcgctacgctcaaatcctttaaaaaaaca 180

||||||||||||||||||||||||||||||||||||||||||||||||||||||||||||

Seq_2 43 ttattatcaagataagaaagaaaaggatttttcgctacgctcaaatcctttaaaaaaaca 102

Seq_1 181 caaaagaccacattttttaatgtggtctttattcttcaactaaagcacccattagttcaa 240

||||||||||||||||||||||||||||||||||||||||||||||||||||||||||||

Seq_2 103 caaaagaccacattttttaatgtggtctttattcttcaactaaagcacccattagttcaa 162

Seq_1 241 caaacgaaaattggataaagtgggatatttttaaaatatatatttatgttacagtaatat 300

||||||||||||||||||||||||||||||||||||||||||||||||||||||||||||

Seq_2 163 caaacgaaaattggataaagtgggatatttttaaaatatatatttatgttacagtaatat 222

Seq_1 301 tgacttttaaaaaaggattgattctaatgaagaaagcagacaagtaagcctcctaaattc 360

||||||||||||||||||||||||||||||||||||||||||||||||||||||||||||

Seq_2 223 tgacttttaaaaaaggattgattctaatgaagaaagcagacaagtaagcctcctaaattc 282

Seq_1 361 actttagataaaaatttaggaggcatatcaaatgaactttaataaaattgatttagacaa 420

||||||||||||||||||||||||||||||||||||||||||||||||||||||||||||

Seq_2 283 actttagataaaaatttaggaggcatatcaaatgaactttaataaaattgatttagacaa 342

Seq_1 421 ttggaagagaaaagagatatttaatcattatttgaaccaacaaacgacttttagtataac 480

||||||||||||||||||||||||||||||||||||||||||||||||||||||||||||

Seq_2 343 ttggaagagaaaagagatatttaatcattatttgaaccaacaaacgacttttagtataac 402

Seq_1 481 cacagaaattgatattagtgttttataccgaaacataaaacaagaaggatataaatttta 540

||||||||||||||||||||||||||||||||||||||||||||||||||||||||||||

Seq_2 403 cacagaaattgatattagtgttttataccgaaacataaaacaagaaggatataaatttta 462

Seq_1 541 ccctgcatttattttcttagtgacaagggtgataaactcaaatacagcttttagaactgg 600

||||||||||||||||||||||||||||||||||||||||||||||||||||||||||||

Seq_2 463 ccctgcatttattttcttagtgacaagggtgataaactcaaatacagcttttagaactgg 522

Seq_1 601 ttacaatagcgacggagagttaggttattgggataagttagagccactttatacaatttt 660

||||||||||||||||||||||||||||||||||||||||||||||||||||||||||||

Seq_2 523 ttacaatagcgacggagagttaggttattgggataagttagagccactttatacaatttt 582

Seq_1 661 tgatggtgtatctaaaacattctctggtatttggactcctgtaaagaatgacttcaaaga 720

||||||||||||||||||||||||||||||||||||||||||||||||||||||||||||

Seq_2 583 tgatggtgtatctaaaacattctctggtatttggactcctgtaaagaatgacttcaaaga 642

Seq_1 721 gttttatgatttatacctttctgatgtagagaaatataatggttcggggaaattgtttcc 780

||||||||||||||||||||||||||||||||||||||||||||||||||||||||||||

Seq_2 643 gttttatgatttatacctttctgatgtagagaaatataatggttcggggaaattgtttcc 702

Seq_1 781 caaaacacctatacctgaaaatgctttttctctttctattattccatggacttcatttac 840

||||||||||||||||||||||||||||||||||||||||||||||||||||||||||||

Seq_2 703 caaaacacctatacctgaaaatgctttttctctttctattattccatggacttcatttac 762

Seq_1 841 tgggtttaacttaaatatcaataataatagtaattaccttctacccattattacagcagg 900

||||||||||||||||||||||||||||||||||||||||||||||||||||||||||||

Seq_2 763 tgggtttaacttaaatatcaataataatagtaattaccttctacccattattacagcagg 822

Seq_1 901 aaaattcattaataaaggtaattcaatatatttaccgctatctttacaggtacatcattc 960

||||||||||||||||||||||||||||||||||||||||||||||||||||||||||||

Seq_2 823 aaaattcattaataaaggtaattcaatatatttaccgctatctttacaggtacatcattc 882

Seq_1 961 tgtttgtgatggttatcatgcaggattgtttatgaactctattcaggaattgtcagatag 1020

||||||||||||||||||||||||||||||||||||||||||||||||||||||||||||

Seq_2 883 tgtttgtgatggttatcatgcaggattgtttatgaactctattcaggaattgtcagatag 942

Seq_1 1021 gcctaatgactggcttttataatatgagataatgccgactgtactttttacagtcggttt 1080

||||||||||||||||||||||||||||||||||||||||||||||||||||||||||||

Seq_2 943 gcctaatgactggcttttataatatgagataatgccgactgtactttttacagtcggttt 1002

Seq_1 1081 tctaatgtcactaacctgccccgttagttgaagaaggtttttatattacagctccagatc 1140

||||||||||||||||||||||||||||||||||||||||||||||||||||||||||||

Seq_2 1003 tctaatgtcactaacctgccccgttagttgaagaaggtttttatattacagctccagatc 1062

Seq_1 1141 ctctacgccggacgcatcgtggcaggcatcaccggcgccacaggtgcggttgctggcgcc 1200

|||||||||||||||||||||||#||||||||||||||||||||||||||||||||||||

Seq_2 1063 ctctacgccggacgcatcgtggccggcatcaccggcgccacaggtgcggttgctggcgcc 1122

Seq_1 1201 tatatcgccgacatcaccgatggggaagatcgggctcgccacttcgggctcatgagcgct 1260

||||||||||||||||||||||||||||||||||||||||||||||||||||||||||||

Seq_2 1123 tatatcgccgacatcaccgatggggaagatcgggctcgccacttcgggctcatgagcgct 1182

Seq_1 1261 tgtttcggcgtgggtatggtggcaggccccgtggccgggggactgttgggcgccatctcc 1320

||||||||||||||||||||||||||||||||||||||||||||||||||||||||||||

Seq_2 1183 tgtttcggcgtgggtatggtggcaggccccgtggccgggggactgttgggcgccatctcc 1242

Seq_1 1321 ttgcatgcaccattccttgcggcggcggtgctcaacggcctcaacctactactgggctgc 1380

||||||||||||||||||||||||||||||||||||||||||||||||||||||||||||

Seq_2 1243 ttgcatgcaccattccttgcggcggcggtgctcaacggcctcaacctactactgggctgc 1302

Seq_1 1381 ttcctaatgcaggagtcgcataagggagagcgtcgacatggatgagcgatgatgatatcc 1440

||||||||||||||||||||||||||||||||||||||||||||||||||||||||||||

Seq_2 1303 ttcctaatgcaggagtcgcataagggagagcgtcgacatggatgagcgatgatgatatcc 1362

Seq_1 1441 gtttaggctgggcggtgatagcttctcgttcaggcagtacgcctcttttcttttccagac 1500

||||||||||||||||||||||||||||||||||||||||||||||||||||||||||||

Seq_2 1363 gtttaggctgggcggtgatagcttctcgttcaggcagtacgcctcttttcttttccagac 1422

Seq_1 1501 ctgagggaggcggaaatggtgtgaggttcccggggaaaagccaaataggcgatcgcggga 1560

||||||||||||||||||||||||||||||||||||||||||||||||||||||||||||

Seq_2 1423 ctgagggaggcggaaatggtgtgaggttcccggggaaaagccaaataggcgatcgcggga 1482

Seq_1 1561 gtgctttatttgaagatcaggctatcactgcggtcaatagatttcacaatgtgatggctg 1620

||||||||||||||||||||||||||||||||||||||||||||||||||||||||||||

Seq_2 1483 gtgctttatttgaagatcaggctatcactgcggtcaatagatttcacaatgtgatggctg 1542

Seq_1 1621 gacagcctgaggaactctcgaacccgaatggaaacaaccagatatttatgaatcagcgcg 1680

||||||||||||||||||||||||||||||||||||||||||||||||||||||||||||

Seq_2 1543 gacagcctgaggaactctcgaacccgaatggaaacaaccagatatttatgaatcagcgcg 1602

Seq_1 1681 gctcacatggcgttgtgctggcaaatgcaggttcatcctctgtctctatcaatacggcaa 1740

||||||||||||||||||||||||||||||||||||||||||||||||||||||||||||

Seq_2 1603 gctcacatggcgttgtgctggcaaatgcaggttcatcctctgtctctatcaatacggcaa 1662

Seq_1 1741 caaaattgcctgatggcaggtatgacaataaagctggagcgggttcatttcaagtgaacg 1800

||||||||||||||||||||||||||||||||||||||||||||||||||||||||||||

Seq_2 1663 caaaattgcctgatggcaggtatgacaataaagctggagcgggttcatttcaagtgaacg 1722

Seq_1 1801 atggtaaactgacaggcacgatcaatgccaggtctgtagctgtgctttatcctgatgata 1860

||||||||||||||||||||||||||||||||||||||||||||||||||||||||||||

Seq_2 1723 atggtaaactgacaggcacgatcaatgccaggtctgtagctgtgctttatcctgatgata 1782

Seq_1 1861 ttgcaaaagcgcctcatgttttccttgagaattacaaaacaggtgtaacacattctttca 1920

||||||||||||||||||||||||||||||||||||||||||||||||||||||||||||

Seq_2 1783 ttgcaaaagcgcctcatgttttccttgagaattacaaaacaggtgtaacacattctttca 1842

Seq_1 1921 atgatcaactgacgattaccttgcgtgcagatgcgaatacaacaaaagccgtttatcaaa 1980

||||||||||||||||||||||||||||||||||||||||||||||||||||||||||||

Seq_2 1843 atgatcaactgacgattaccttgcgtgcagatgcgaatacaacaaaagccgtttatcaaa 1902

Seq_1 1981 tcaataatggaccagacgacaggcgtttaaggatggagatcaattcacaatcggaaaagg 2040

||||||||||||||||||||||||||||||||||||||||||||||||||||||||||||

Seq_2 1903 tcaataatggaccagacgacaggcgtttaaggatggagatcaattcacaatcggaaaagg 1962

Seq_1 2041 agatccaatttggcaaaacatacaccatcatgttaaaaggaacgaacagtgatggtgtaa 2100

||||||||||||||||||||||||||||||||||||||||||||||||||||||||||||

Seq_2 1963 agatccaatttggcaaaacatacaccatcatgttaaaaggaacgaacagtgatggtgtaa 2022

Seq_1 2101 cgaggaccgagaaatacagttttgttaaaagagatccagcgtcggccaaaaccatcggct 2160

||||||||||||||||||||||||||||||||||||||||||||||||||||||||||||

Seq_2 2023 cgaggaccgagaaatacagttttgttaaaagagatccagcgtcggccaaaaccatcggct 2082

Seq_1 2161 atcaaaatccgaatcattggagccaggtaaatgcttatatctataaacatgatgggagcc 2220

||||||||||||||||||||||||||||||||||||||||||||||||||||||||||||

Seq_2 2083 atcaaaatccgaatcattggagccaggtaaatgcttatatctataaacatgatgggagcc 2142

Seq_1 2221 gagtaattgaattgaccggatcttggcctggaaaaccaatgactaaaaatgcagacggaa 2280

||||||||||||||||||||||||||||||||||||||||||||||||||||||||||||

Seq_2 2143 gagtaattgaattgaccggatcttggcctggaaaaccaatgactaaaaatgcagacggaa 2202

Seq_1 2281 tttacacgctgacgctgcctgcggacacggatacaaccaacgcaaaagtgatttttaata 2340

||||||||||||||||||||||||||||||||||||||||||||||||||||||||||||

Seq_2 2203 tttacacgctgacgctgcctgcggacacggatacaaccaacgcaaaagtgatttttaata 2262

Seq_1 2341 atggcagcgcccaagtgcccggtcagaatcagcctggctttgattacgtgctaaatggtt 2400

||||||||||||||||||||||||||||||||||||||||||||||||||||||||||||

Seq_2 2263 atggcagcgcccaagtgcccggtcagaatcagcctggctttgattacgtgctaaatggtt 2322

Seq_1 2401 tatataatgactcgggcttaagcggttctcttccccattgagggcaaggctagacgggac 2460

||||||||||||||||||||||||||||||||||||||||||||||||||||||||||||

Seq_2 2323 tatataatgactcgggcttaagcggttctcttccccattgagggcaaggctagacgggac 2382

Seq_1 2461 ttaccgaaagaaaccatcaatgatggtttcttttttgttcataaatcagacaaaactttt 2520

||||||||||||||||||||||||||||||||||||||||||||||||||||||||||||

Seq_2 2383 ttaccgaaagaaaccatcaatgatggtttcttttttgttcataaatcagacaaaactttt 2442

Seq_1 2521 ctcttgcaaaagtttgtgaagtgttgcacaatataaatgtgaaatacttcacaaacaaaa 2580

||||||||||||||||||||||||||||||||||||||||||||||||||||||||||||

Seq_2 2443 ctcttgcaaaagtttgtgaagtgttgcacaatataaatgtgaaatacttcacaaacaaaa 2502

Seq_1 2581 agacatcaaagagaaacataccctgcaaggatgctgatattgtctgcatttgcgccggag 2640

||||||||||||||||||||||||||||||||||||||||||||||||||||||||||||

Seq_2 2503 agacatcaaagagaaacataccctgcaaggatgctgatattgtctgcatttgcgccggag 2562

Seq_1 2641 caaaccaaaaacctggtgagacacgccttgaattagtagaaaagaacttgaagattttca 2700

||||||||||||||||||||||||||||||||||||||||||||||||||||||||||||

Seq_2 2563 caaaccaaaaacctggtgagacacgccttgaattagtagaaaagaacttgaagattttca 2622

Seq_1 2701 aaggcatcgttagtgaagtcatggcgagcggatttgacggcattttcttagtcgcgacgc 2760

||||||||||||||||||||||||||||||||||||||||||||||||||||||||||||

Seq_2 2623 aaggcatcgttagtgaagtcatggcgagcggatttgacggcattttcttagtcgcgacgc 2682

Seq_1 2761 gaggctggatggccttccccattatgattcttctcgcttccggcggcatcgggatgcccg 2820

||||||||||||||||||||||||||||||||||||||||||||||||||||||||||||

Seq_2 2683 gaggctggatggccttccccattatgattcttctcgcttccggcggcatcgggatgcccg 2742

Seq_1 2821 cgttgcaggccatgctgtccaggcaggtagatgacgaccatcagggacagcttcaaggat 2880

||||||||||||||||||||||||||||||||||||||||||||||||||||||||||||

Seq_2 2743 cgttgcaggccatgctgtccaggcaggtagatgacgaccatcagggacagcttcaaggat 2802

Seq_1 2881 cgctcgcggctcttaccagcctaacttcgatcactggaccgctgatcgtcacggcgattt 2940

||||||||||||||||||||||||||||||||||||||||||||||||||||||||||||

Seq_2 2803 cgctcgcggctcttaccagcctaacttcgatcactggaccgctgatcgtcacggcgattt 2862

Seq_1 2941 atgccgcctcggcgagcacatggaacgggttggcatggattgtaggcgccgccctatacc 3000

||||||||||||||||||||||||||||||||||||||||||||||||||||||||||||

Seq_2 2863 atgccgcctcggcgagcacatggaacgggttggcatggattgtaggcgccgccctatacc 2922

Seq_1 3001 ttgtctgcctccccgcgttgcgtcgcggtgcatggagccgggccacctactgaagtggat 3060

||||||||||||||||||||||||||||||||||||||||||||||||||||||||||||

Seq_2 2923 ttgtctgcctccccgcgttgcgtcgcggtgcatggagccgggccacctactgaagtggat 2982

Seq_1 3061 ttctttaagagctcctttaacttcctcaccagtagttgtatcggtaccataagtagaagc 3120

||||||||||||||||||||||||||||||||||||||||||||||||||||||||||||

Seq_2 2983 ttctttaagagctcctttaacttcctcaccagtagttgtatcggtaccataagtagaagc 3042

Seq_1 3121 agcaacccaagtagctttaccagcatccggttcaaccagcatagtaagaatcttactgga 3180

||||||||||||||||||||||||||||||||||||||||||||||||||||||||||||

Seq_2 3043 agcaacccaagtagctttaccagcatccggttcaaccagcatagtaagaatcttactgga 3102

Seq_1 3181 catcggcagttcttcgaacagtgcgccaactaccagctctttct-c-------------- 3225

||||||||||||||||||||||||||||||||||||||||||||#|##############

Seq_2 3103 catcggcagttcttcgaacagtgcgccaactaccagctctttctgcagttcattcagggc 3162

Seq_1 3226 ------------------------------------------------------------ 3225

############################################################

Seq_2 3163 accggagaacctgcgtgcaatccatcttgttcaatcatgcgaaacgatcctcatcctgtc 3222

Seq_1 3226 ------------------------------------------------------------ 3225

############################################################

Seq_2 3223 tcttgatccatggattacgcgttaacccgggcccgcggatgcatatgatcagatcttaag 3282

Seq_1 3226 ------------------------------------------------------------ 3225

############################################################

Seq_2 3283 gcctaggtctagaggatcgatctgtataataaagaataattattaatctgtagacaaatt 3342

Seq_1 3226 ------------------------------------------------------------ 3225

############################################################

Seq_2 3343 gtgaaaggatgtacttaaacgctaacggtcagctttattgaacagtaatttaagtatatg 3402

Seq_1 3226 ------------------------------------------------------------ 3225

############################################################

Seq_2 3403 tccaatctagggtaagtaaattgagtatcaatataaactttatatgaacataatcaacga 3462

Seq_1 3226 ------------------------------------------------------------ 3225

############################################################

Seq_2 3463 ggtgaaatcatgagcaatttgattaacggaaaaataccaaatcaagcgattcaaacatta 3522

Seq_1 3226 ------------------------------------------------------------ 3225

############################################################

Seq_2 3523 aaaatcgtaaaagatttatttggaagttcaatagttggagtatatctatttggttcagca 3582

Seq_1 3226 ------------------------------------------------------------ 3225

############################################################

Seq_2 3583 gtaaatggtggtttacgcattaacagcgatgtagatgttctagtcgtcgtgaatcatagt 3642

Seq_1 3226 ------------------------------------------------------------ 3225

############################################################

Seq_2 3643 ttacctcaattaactcgaaaaaaactaacagaaagactaatgactatatcaggaaagatt 3702

Seq_1 3226 ------------------------------------------------------------ 3225

############################################################

Seq_2 3703 ggaaatacggattctgttagaccacttgaagttacggttataaataggagtgaagttgtc 3762

Seq_1 3226 ------------------------------------------------------------ 3225

############################################################

Seq_2 3763 ccttggcaatatcctccaaaaagagaatttatatacggtgagtggctcaggggtgaattt 3822

Seq_1 3226 ------------------------------------------------------------ 3225

############################################################

Seq_2 3823 gagaatggacaaattcaggaaccaagctatgatcctgatttggctattgttttagcacaa 3882

Seq_1 3226 ------------------------------------------------------------ 3225

############################################################

Seq_2 3883 gcaagaaagaatagtatttctctatttggtcctgattcttcaagtatacttgtctccgta 3942

Seq_1 3226 ------------------------------------------------------------ 3225

############################################################

Seq_2 3943 cctttgacagatattcgaagagcaattaaggattctttgccagaactaattgaggggata 4002

Seq_1 3226 ------------------------------------------------------------ 3225

############################################################

Seq_2 4003 aaaggtgatgagcgtaatgtaattttaaccctagctcgaatgtggcaaacagtgactact 4062

Seq_1 3226 ----------------------------cagaatgggctatacctcttttacctaaagag 3257

############################||||||||||||||||||||||||||||||||

Seq_2 4063 ggtgaaattacctcgaaagatgtcgctgcagaatgggctatacctcttttacctaaagag 4122

Seq_1 3258 catgtaactttactggatatagctagaaaaggctatcggggagagtgtgatgataagtgg 3317

||||||||||||||||||||||||||||||||||||||||||||||||||||||||||||

Seq_2 4123 catgtaactttactggatatagctagaaaaggctatcggggagagtgtgatgataagtgg 4182

Seq_1 3318 gaaggactatattcaaaggtgaaagcactcgttaagtatatgaaaaattctatagaaact 3377

||||||||||||||||||||||||||||||||||||||||||||||||||||||||||||

Seq_2 4183 gaaggactatattcaaaggtgaaagcactcgttaagtatatgaaaaattctatagaaact 4242

Seq_1 3378 tctctcaattaggctaattttattgcaataacaggtgcttacttttctggagttctttag 3437

||||||||||||||||||||||||||||||||||||||||||||||||||||||||||||

Seq_2 4243 tctctcaattaggctaattttattgcaataacaggtgcttacttttctggagttctttag 4302

Seq_1 3438 caaatttttttattagctgaacttagtattagtggccatactcctccaatccaaagctat 3497

||||||||||||||||||||||||||||||||||||||||||||||||||||||||||||

Seq_2 4303 caaatttttttattagctgaacttagtattagtggccatactcctccaatccaaagctat 4362

Seq_1 3498 ttagaaagattactatatcctcaaacaggcggtaaccggcctcttcatcgggaatgcgcg 3557

||||||||||||||||||||||||||||||||||||||||||||||||||||||||||||

Seq_2 4363 ttagaaagattactatatcctcaaacaggcggtaaccggcctcttcatcgggaatgcgcg 4422

Seq_1 3558 cgaccttcagcatcaccggcatgtccccctggcggacgggaagtatccagctcgaggtcg 3617

||||||||||||||#|||||||||||||||||||||||||||||||||||||||||||||

Seq_2 4423 cgaccttcagcatcgccggcatgtccccctggcggacgggaagtatccagctcgaggtcg 4482

Seq_1 3618 ggccgcgttgctggcgtttttccataggctccgcccccctgacgagcatcacaaaaatcg 3677

||||||||||||||||||||||||||||||||||||||||||||||||||||||||||||

Seq_2 4483 ggccgcgttgctggcgtttttccataggctccgcccccctgacgagcatcacaaaaatcg 4542

Seq_1 3678 acgctcaagtcagaggtggcgaaacccgacaggactataaagataccaggcgtttccccc 3737

||||||||||||||||||||||||||||||||||||||||||||||||||||||||||||

Seq_2 4543 acgctcaagtcagaggtggcgaaacccgacaggactataaagataccaggcgtttccccc 4602

Seq_1 3738 tggaagctccctcgtgcgctctcctgttccgaccctgccgcttaccggatacctgtccgc 3797

||||||||||||||||||||||||||||||||||||||||||||||||||||||||||||

Seq_2 4603 tggaagctccctcgtgcgctctcctgttccgaccctgccgcttaccggatacctgtccgc 4662

Seq_1 3798 ctttctcccttcgggaagcgtggcgctttctcatagctcacgctgtaggtatctcagttc 3857

||||||||||||||||||||||||||||||||||||||||||||||||||||||||||||

Seq_2 4663 ctttctcccttcgggaagcgtggcgctttctcatagctcacgctgtaggtatctcagttc 4722

Seq_1 3858 ggtgtaggtcgttcgctccaagctgggctgtgtgcacgaaccccccgttcagcccgaccg 3917

||||||||||||||||||||||||||||||||||||||||||||||||||||||||||||

Seq_2 4723 ggtgtaggtcgttcgctccaagctgggctgtgtgcacgaaccccccgttcagcccgaccg 4782

Seq_1 3918 ctgcgccttatccggtaactatcgtcttgagtccaacccggtaagacacgacttatcgcc 3977

||||||||||||||||||||||||||||||||||||||||||||||||||||||||||||

Seq_2 4783 ctgcgccttatccggtaactatcgtcttgagtccaacccggtaagacacgacttatcgcc 4842

Seq_1 3978 actggcagcagccactggtaacaggattagcagagcgaggtatgtaggcggtgctacaga 4037

||||||||||||||||||||||||||||||||||||||||||||||||||||||||||||

Seq_2 4843 actggcagcagccactggtaacaggattagcagagcgaggtatgtaggcggtgctacaga 4902

Seq_1 4038 gttcttgaagtggtggcctaactacggctacactagaaggacagtatttggtatctgcgc 4097

||||||||||||||||||||||||||||||||||||||||||||||||||||||||||||

Seq_2 4903 gttcttgaagtggtggcctaactacggctacactagaaggacagtatttggtatctgcgc 4962

Seq_1 4098 tctgctgaagccagttaccttcggaaaaagagttgatagctcttgatccggcaaacaaac 4157

||||||||||||||||||||||||||||||||||||||||||||||||||||||||||||

Seq_2 4963 tctgctgaagccagttaccttcggaaaaagagttgatagctcttgatccggcaaacaaac 5022

Seq_1 4158 caccgctggtagcggtggtttttttgtttgcaagcagcagattacgcgcagaaaaaaagg 4217

||||||||||||||||||||||||||||||||||||||||||||||||||||||||||||

Seq_2 5023 caccgctggtagcggtggtttttttgtttgcaagcagcagattacgcgcagaaaaaaagg 5082

Seq_1 4218 atctcaagaagatcctttgatcttttctacggggtctgacgctcagtggaacgaaaactc 4277

||||||||||||||||||||||||||||||||||||||||||||||||||||||||||||

Seq_2 5083 atctcaagaagatcctttgatcttttctacggggtctgacgctcagtggaacgaaaactc 5142

Seq_1 4278 acgttaagggattttggtcatgagattatcaaaaaggatcttcacctagatccttttaaa 4337

||||||||||||||||||||||||||||||||||||||||||||||||||||||||||||

Seq_2 5143 acgttaagggattttggtcatgagattatcaaaaaggatcttcacctagatccttttaaa 5202

Seq_1 4338 ttaaaaatgaagttttaaatcaatctaaagtatatatgagtaaacttggtctgacagtta 4397

||||||||||||||||||||||||||||||||||||||||||||||||||||||||||||

Seq_2 5203 ttaaaaatgaagttttaaatcaatctaaagtatatatgagtaaacttggtctgacagtta 5262

Seq_1 4398 ccaatgcttaatcagtgaggcacctatctcagcgatctgtctatttcgttcatccatagt 4457

||||||||||||||||||||||||||||||||||||||||||||||||||||||||||||

Seq_2 5263 ccaatgcttaatcagtgaggcacctatctcagcgatctgtctatttcgttcatccatagt 5322

Seq_1 4458 tgcctgactccccgtcgtgtagataactacgatacgggagggcttaccatctggccccag 4517

||||||||||||||||||||||||||||||||||||||||||||||||||||||||||||

Seq_2 5323 tgcctgactccccgtcgtgtagataactacgatacgggagggcttaccatctggccccag 5382

Seq_1 4518 tgctgcaatgataccgcgagacccacgctcaccggctccagatttatcagcaataaacca 4577

||||||||||||||||||||||||||||||||||||||||||||||||||||||||||||

Seq_2 5383 tgctgcaatgataccgcgagacccacgctcaccggctccagatttatcagcaataaacca 5442

Seq_1 4578 gccagccggaagggccgagcgcagaagtggtcctgcaactttatccgcctccatccagtc 4637

||||||||||||||||||||||||||||||||||||||||||||||||||||||||||||

Seq_2 5443 gccagccggaagggccgagcgcagaagtggtcctgcaactttatccgcctccatccagtc 5502

Seq_1 4638 tattaattgttgccgggaagctagagtaagtagttcgccagttaatagtttgcgcaacgt 4697

||||||||||||||||||||||||||||||||||||||||||||||||||||||||||||

Seq_2 5503 tattaattgttgccgggaagctagagtaagtagttcgccagttaatagtttgcgcaacgt 5562

Seq_1 4698 tgttgccattgctgctggcatcgtggtgtcacgctcgtcgtttggtatggcttcattcag 4757

|||||||||||||||#||||||||||||||||||||||||||||||||||||||||||||

Seq_2 5563 tgttgccattgctgcaggcatcgtggtgtcacgctcgtcgtttggtatggcttcattcag 5622

Seq_1 4758 ctccggttcccaacgatcaaggcgagttacatgatcccccatgttgtgcaaaaaagcggt 4817

||||||||||||||||||||||||||||||||||||||||||||||||||||||||||||

Seq_2 5623 ctccggttcccaacgatcaaggcgagttacatgatcccccatgttgtgcaaaaaagcggt 5682

Seq_1 4818 tagctccttcggtcctccgatcgttgtcagaagtaagttggccgcagtgttatcactcat 4877

||||||||||||||||||||||||||||||||||||||||||||||||||||||||||||

Seq_2 5683 tagctccttcggtcctccgatcgttgtcagaagtaagttggccgcagtgttatcactcat 5742

Seq_1 4878 ggttatggcagcactgcataattctcttactgtcatgccatccgtaagatgcttttctgt 4937

||||||||||||||||||||||||||||||||||||||||||||||||||||||||||||

Seq_2 5743 ggttatggcagcactgcataattctcttactgtcatgccatccgtaagatgcttttctgt 5802

Seq_1 4938 gactggtgagtactcaaccaagtcattctgagaatagtgtatgcggcgaccgagttgctc 4997

||||||||||||||||||||||||||||||||||||||||||||||||||||||||||||

Seq_2 5803 gactggtgagtactcaaccaagtcattctgagaatagtgtatgcggcgaccgagttgctc 5862

Seq_1 4998 ttgcccggcgtcaacacgggataataccgcgccacatagcagaactttaaaagtgctcat 5057

||||||||||||||||||||||||||||||||||||||||||||||||||||||||||||

Seq_2 5863 ttgcccggcgtcaacacgggataataccgcgccacatagcagaactttaaaagtgctcat 5922

Seq_1 5058 cattggaaaacgttcttcggggcgaaaactctcaaggatcttaccgctgttgagatccag 5117

||||||||||||||||||||||||||||||||||||||||||||||||||||||||||||

Seq_2 5923 cattggaaaacgttcttcggggcgaaaactctcaaggatcttaccgctgttgagatccag 5982

Seq_1 5118 ttcgatgtaacccactcgtgcacccaactgatcttcagcatcttttactttcaccagcgt 5177

||||||||||||||||||||||||||||||||||||||||||||||||||||||||||||

Seq_2 5983 ttcgatgtaacccactcgtgcacccaactgatcttcagcatcttttactttcaccagcgt 6042

Seq_1 5178 ttctgggtgagcaaaaacaggaaggcaaaatgccgcaaaaaagggaataagggcgacacg 5237

||||||||||||||||||||||||||||||||||||||||||||||||||||||||||||

Seq_2 6043 ttctgggtgagcaaaaacaggaaggcaaaatgccgcaaaaaagggaataagggcgacacg 6102

Seq_1 5238 gaaatgttgaatactcatactcttcctttttcaatattattgaagcatttatcagggtta 5297

||||||||||||||||||||||||||||||||||||||||||||||||||||||||||||

Seq_2 6103 gaaatgttgaatactcatactcttcctttttcaatattattgaagcatttatcagggtta 6162

Seq_1 5298 ttgtctcatgagcggatacatatttgaatgtatttagaaaaataaacaaataggggttcc 5357

||||||||||||||||||||||||||||||||||||||||||||||||||||||||||||

Seq_2 6163 ttgtctcatgagcggatacatatttgaatgtatttagaaaaataaacaaataggggttcc 6222

Seq_1 5358 gcgcacatttccccgaaaagtgccacctgacgtctaagaaaccattattatcatgacatt 5417

||||||||||||||||||||||||||||||||||||||||||||||||||||||||||||

Seq_2 6223 gcgcacatttccccgaaaagtgccacctgacgtctaagaaaccattattatcatgacatt 6282

Seq_1 5418 aacctataaaaataggcgtatcacgaggccctttcgtcttcaagaattaacaaaattctc 5477

||||||||||||||||||||||||||||||||||||||||||||||||||||||||||||

Seq_2 6283 aacctataaaaataggcgtatcacgaggccctttcgtcttcaagaattaacaaaattctc 6342

Seq_1 5478 cagtcttcacatcggtttgaaaggaggaagcggaagaatgaagtaagagggatttttgac 5537

||||||||||||||||||||||||||||||||||||||||||||||||||||||||||||

Seq_2 6343 cagtcttcacatcggtttgaaaggaggaagcggaagaatgaagtaagagggatttttgac 6402

Seq_1 5538 tccgaagtaagtcttcaaaaaatcaaataaggagtgtcaagaatgtttgcaaaacgattc 5597

||||||||||||||||||||||||||||||||||||||||||||||||||||||||||||

Seq_2 6403 tccgaagtaagtcttcaaaaaatcaaataaggagtgtcaagaatgtttgcaaaacgattc 6462

Seq_1 5598 aaaacctctttactgccgttattcgctggatttttattgctgtttcatttggttctggca 5657

||||||||||||||||||||||||||||||||||||||||||||||||||||||||||||

Seq_2 6463 aaaacctctttactgccgttattcgctggatttttattgctgtttcatttggttctggca 6522

Seq_1 5658 ggaccggcggctgcgagtgctgaaacggcgaacaaatcgaatgagcttacagcaccgtcg 5717

||||||||||||||||||||||||||||||||||||||||||||||||||||||||||||

Seq_2 6523 ggaccggcggctgcgagtgctgaaacggcgaacaaatcgaatgagcttacagcaccgtcg 6582

Seq_1 5718 atcaaaagcggaaccattcttcatgcatggaattggtcgttcaatacgttaaaacacaat 5777

||||||||||||||||||||||||||||||||||||||||||||||||||||||||||||

Seq_2 6583 atcaaaagcggaaccattcttcatgcatggaattggtcgttcaatacgttaaaacacaat 6642

Seq_1 5778 atgaaggatattcatgatgcaggatatacagccattcagacatctccgattaaccaagta 5837

||||||||||||||||||||||||||||||||||||||||||||||||||||||||||||

Seq_2 6643 atgaaggatattcatgatgcaggatatacagccattcagacatctccgattaaccaagta 6702

Seq_1 5838 aaggaagggaatcaaggagataaaagcatgtcgaactggtactggctgtatcagccgaca 5897

||||||||||||||||||||||||||||||||||||||||||||||||||||||||||||

Seq_2 6703 aaggaagggaatcaaggagataaaagcatgtcgaactggtactggctgtatcagccgaca 6762

Seq_1 5898 tcgtatcaaattggcaaccgttacttaggtactgaacaagaatttaaagaaatgtgtgca 5957

||||||||||||||||||||||||||||||||||||||||||||||||||||||||||||

Seq_2 6763 tcgtatcaaattggcaaccgttacttaggtactgaacaagaatttaaagaaatgtgtgca 6822

Seq_1 5958 gccgctgaagaatatggcataaaggtcattgttgacgcggtcatcaatcataccaccagt 6017

||||||||||||||||||||||||||||||||||||||||||||||||||||||||||||

Seq_2 6823 gccgctgaagaatatggcataaaggtcattgttgacgcggtcatcaatcataccaccagt 6882

Seq_1 6018 gattatgccgcgatttccaatgaggttaagagtattccaaactggacacatggaaacaca 6077

||||||||||||||||||||||||||||||||||||||||||||||||||||||||||||

Seq_2 6883 gattatgccgcgatttccaatgaggttaagagtattccaaactggacacatggaaacaca 6942

Seq_1 6078 caaattaaaaactggtctgatcggatcctagaagcttatc 6117

||||||||||||||||||||||||||||||||||||||||

Seq_2 6943 caaattaaaaactggtctgatcggatcctagaagcttatc 6982

**Features [Seq_1]:**

**chloramphenicol resistance_cat_CDS : [392 : 1042 - CW]**

amy-back_amyE_CDS : [1413 : 2441 - CW]

ori_misc_feature : [3621 : 4235 - CW]

ampicillin resistance_bla_CDS : [5255 : 4395 - CCW]

amy-front_amyE_CDS : [5580 : 6100 - CW]

**Features [Seq_2]:**

**cat-check rev_misc_signal : [237 : 263 - CW]**

cat_CDS : [314 : 964 - CW]

¥amyE_CDS : [1335 : 2363 - CW]

spec_CDS : [3472 : 4254 - CW]

bla_CDS complement : [5260 : 6120 - CW]

amyE¥_CDS : [6445 : 6965 - CW]

amyE-check rev_misc_signal : [6843 : 6862 - CW]

## Alignment of Sequence_1: pBS4S with Sequence_2: pDG1731

Similarity : 4544/6673 (68,10 %)

Seq_1 1 gaattc-------------------------------gcggccgcttctagagcaatata 29

||||||##################################|##########|##|##|##

Seq_2 1 gaattcctgcagccctggcgaatggcgattttcg-ttcgtgaatacatgttataataact 59

Seq_1 30 tatactagtagcggcc-gctgcag------------------------------------ 52

###||||#||#||####|#||#######################################

Seq_2 60 ataactaataacgtaacg-tgactggcaagagatatttttaaaacaatgaataggtttac 118

Seq_1 53 ------------------------------------------------------------ 52

############################################################

Seq_2 119 acttactttagttttatggaaatgaaagatcatatcatatataatctagaataaaattaa 178

Seq_1 53 -------------------gataaaaaatttagaagccaatgaaatctataaataaacta 93

###################|||||||||||||||||||||||||||||||||||||||||

Seq_2 179 ctaaaataattattatctagataaaaaatttagaagccaatgaaatctataaataaacta 238

Seq_1 94 aattaagtttatttaattaacaactatggatataaaataggtactaatcaaaatagtgag 153

||||||||||||||||||||||||||||||||||||||||||||||||||||||||||||

Seq_2 239 aattaagtttatttaattaacaactatggatataaaataggtactaatcaaaatagtgag 298

Seq_1 154 gaggatatatttgaatacatacgaacaaattaataaagtgaaaaaaatacttcggaaaca 213

||||||||||||||||||||||||||||||||||||||||||||||||||||||||||||

Seq_2 299 gaggatatatttgaatacatacgaacaaattaataaagtgaaaaaaatacttcggaaaca 358

Seq_1 214 tttaaaaaataaccttattggtacttacatgtttggatcaggagttgagagtggactaaa 273

||||||||||||||||||||||||||||||||||||||||||||||||||||||||||||

Seq_2 359 tttaaaaaataaccttattggtacttacatgtttggatcaggagttgagagtggactaaa 418

Seq_1 274 accaaatagtgatcttgactttttagtcgtcgtatctgaaccattgacagatcaaagtaa 333

||||||||||||||||||||||||||||||||||||||||||||||||||||||||||||

Seq_2 419 accaaatagtgatcttgactttttagtcgtcgtatctgaaccattgacagatcaaagtaa 478

Seq_1 334 agaaatacttatacaaaaaattagacctatttcaaaaaaaataggagataaaagcaactt 393

||||||||||||||||||||||||||||||||||||||||||||||||||||||||||||

Seq_2 479 agaaatacttatacaaaaaattagacctatttcaaaaaaaataggagataaaagcaactt 538

Seq_1 394 acgatatattgaattaacaattattattcagcaagaaatggtaccgtggaatcatcctcc 453

||||||||||||||||||||||||||||||||||||||||||||||||||||||||||||

Seq_2 539 acgatatattgaattaacaattattattcagcaagaaatggtaccgtggaatcatcctcc 598

Seq_1 454 caaacaagaatttatttatggagaatggttacaagagctttatgaacaaggatacattcc 513

||||||||||||||||||||||||||||||||||||||||||||||||||||||||||||

Seq_2 599 caaacaagaatttatttatggagaatggttacaagagctttatgaacaaggatacattcc 658

Seq_1 514 tcagaaggaattaaattcagatttaaccataatgctttaccaagcaaaacgaaaaaataa 573

||||||||||||||||||||||||||||||||||||||||||||||||||||||||||||

Seq_2 659 tcagaaggaattaaattcagatttaaccataatgctttaccaagcaaaacgaaaaaataa 718

Seq_1 574 aagaatatacggaaattatgacttagaggaattactacctgatattccattttctgatgt 633

||||||||||||||||||||||||||||||||||||||||||||||||||||||||||||

Seq_2 719 aagaatatacggaaattatgacttagaggaattactacctgatattccattttctgatgt 778

Seq_1 634 gagaagagccattatggattcgtcagaggaattaatagataattatcaggatgatgaaac 693

||||||||||||||||||||||||||||||||||||||||||||||||||||||||||||

Seq_2 779 gagaagagccattatggattcgtcagaggaattaatagataattatcaggatgatgaaac 838

Seq_1 694 caactctatattaactttatgccgtatgattttaactatggacacgggtaaaatcatacc 753

||||||||||||||||||||||||||||||||||||||||||||||||||||||||||||

Seq_2 839 caactctatattaactttatgccgtatgattttaactatggacacgggtaaaatcatacc 898

Seq_1 754 aaaagatattgcgggaaatgcagtggctgaatcttctccattagaacatagggagagaat 813

||||||||||||||||||||||||||||||||||||||||||||||||||||||||||||

Seq_2 899 aaaagatattgcgggaaatgcagtggctgaatcttctccattagaacatagggagagaat 958

Seq_1 814 tttgttagcagttcgtagttatcttggagagaatattgaatggactaatgaaaatgtaaa 873

||||||||||||||||||||||||||||||||||||||||||||||||||||||||||||

Seq_2 959 tttgttagcagttcgtagttatcttggagagaatattgaatggactaatgaaaatgtaaa 1018

Seq_1 874 tttaactataaactatttaaataacagattaaaaaaattataaaaaaattgaaaaaatgg 933

||||||||||||||||||||||||||||||||||||||||||||||||||||||||||||

Seq_2 1019 tttaactataaactatttaaataacagattaaaaaaattataaaaaaattgaaaaaatgg 1078

Seq_1 934 tggaaacacttttttcaatttttttgttttattatttaatatttgggaaatattcattct 993

||||||||||||||||||||||||||||||||||||||||||||||||||||||||||||

Seq_2 1079 tggaaacacttttttcaatttttttgttttattatttaatatttgggaaatattcattct 1138

Seq_1 994 aattggtaatcagattttagaaaacaataaacccttgcatagggggatctcgaggccttc 1053

||||||||||||||||||||||||||||||||||||||||||||||||||||||||||||

Seq_2 1139 aattggtaatcagattttagaaaacaataaacccttgcatagggggatctcgaggccttc 1198

Seq_1 1054 cgaaaatgcgcggctttgaagctgagggtgcggcggcaatcgtgcgcaatgaagtgattg 1113

||||||||||||||||||||||||||||||||||||||||||||||||||||||||||||

Seq_2 1199 cgaaaatgcgcggctttgaagctgagggtgcggcggcaatcgtgcgcaatgaagtgattg 1258

Seq_1 1114 aaaatccggaaacaatagcgacagccattcgtatcggaaacccggcaagctgggacaaag 1173

||||||||||||||||||||||||||||||||||||||||||||||||||||||||||||

Seq_2 1259 aaaatccggaaacaatagcgacagccattcgtatcggaaacccggcaagctgggacaaag 1318

Seq_1 1174 ctgtaaaggcagccgaggaatccaatgggaaaattgacgaagtcactgatgatgaaatcc 1233

||||||||||||||||||||||||||||||||||||||||||||||||||||||||||||

Seq_2 1319 ctgtaaaggcagccgaggaatccaatgggaaaattgacgaagtcactgatgatgaaatcc 1378

Seq_1 1234 ttcacgcatatcagctgatcgcccgtgtagaaggcgtgtttgcagaaccaggttcttgcg 1293

||||||||||||||||||||||||||||||||||||||||||||||||||||||||||||

Seq_2 1379 ttcacgcatatcagctgatcgcccgtgtagaaggcgtgtttgcagaaccaggttcttgcg 1438

Seq_1 1294 cgtctatcgcaggagtgctgaaacaggtgaaatccggagaaattccgaaaggcagcaagg 1353

||||||||||||||||||||||||||||||||||||||||||||||||||||||||||||

Seq_2 1439 cgtctatcgcaggagtgctgaaacaggtgaaatccggagaaattccgaaaggcagcaagg 1498

Seq_1 1354 tcgtagctgtgttaacaggaaacggactgaaagatccgaacacagcggtcgacatttcag 1413

||||||||||||||||||||||||||||||||||||||||||||||||||||||||||||

Seq_2 1499 tcgtagctgtgttaacaggaaacggactgaaagatccgaacacagcggtcgacatttcag 1558

Seq_1 1414 aaatcaagcctgtcacattgccgactgatgaagacagcatccttgaatatgtaaaaggag 1473

||||||||||||||||||||||||||||||||||||||||||||||||||||||||||||

Seq_2 1559 aaatcaagcctgtcacattgccgactgatgaagacagcatccttgaatatgtaaaaggag 1618

Seq_1 1474 cggcccgtgtatgaacgaagccgacatgctgttctctgtcactgttcccggaagcacagc 1533

||||||||||||||||||||||||||||||||||||||||||||||||||||||||||||

Seq_2 1619 cggcccgtgtatgaacgaagccgacatgctgttctctgtcactgttcccggaagcacagc 1678

Seq_1 1534 taacctaggccccggctttgattcagtcggaatggcgctcagcagatatttgaagctgac 1593

||||||||||||||||||||||||||||||||||||||||||||||||||||||||||||

Seq_2 1679 taacctaggccccggctttgattcagtcggaatggcgctcagcagatatttgaagctgac 1738

Seq_1 1594 cgtctttgaaagcgacaaatggtcttttgaggctgaaacagaaacagtcgccggaattaa 1653

||||||||||||||||||||||||||||||||||||||||||||||||||||||||||||

Seq_2 1739 cgtctttgaaagcgacaaatggtcttttgaggctgaaacagaaacagtcgccggaattaa 1798

Seq_1 1654 ttcgagctcctttaacttcctcaccagtagttgtatcggtaccataagtagaagcagcaa 1713

||||||||||||||||||||||||||||||||||||||||||||||||||||||||||||

Seq_2 1799 ttcgagctcctttaacttcctcaccagtagttgtatcggtaccataagtagaagcagcaa 1858

Seq_1 1714 cccaagtagctttaccagcatccggttcaaccagcatagtaagaatcttactggacatcg 1773

||||||||||||||||||||||||||||||||||||||||||||||||||||||||||||

Seq_2 1859 cccaagtagctttaccagcatccggttcaaccagcatagtaagaatcttactggacatcg 1918

Seq_1 1774 gcagttcttcgaacagtgcgccaactaccagctctttctacagttcattcagggcaccgg 1833

|||||||||||||||||||||||||||||||||||||||#||||||||||||||||||||

Seq_2 1919 gcagttcttcgaacagtgcgccaactaccagctctttctgcagttcattcagggcaccgg 1978

Seq_1 1834 agaacctgcgtgcaatccatcttgttcaatcatgcgaaacgatcctcatcctgtctcttg 1893

||||||||||||||||||||||||||||||||||||||||||||||||||||||||||||

Seq_2 1979 agaacctgcgtgcaatccatcttgttcaatcatgcgaaacgatcctcatcctgtctcttg 2038

Seq_1 1894 atccatggattacgcg-------------------------------------------- 1909

||||||||||||||||############################################

Seq_2 2039 atccatggattacgcgttaacccgggcccgcggatgcatatgatcagatcctttaactct 2098

Seq_1 1910 ------------------------------------------------------------ 1909

############################################################

Seq_2 2099 ggcaaccctcaaaattgaatgagacatgctacacctccggataataaatatatataaacg 2158

Seq_1 1910 ------------------------------------------------------------ 1909

############################################################

Seq_2 2159 tatatagatttcataaagtctaacacactagacttatttacttcgtaattaagtcgttaa 2218

Seq_1 1910 ------------------------------------------------------------ 1909

############################################################

Seq_2 2219 accgtgtgctctacgaccaaaactataaaacctttaagaactttctttttttacaagaaa 2278

Seq_1 1910 ------------------------------------------------------------ 1909

############################################################

Seq_2 2279 aaagaaattagataaatctctcatatcttttattcaataatcgcatccgattgcagtata 2338

Seq_1 1910 ------------------------------------------------------------ 1909

############################################################

Seq_2 2339 aatttaacgatcactcatcatgttcatatttatcagagctcgtgctataattatactaat 2398

Seq_1 1910 ------------------------------------------------------------ 1909

############################################################

Seq_2 2399 tttataaggaggaaaaaatatgggcatttttagtatttttgtaatcagcacagttcatta 2458

Seq_1 1910 ------------------------------------------------------------ 1909

############################################################

Seq_2 2459 tcaaccaaacaaaaaataagtggttataatgaatcgttaataagcaaaattcatataacc 2518

Seq_1 1910 ------------------------------------------------------------ 1909

############################################################

Seq_2 2519 aaattaaagagggttataatgaacgagaaaaatataaaacacagtcaaaactttattact 2578

Seq_1 1910 ------------------------------------------------------------ 1909

############################################################

Seq_2 2579 tcaaaacataatatagataaaataatgacaaatataagattaaatgaacatgataatatc 2638

Seq_1 1910 ------------------------------------------------------------ 1909

############################################################

Seq_2 2639 tttgaaatcggctcaggaaaaggccattttacccttgaattagtaaagaggtgtaatttc 2698

Seq_1 1910 ------------------------------------------------------------ 1909

############################################################

Seq_2 2699 gtaactgccattgaaatagaccataaattatgcaaaactacagaaaataaacttgttgat 2758

Seq_1 1910 ------------------------------------------------------------ 1909

############################################################

Seq_2 2759 cacgataatttccaagttttaaacaaggatatattgcagtttaaatttcctaaaaaccaa 2818

Seq_1 1910 ------------------------------------------------------------ 1909

############################################################

Seq_2 2819 tcctataaaatatatggtaatataccttataacataagtacggatataatacgcaaaatt 2878

Seq_1 1910 ------------------------------------------------------------ 1909

############################################################

Seq_2 2879 gtttttgatagtatagctaatgagatttatttaatcgtggaatacgggtttgctaaaaga 2938

Seq_1 1910 ------------------------------------------------------------ 1909

############################################################

Seq_2 2939 ttattaaatacaaaacgctcattggcattacttttaatggcagaagttgatatttctata 2998

Seq_1 1910 ------------------------------------------------------------ 1909

############################################################

Seq_2 2999 ttaagtatggttccaagagaatattttcatcctaaacctaaagtgaatagctcacttatc 3058

Seq_1 1910 ------------------------------------------------------------ 1909

############################################################

Seq_2 3059 agattaagtagaaaaaaatcaagaatatcacacaaagataaacaaaagtataattatttc 3118

Seq_1 1910 ------------------------------------------------------------ 1909

############################################################

Seq_2 3119 gttatgaaatgggttaacaaagaatacaagaaaatatttacaaaaaatcaatttaacaat 3178

Seq_1 1910 ------------------------------------------------------------ 1909

############################################################

Seq_2 3179 tccttaaaacatgcaggaattgacgatttaaacaatattagctttgaacaattcttatct 3238

Seq_1 1910 ------------------------------------------------------------ 1909

############################################################

Seq_2 3239 cttttcaatagctataaattatttaataagtaagttaagggatgcataaactgcatccct 3298

Seq_1 1910 ------------------------------------------------------------ 1909

############################################################

Seq_2 3299 taacttgtttttcgtgtgcctattttttgtgaatcgattatgtcttttgcgcagtcggct 3358

Seq_1 1910 ------------------------------------------------------------ 1909

############################################################

Seq_2 3359 taaaccagttttcgctggtgcgaaaaaagagtgtcttgtgacacctaaattcaaaatcta 3418

Seq_1 1910 ------------------------------------------------------------ 1909

############################################################

Seq_2 3419 tcggtcagatttataccgatttgattttatatattcttgaataacatacgccgagttatc 3478

Seq_1 1910 ------------------------------------------------------------ 1909

############################################################

Seq_2 3479 acataaaagcgggaaccaatcatcaaatttaaacttcattgcataatccattaaactctt 3538

Seq_1 1910 ------------------------------------------------------------ 1909

############################################################

Seq_2 3539 aaattctacgattccttgttcatcaataaactcaatcatttctttaattaatttatatct 3598

Seq_1 1910 ------------------------------------------------------------ 1909

############################################################

Seq_2 3599 atctgttgttgttttctttaataattcatcaacatctacaccgccataaactatcatatc 3658

Seq_1 1910 ------------------------------------------------------------ 1909

############################################################

Seq_2 3659 ttctttttgatatttaaatttattaggatcttaaggcctaggtctagagtctttgttttg 3718

Seq_1 1910 ------------------------------------------------------------ 1909

############################################################

Seq_2 3719 acgccattagcgtacgtaacaatcctcgttaaaggacaaggacctgagcggaagtgtatc 3778

Seq_1 1910 ------------------------------------------------------------ 1909

############################################################

Seq_2 3779 gtacagtagacggagtatactagtatagtctatagtccgtggaattattatatttatctc 3838

Seq_1 1910 ------------------------------------------------------------ 1909

############################################################

Seq_2 3839 cgacgatattctcatcagtgaaatccagctggagttctttagcaaatttttttattagct 3898

Seq_1 1910 ------------------------------------------------------------ 1909

############################################################

Seq_2 3899 gaacttagtattagtggccatactcctccaatccaaagctatttagaaagattactatat 3958

Seq_1 1910 ------------------------------------------cgaccttcagcatcaccg 1927

##########################################||||||||||||||#|||

Seq_2 3959 cctcaaacaggcggtaaccggcctcttcatcgggaatgcgcgcgaccttcagcatcgccg 4018

Seq_1 1928 gcatgtccccctggcggacgggaagtatccagctcgaggtcgggccgcgttgctggcgtt 1987

||||||||||||||||||||||||||||||||||||||||||||||||||||||||||||

Seq_2 4019 gcatgtccccctggcggacgggaagtatccagctcgaggtcgggccgcgttgctggcgtt 4078

Seq_1 1988 tttccataggctccgcccccctgacgagcatcacaaaaatcgacgctcaagtcagaggtg 2047

||||||||||||||||||||||||||||||||||||||||||||||||||||||||||||

Seq_2 4079 tttccataggctccgcccccctgacgagcatcacaaaaatcgacgctcaagtcagaggtg 4138

Seq_1 2048 gcgaaacccgacaggactataaagataccaggcgtttccccctggaagctccctcgtgcg 2107

||||||||||||||||||||||||||||||||||||||||||||||||||||||||||||

Seq_2 4139 gcgaaacccgacaggactataaagataccaggcgtttccccctggaagctccctcgtgcg 4198

Seq_1 2108 ctctcctgttccgaccctgccgcttaccggatacctgtccgcctttctcccttcgggaag 2167

||||||||||||||||||||||||||||||||||||||||||||||||||||||||||||

Seq_2 4199 ctctcctgttccgaccctgccgcttaccggatacctgtccgcctttctcccttcgggaag 4258

Seq_1 2168 cgtggcgctttctcatagctcacgctgtaggtatctcagttcggtgtaggtcgttcgctc 2227

||||||||||||||||||||||||||||||||||||||||||||||||||||||||||||

Seq_2 4259 cgtggcgctttctcatagctcacgctgtaggtatctcagttcggtgtaggtcgttcgctc 4318

Seq_1 2228 caagctgggctgtgtgcacgaaccccccgttcagcccgaccgctgcgccttatccggtaa 2287

||||||||||||||||||||||||||||||||||||||||||||||||||||||||||||

Seq_2 4319 caagctgggctgtgtgcacgaaccccccgttcagcccgaccgctgcgccttatccggtaa 4378

Seq_1 2288 ctatcgtcttgagtccaacccggtaagacacgacttatcgccactggcagcagccactgg 2347

||||||||||||||||||||||||||||||||||||||||||||||||||||||||||||

Seq_2 4379 ctatcgtcttgagtccaacccggtaagacacgacttatcgccactggcagcagccactgg 4438

Seq_1 2348 taacaggattagcagagcgaggtatgtaggcggtgctacagagttcttgaagtggtggcc 2407

||||||||||||||||||||||||||||||||||||||||||||||||||||||||||||

Seq_2 4439 taacaggattagcagagcgaggtatgtaggcggtgctacagagttcttgaagtggtggcc 4498

Seq_1 2408 taactacggctacactagaaggacagtatttggtatctgcgctctgctgaagccagttac 2467

||||||||||||||||||||||||||||||||||||||||||||||||||||||||||||

Seq_2 4499 taactacggctacactagaaggacagtatttggtatctgcgctctgctgaagccagttac 4558

Seq_1 2468 cttcggaaaaagagttgatagctcttgatccggcaaacaaaccaccgctggtagcggtgg 2527

||||||||||||||||||||||||||||||||||||||||||||||||||||||||||||

Seq_2 4559 cttcggaaaaagagttgatagctcttgatccggcaaacaaaccaccgctggtagcggtgg 4618

Seq_1 2528 tttttttgtttgcaagcagcagattacgcgcagaaaaaaaggatctcaagaagatccttt 2587

||||||||||||||||||||||||||||||||||||||||||||||||||||||||||||

Seq_2 4619 tttttttgtttgcaagcagcagattacgcgcagaaaaaaaggatctcaagaagatccttt 4678

Seq_1 2588 gatcttttctacggggtctgacgctcagtggaacgaaaactcacgttaagggattttggt 2647

||||||||||||||||||||||||||||||||||||||||||||||||||||||||||||

Seq_2 4679 gatcttttctacggggtctgacgctcagtggaacgaaaactcacgttaagggattttggt 4738

Seq_1 2648 catgagattatcaaaaaggatcttcacctagatccttttaaattaaaaatgaagttttaa 2707

||||||||||||||||||||||||||||||||||||||||||||||||||||||||||||

Seq_2 4739 catgagattatcaaaaaggatcttcacctagatccttttaaattaaaaatgaagttttaa 4798

Seq_1 2708 atcaatctaaagtatatatgagtaaacttggtctgacagttaccaatgcttaatcagtga 2767

||||||||||||||||||||||||||||||||||||||||||||||||||||||||||||

Seq_2 4799 atcaatctaaagtatatatgagtaaacttggtctgacagttaccaatgcttaatcagtga 4858

Seq_1 2768 ggcacctatctcagcgatctgtctatttcgttcatccatagttgcctgactccccgtcgt 2827

||||||||||||||||||||||||||||||||||||||||||||||||||||||||||||

Seq_2 4859 ggcacctatctcagcgatctgtctatttcgttcatccatagttgcctgactccccgtcgt 4918

Seq_1 2828 gtagataactacgatacgggagggcttaccatctggccccagtgctgcaatgataccgcg 2887

||||||||||||||||||||||||||||||||||||||||||||||||||||||||||||

Seq_2 4919 gtagataactacgatacgggagggcttaccatctggccccagtgctgcaatgataccgcg 4978

Seq_1 2888 agacccacgctcaccggctccagatttatcagcaataaaccagccagccggaagggccga 2947

||||||||||||||||||||||||||||||||||||||||||||||||||||||||||||

Seq_2 4979 agacccacgctcaccggctccagatttatcagcaataaaccagccagccggaagggccga 5038

Seq_1 2948 gcgcagaagtggtcctgcaactttatccgcctccatccagtctattaattgttgccggga 3007

||||||||||||||||||||||||||||||||||||||||||||||||||||||||||||

Seq_2 5039 gcgcagaagtggtcctgcaactttatccgcctccatccagtctattaattgttgccggga 5098

Seq_1 3008 agctagagtaagtagttcgccagttaatagtttgcgcaacgttgttgccattgctgctgg 3067

|||||||||||||||||||||||||||||||||||||||||||||||||||||||||#||

Seq_2 5099 agctagagtaagtagttcgccagttaatagtttgcgcaacgttgttgccattgctgcagg 5158

Seq_1 3068 catcgtggtgtcacgctcgtcgtttggtatggcttcattcagctccggttcccaacgatc 3127

||||||||||||||||||||||||||||||||||||||||||||||||||||||||||||

Seq_2 5159 catcgtggtgtcacgctcgtcgtttggtatggcttcattcagctccggttcccaacgatc 5218

Seq_1 3128 aaggcgagttacatgatcccccatgttgtgcaaaaaagcggttagctccttcggtcctcc 3187

||||||||||||||||||||||||||||||||||||||||||||||||||||||||||||

Seq_2 5219 aaggcgagttacatgatcccccatgttgtgcaaaaaagcggttagctccttcggtcctcc 5278

Seq_1 3188 gatcgttgtcagaagtaagttggccgcagtgttatcactcatggttatggcagcactgca 3247

||||||||||||||||||||||||||||||||||||||||||||||||||||||||||||

Seq_2 5279 gatcgttgtcagaagtaagttggccgcagtgttatcactcatggttatggcagcactgca 5338

Seq_1 3248 taattctcttactgtcatgccatccgtaagatgcttttctgtgactggtgagtactcaac 3307

||||||||||||||||||||||||||||||||||||||||||||||||||||||||||||

Seq_2 5339 taattctcttactgtcatgccatccgtaagatgcttttctgtgactggtgagtactcaac 5398

Seq_1 3308 caagtcattctgagaatagtgtatgcggcgaccgagttgctcttgcccggcgtcaacacg 3367

||||||||||||||||||||||||||||||||||||||||||||||||||||||||||||

Seq_2 5399 caagtcattctgagaatagtgtatgcggcgaccgagttgctcttgcccggcgtcaacacg 5458

Seq_1 3368 ggataataccgcgccacatagcagaactttaaaagtgctcatcattggaaaacgttcttc 3427

||||||||||||||||||||||||||||||||||||||||||||||||||||||||||||

Seq_2 5459 ggataataccgcgccacatagcagaactttaaaagtgctcatcattggaaaacgttcttc 5518

Seq_1 3428 ggggcgaaaactctcaaggatcttaccgctgttgagatccagttcgatgtaacccactcg 3487

||||||||||||||||||||||||||||||||||||||||||||||||||||||||||||

Seq_2 5519 ggggcgaaaactctcaaggatcttaccgctgttgagatccagttcgatgtaacccactcg 5578

Seq_1 3488 tgcacccaactgatcttcagcatcttttactttcaccagcgtttctgggtgagcaaaaac 3547

||||||||||||||||||||||||||||||||||||||||||||||||||||||||||||

Seq_2 5579 tgcacccaactgatcttcagcatcttttactttcaccagcgtttctgggtgagcaaaaac 5638

Seq_1 3548 aggaaggcaaaatgccgcaaaaaagggaataagggcgacacggaaatgttgaatactcat 3607

||||||||||||||||||||||||||||||||||||||||||||||||||||||||||||

Seq_2 5639 aggaaggcaaaatgccgcaaaaaagggaataagggcgacacggaaatgttgaatactcat 5698

Seq_1 3608 actcttcctttttcaatattattgaagcatttatcagggttattgtctcatgagcggata 3667

||||||||||||||||||||||||||||||||||||||||||||||||||||||||||||

Seq_2 5699 actcttcctttttcaatattattgaagcatttatcagggttattgtctcatgagcggata 5758

Seq_1 3668 catatttgaatgtatttagaaaaataaacaaataggggttccgcgcacatttccccgaaa 3727

||||||||||||||||||||||||||||||||||||||||||||||||||||||||||||

Seq_2 5759 catatttgaatgtatttagaaaaataaacaaataggggttccgcgcacatttccccgaaa 5818

Seq_1 3728 agtgccacctgacgtctaagaaaccattattatcatgacattaacctataaaaataggcg 3787

||||||||||||||||||||||||||||||||||||||||||||||||||||||||||||

Seq_2 5819 agtgccacctgacgtctaagaaaccattattatcatgacattaacctataaaaataggcg 5878

Seq_1 3788 tatcacgaggccctttcgtcttcaagaattaattcatgtaaaagatgaggttggttcatt 3847

||||||||||||||||||||||||||||||||||||||||||||||||||||||||||||

Seq_2 5879 tatcacgaggccctttcgtcttcaagaattaattcatgtaaaagatgaggttggttcatt 5938

Seq_1 3848 ctcgaaaattacatctgtgttctcagagcggggcgtgacgtttgaaaaaatccttcagct 3907

||||||||||||||||||||||||||||||||||||||||||||||||||||||||||||

Seq_2 5939 ctcgaaaattacatctgtgttctcagagcggggcgtgacgtttgaaaaaatccttcagct 5998

Seq_1 3908 gccaattaaaggccatgatgagttagctgaaatcgtaattgtcacacatcatacatcaga 3967

||||||||||||||||||||||||||||||||||||||||||||||||||||||||||||

Seq_2 5999 gccaattaaaggccatgatgagttagctgaaatcgtaattgtcacacatcatacatcaga 6058

Seq_1 3968 agctgatttcagtgatatcctgcaaaacctaaatgatttggaagtcgttcaagaagtcaa 4027

||||||||||||||||||||||||||||||||||||||||||||||||||||||||||||

Seq_2 6059 agctgatttcagtgatatcctgcaaaacctaaatgatttggaagtcgttcaagaagtcaa 6118

Seq_1 4028 aagcacatatcgtgtagaagggaacggttggagctaatgtggaaaggacttatccatcaa 4087

||||||||||||||||||||||||||||||||||||||||||||||||||||||||||||

Seq_2 6119 aagcacatatcgtgtagaagggaacggttggagctaatgtggaaaggacttatccatcaa 6178

Seq_1 4088 tataaagaatttttacctgtaacagatcaaacaccggcgctaactttacatgaaggaaac 4147

||||||||||||||||||||||||||||||||||||||||||||||||||||||||||||

Seq_2 6179 tataaagaatttttacctgtaacagatcaaacaccggcgctaactttacatgaaggaaac 6238

Seq_1 4148 acacctcttattcacctgccgaagctgtctgagcagctcggaattgagcttcatgtcaaa 4207

||||||||||||||||||||||||||||||||||||||||||||||||||||||||||||

Seq_2 6239 acacctcttattcacctgccgaagctgtctgagcagctcggaattgagcttcatgtcaaa 6298

Seq_1 4208 acggaaggcgtcaatcctacgggatcatttaaagatcgcggaatggttatggctgtggca 4267

||||||||||||||||||||||||||||||||||||||||||||||||||||||||||||

Seq_2 6299 acggaaggcgtcaatcctacgggatcatttaaagatcgcggaatggttatggctgtggca 6358

Seq_1 4268 aaggcaaaagaagaaggcaatgacacgattatgtgcgcgtcaacaggtaacacttccgct 4327

||||||||||||||||||||||||||||||||||||||||||||||||||||||||||||

Seq_2 6359 aaggcaaaagaagaaggcaatgacacgattatgtgcgcgtcaacaggtaacacttccgct 6418

Seq_1 4328 gctgcggcagcatatgcagcccgtgctaacatgaaatgcattgtcatcatcccgaacgga 4387

||||||||||||||||||||||||||||||||||||||||||||||||||||||||||||

Seq_2 6419 gctgcggcagcatatgcagcccgtgctaacatgaaatgcattgtcatcatcccgaacgga 6478

Seq_1 4388 aaaattgcatttggaaaactcgctcaagctgtcatgtacggagccgagattatcgcaatt 4447

||||||||||||||||||||||||||||||||||||||||||||||||||||||||||||

Seq_2 6479 aaaattgcatttggaaaactcgctcaagctgtcatgtacggagccgagattatcgcaatt 6538

Seq_1 4448 gacggaaactttgacgatgcgcttaaaattgtccgttccatctgtgagaaatcaccgatt 4507

||||||||||||||||||||||||||||||||||||||||||||||||||||||||||||

Seq_2 6539 gacggaaactttgacgatgcgcttaaaattgtccgttccatctgtgagaaatcaccgatt 6598

Seq_1 4508 gcccttgtcaactcagtcaacccttaccgcattgaaggccaaaaaactgctgccttcgga 4567

||||||||||||||||||||||||||||||||||||||||||||||||||||||||||||

Seq_2 6599 gcccttgtcaactcagtcaacccttaccgcattgaaggccaaaaaactgctgccttcgga 6658

Seq_1 4568 tcctagaagcttatc 4582

|||||||||||||||

Seq_2 6659 tcctagaagcttatc 6673

**Features [Seq_1]:**

**spc_CDS : [242 : 916 - CW]**

'thrC_CDS : [1059 : 1487 - CW]

thrB_CDS : [1484 : 1894 - CW]

mutation : [ : - CW]

ori approx._CDS : [1973 : 2587 - CW]

bla_CDS : [3607 : 2747 - CCW]

mutation : [ : - CW]

hom_CDS : [3762 : 4064 - CW]

thrC'_CDS : [4064 : 4573 - CW]

**Features [Seq_2]:**

**spc_CDS : [387 : 1061 - CW]**

'thrC_CDS : [1204 : 1632 - CW]

thrB_CDS : [1629 : 2039 - CW]

erm_CDS : [2537 : 3271 - CW]

bla_CDS complement : [4838 : 5698 - CW]

hom_CDS : [5853 : 6155 - CW]

thrC'_CDS : [6155 : 6664 - CW]
