# Supplementary material for: The Bacillus BioBrick Box: generation and evaluation of essential genetic building blocks for standardized work with Bacillus subtilis
Source: J Biol Eng. 2013 Dec 2;7:29. doi: 10.1186/1754-1611-7-29 (PMC4177231; doi:10.1186/1754-1611-7-29)
Supplement: Additional file 3 — Supplemental Figures, Tables and Text. [file 1754-1611-7-29-S3.docx]

Supplementary Information to

**Radeck *et al*. “The *Bacillus* BioBrick Box: Generation and Evaluation of Essential Genetic Building Blocks for Standardized Work with *Bacillus subtilis***

Additional file 3 [.docx]: Supplemental Figures, Tables and Text.

Contents

[Table S1. Plasmids used in this study 2](#_Toc372548831)

[Table S2. Bacterial strains used in this study 3](#_Toc372548832)

[Table S3. Primers used in this study 4](#_Toc372548833)

[Figure S1. Expression of P*_hom_*-*luxABCDE* during growth in different media. 5](#_Toc372548834)

[Figure S2. Correlation between reporter output of lacZ and lux. 6](#_Toc372548835)

[Figure S3. Determination of luminescence half-life. 7](#_Toc372548836)

[Figure S4: Effects of different carbon sources on xylose-dependent induction of P*_xylA_*. 8](#_Toc372548837)

[Protocols 9](#_Toc372548838)

[Luria-Bertani (LB) broth: 9](#_Toc372548839)

[Starch plates: 9](#_Toc372548840)

[Chemical defined medium (CSE): (100ml) 9](#_Toc372548841)

[MOPS-based chemically defined medium (MCSE) (100ml) 10](#_Toc372548842)

[Antibiotics 11](#_Toc372548843)

[QuikChange Site Directed Mutagenesis 11](#_Toc372548844)

[Plasmid Extraction from *E. coli*  - Alkaline Lysis Method 13](#_Toc372548845)

[Transformation of *Bacillus subtilis* (simple) 14](#_Toc372548846)

[Competent *E. coli* cells 15](#_Toc372548847)

[β-Galactosidase Assay for *B. subtilis* (based on Miller, 1972) 19](#_Toc372548848)

[Western blot detection of GFP 21](#_Toc372548849)

[Detection of Flag-tag on Western blots 22](#_Toc372548850)

[Detection of His-tag on Western Blots 23](#_Toc372548851)

[Detect strep-tag on Western blots with Strep-Tactin-HRP conjugate (IBA) 24](#_Toc372548852)

[Detect HA-tag on Western blots 25](#_Toc372548853)

[Detection of cMyc on Western blots 26](#_Toc372548854)

[How to work with *Bacillus subtilis* vectors 27](#_Toc372548855)

[Pre-Cloning in *E. coli* 27](#_Toc372548856)

[Linearisation before transformation in *B. subtilis* 28](#_Toc372548857)

[Verification of correct integration 28](#_Toc372548858)

## Table S1. Plasmids used in this study

| **Name** | **Description*^a^*** | **Source** |
| --- | --- | --- |
| Plasmids |  |  |
| pAC6 | Vector for transcriptional promoter fusions to *lacZ*; integrates at *amyE*; cm^r^ | [[25](#_ENREF_25)] |
| pAH328 | Vector for transcriptional promoter fusions to *luxABCDE* (luciferase); integrates at *sacA*; cm^r^ | [[26](#_ENREF_26)] |
| pDG1662 | Empty vector, integrates at *amyE*, cm^r^, spc^r^, amp^r^ | [[23](#_ENREF_23)] |
| pDG1731 | Empty vector; integrates at *thrC*, spc^r^, mls^r^, amp^r^ | [[23](#_ENREF_23)] |
| pAX01 | Vector for xylose-dependent gene expression; integrates at *lacA*, mls^r^, amp^r^ | [[24](#_ENREF_24)] |
| pXT | Vector for xylose-inducible gene expression; integrates in *thrC*; spc^r^, amp^r^ | [[46](#_ENREF_46)] |
| pSB1C3 | Replicative *E. coli* vector, MCS features *rfp*-cassette; cm^r^ | [[62](#_ENREF_62)] |
| pGFPamy | Vector for transcriptional promoter fusions to *gfpmut3*; integrates at *amyE*; cm^r^, amp^r^ | [[63](#_ENREF_63)] |
| pBS1C | Empty vector, integrates at *amyE*; cm^r^ | This study |
| pBS2E | Empty vector, integrates at *lacA*; mls^r^ | This study |
| pBS4S | Empty vector, integrates at *thrC*; spc^r^ | This study |
| pBS1C*lacZ* | Vector for transcriptional promoter fusions to *lacZ*; integrates at *amyE*; cm^r^ | This study |
| pBS1C*lacZ-0* | pBS1C*lacZ* without promoter | This study |
| pBS1C*lacZ*-P*_liaI_* | pBS1C*lacZ*-P*_liaI_-lacZ* | This study |
| pBS3C*lux* | Vector for transcriptional promoter fusions to *luxABCDE* (luciferase); integrates in *sacA*; cm^r^ | This study |
| pBS3C*lux-0* | pBS3C*lux* without promoter | This study |
| pBS3C*lux*-J23101 | pBS3C*lux*-J23101-*luxABCDE* | This study |
| pBS3C*lux*-P*_liaG_* | pBS3C*lux*-P*_liaG_*-*luxABCDE* | This study |
| pBS3C*lux*-P*_lepA_* | pBS3C*lux*-P*_lepA_*-*luxABCDE* | This study |
| pBS3C*lux*-P*_veg_* | pBS3C*lux*-P*_veg_*-*luxABCDE* | This study |
| pBS3C*lux*-P*_liaI_* | pBS3C*lux*-P*_liaI_*-*luxABCDE* | This study |
| pBS3C*lux*-P*_xylA_* | pBS3C*lux*-P*_xylA_*-*luxABCDE* | This study |
| pBS0K*Pspac** | Replicative expression vector with constitutive P*_spac_*; pDG148 derivative | This study, [[69](#_ENREF_69)] |
| pBS0K*Pspac**-Flag-*gfp* | pBS0K*Pspac**-Flag-*gfp* | This study |
| pBS0K*Pspac**-*gfp*-Flag | pBS0K*Pspac**-*gfp*-Flag | This study |
| pBS0K*Pspac**-HA-*gfp* | pBS0K*Pspac**-HA-*gfp* | This study |
| pBS0K*Pspac**-*gfp*-HA | pBS0K*Pspac**-*gfp*-HA | This study |
| pBS0K*Pspac**-cMyc-*gfp* | pBS0K*Pspac**-cMyc-*gfp* | This study |
| pBS0K*Pspac**-*gfp*-cMyc | pBS0K*Pspac**-*gfp*-cMyc | This study |
| pBS0K*Pspac**-His-*gfp* | pBS0K*Pspac**-His-*gfp* | This study |
| pBS0K*Pspac**-*gfp*-His | pBS0K*Pspac**-*gfp*-His | This study |
| pBS0K*Pspac**-StrepII-*gfp* | pBS0K*Pspac**-StrepII-*gfp* | This study |
| pBS0K*Pspac**-*gfp*-StrepII | pBS0K*Pspac**-*gfp*-StrepII | This study |
| pBS0K*Pspac**-Flag-*gfp* | pBS0K*Pspac**-Flag-*gfp* | This study |
| pCSlux101 | pAH328-P*_hom_*-*luxABCDE*; promoter fragment amplified with primers TM2377+2474 | This study |

cm^r^, chloramphenicol resistance; kan^r^, kanamycin resistance; spc^r^, spectinomycin resistance; mls^r^, erythromycin-induced resistance to macrolide, lincosamide and streptogramin B antibiotics (MLS); 0: no insert, but *rfp*-cassette was removed by cleavage with XbaI and SpeI and religation

## Table S2. Bacterial strains used in this study

| **Name** | **Description*^a^*** | **Source** |
| --- | --- | --- |
| *E. coli* strains | | |
| XL1-Blue | *recA1 endA1 gyrA96 thi-1 hsdR17 supE44 relA1 lac F′::*Tn*10*  *proAB lacI^q^* Δ(*lacZ*)M15] | Stratagene |
| *B. subtilis* strains | | |
| W168 | Wild-type, *trpC2* | Laboratory stock |
| TMB1872 | W168 *sacA*::pBS3C*lux-0* | This study |
| TMB1862 | W168 *sacA*:: pBS3C*lux*-J23101-*luxABCDE* | This study |
| TMB1856 | W168 *sacA*:: pBS3C*lux*-P*_liaG_*-*luxABCDE* | This study |
| TMB1860 | W168 *sacA*:: pBS3C*lux*-P*_lepA_*-*luxABCDE* | This study |
| TMB1930 | W168 *sacA*:: pBS3C*lux*-P*_veg_*-*luxABCDE* | This study |
| TMB1858 | W168 *sacA*:: pBS3C*lux*-P*_liaI_*-*luxABCDE* | This study |
| TMB1931 | W168 *sacA*:: pBS3C*lux*-P*_xylA_*-*luxABCDE* | This study |
| TMB1939 | W168 *amyE*::pBS1ClacZ-0 | This study |
| TMB1857 | W168 *amyE*::pBS1ClacZ-P*_liaI_-lacZ* | This study |
| TMB1920 | W168 pBS0K*Pspac**-Flag-*gfp* | This study |
| TMB1921 | W168 pBS0K*Pspac**-*gfp*-Flag | This study |
| TMB1922 | W168 pBS0K*Pspac**-HA-*gfp* | This study |
| TMB1923 | W168 pBS0K*Pspac**-*gfp*-HA | This study |
| TMB1924 | W168 pBS0K*Pspac**-cMyc-*gfp* | This study |
| TMB1925 | W168 pBS0K*Pspac**-*gfp*-cMyc | This study |
| TMB1926 | W168 pBS0K*Pspac**-His-*gfp* | This study |
| TMB1927 | W168 pBS0K*Pspac**-*gfp*-His | This study |
| TMB1928 | W168 pBS0K*Pspac**-StrepII-*gfp* | This study |
| TMB1929 | W168 pBS0K*Pspac**-*gfp*-StrepII | This study |
| SGB171 | W168 *sacA*::pCSlux101 | This study |

0: no insert, but *rfp*-cassette was removed by cleavage with XbaI and SpeI and religation

## Table S3. Primers used in this study

| **Primer name** | **Sequence (5'-3')** |
| --- | --- |
| Oligonucleotides for cloning vectors ***^a^*** | |
| TM2206 | CGTTGTTGCCATTG**CTGCCG**GCATCGTGGTGTC |
| TM2207 | gacaccacgatgc**cggcag**caatggcaacaacg |
| TM2845 | GTGCGCCAACTACCAGCTCTTT**CTCCAG**AATGGGCTATACCTC |
| TM2846 | GAGGTATAGCCCATT**CTGGAG**AAAGAGCTGGTAGTTGGCGCAC |
| TM2843 | TTTCGCTAAGGATGATTTCTGG |
| TM2844 | GATC**GGTCTC**GAATTGACACCTTGCCCTTTTTTGCC |
| TM2975 | GATC**GGTCTC**CCTAGGACTCTCTAGCTTGAGGCATC |
| TM2976 | GATC**GGTCTC**CCTAGGAGTTAACAAGAGTTTGTAGA |
| TM2608 | AAATT**ATGCAT**CTTTCGCTAAGGATGATTTCTGG |
| TM2609 | GACACCTTGCCCTTTTTTGCC |
| TM2835 | CCAACTACCAGCTCTTT**CTACAG**TTCATTCAGGGC |
| TM2836 | GCCCTGAATGAA**CTGTAG**AAAGAGCTGGTAGTTGG |
| TM2837 | GTAC**CTGCAG**GATAAAAAATTTAGAAGCCAATG |
| TM2838 | TTAGTCCACTCTCAACTCC |
| TM2301 | AATTCGCGGCCGCTTCTAGATGGCCGGCACCGGTTAATACTAGTAGCGGCCGCTGCAGG |
| TM2302 | GATCCCTGCAGCGGCCGCTACTAGTATTAACCGGTGCCGGCCATCTAGAAGCGGCCGCG |
| TM2885 | GCGTTTGATAGTTGATATC**CAGCAG**GATCCTGAGCG |
| TM2886 | CGCTCAGGATC**CTGCTG**GATATCAACTATCAAACGC |
| TM2887 | CCCATTAATGAATTGCCGGATAA**TCTTGA**TTTTGAAGGCC |
| TM2888 | GGCCTTCAAAA**TCAAGA**TTATCCGGCAATTCATTAATGGG |
| TM2884 | GATC**GGTCTC**GCTAGGACACCTTGCCCTTTTTTGCC |
| TM3005 | GCGACCTTCAGCATC**ACCGGC**ATGTCCCCCTGGC |
| TM3006 | GCCAGGGGGACAT**GCCGGT**GATGCTGAAGGTCGC |
| TM3011 | ACGTTGTTGCCATTGCT**GCTGGC**ATCGTGGTGTC |
| TM3012 | GACACCACGAT**GCCAGC**AGCAATGGCAACAACGT |
| TM3013 | GCCGGACGCATCGTG**GCAGGC**ATCACCGGCG |
| TM3014 | CGCCGGTGAT**GCCTGCC**ACGATGCGTCCGGC |
| TM3028 | CCTCGACCTGAATGGAA**GCTGGC**GGCACCTCGCTAACGG |
| TM3209 | CCGTTAGCGAGGTGCC**GCCAGC**TTCCATTCAGGTCGAGG |
| Oligonucleotides for promoters ***^b^*** | |
| TM2891 | GATC**GAATTC**GCGGCCGCT**TCTAGA**GCAAAAATCAGACCAGACAAAAGC |
| TM2892 | GATC**ACTAGT**ATCATTCATTCTATTATAAAGGAAAAGC |
| TM2895 | GATC**GAATTC**GCGGCCGCT**TCTAGA**GATTGGCCAAAGCAGAAAGGTCC |
| TM2896 | GATC**ACTAGT**ATCGTTTTCCTTGTCTTCATCTTATAC |
| TM2899 | GATC**GAATTC**GCGGCCGCT**TCTAGA**GAGTCAATGTATGAATGGATACG |
| TM2890 | GATC**ACTAGT**AACTATTAAACGCAAAATACACTAG |
| TM2903 | GATC**GAATTC**GCGGCCGCT**TCTAGAG**GGAGTTCTGAGAATTGGTATGC |
| TM2904 | GATC**ACTAGT**AACTACATTTATTGTACAACACGAGC |
| TM2968 | GATC**GAATTC**GCGGCCGCT**TCTAGAG**AAGGCCAAAAAACTGCTGCC |
| TM2969 | GATC**ACTAGT**ATTCGATAAGCTTGGGATCCC |
| TM2934 | GATC**GAATTC**GCGGCCGCT**TCTAGA**TAAGGAGGAACTACTATG**GCCGGC**AGTAAAGGAGAAGAACTTTTC |
| TM2935 | GATC**ACTAGT**ATTA**ACCGGT**TTTGTAGAGCTCATCCATGC |
| TM2377 | AATT**GTCGAC**ATAAGCTTATCCTGATGGTC |
| TM2474 | AATT**GAGCTC**AGGGCTTTCTCTTTTTACAG |

***^a^*** Recognition sites for endonuclease restriction enzymes are in bold, resulting overhangs underlined. Single nucleotides in bold and underlined are introduced mutations at restriction sites.

***^b^*** Introduced restriction sites in the overhang shown in bold, annealing part is underlined.


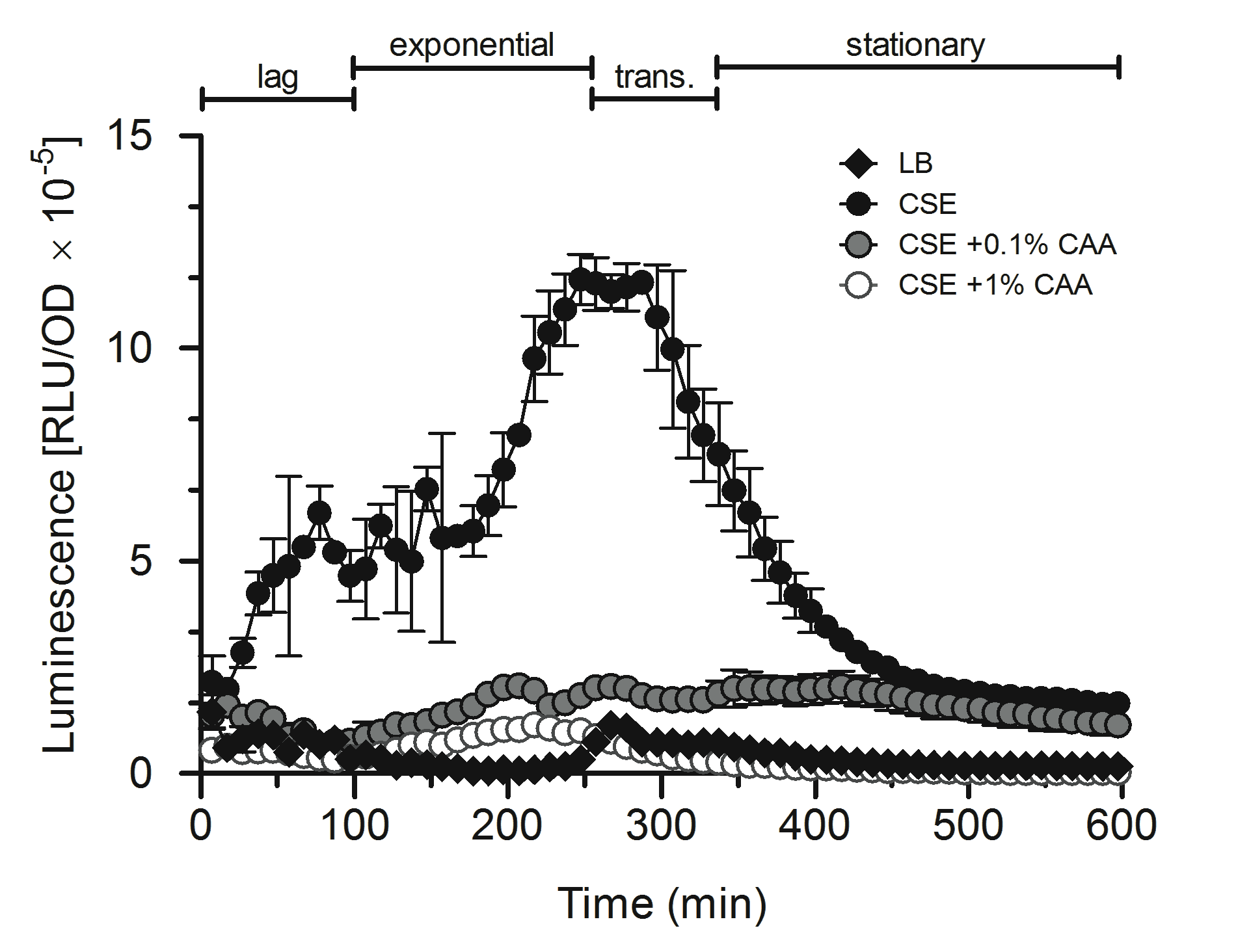


Figure S1. Expression of P*_hom_*-*luxABCDE* during growth in different media.

Wild-type *B. subtilis* carrying the P*_hom_*-*luxABCDE* reporter construct was grown in LB medium, defined CSE medium, or CSE medium supplemented with 0.1% or 1% casamino acids (CAA) as indicated in the legend. Luminescence output, expressed as relative light units per OD_600_ (RLU/OD), was monitored over time. Results are shown as the mean and standard error of the mean of two experiments. The approximate extent of the different growth phases is indicated above the graph; trans., transition phase.


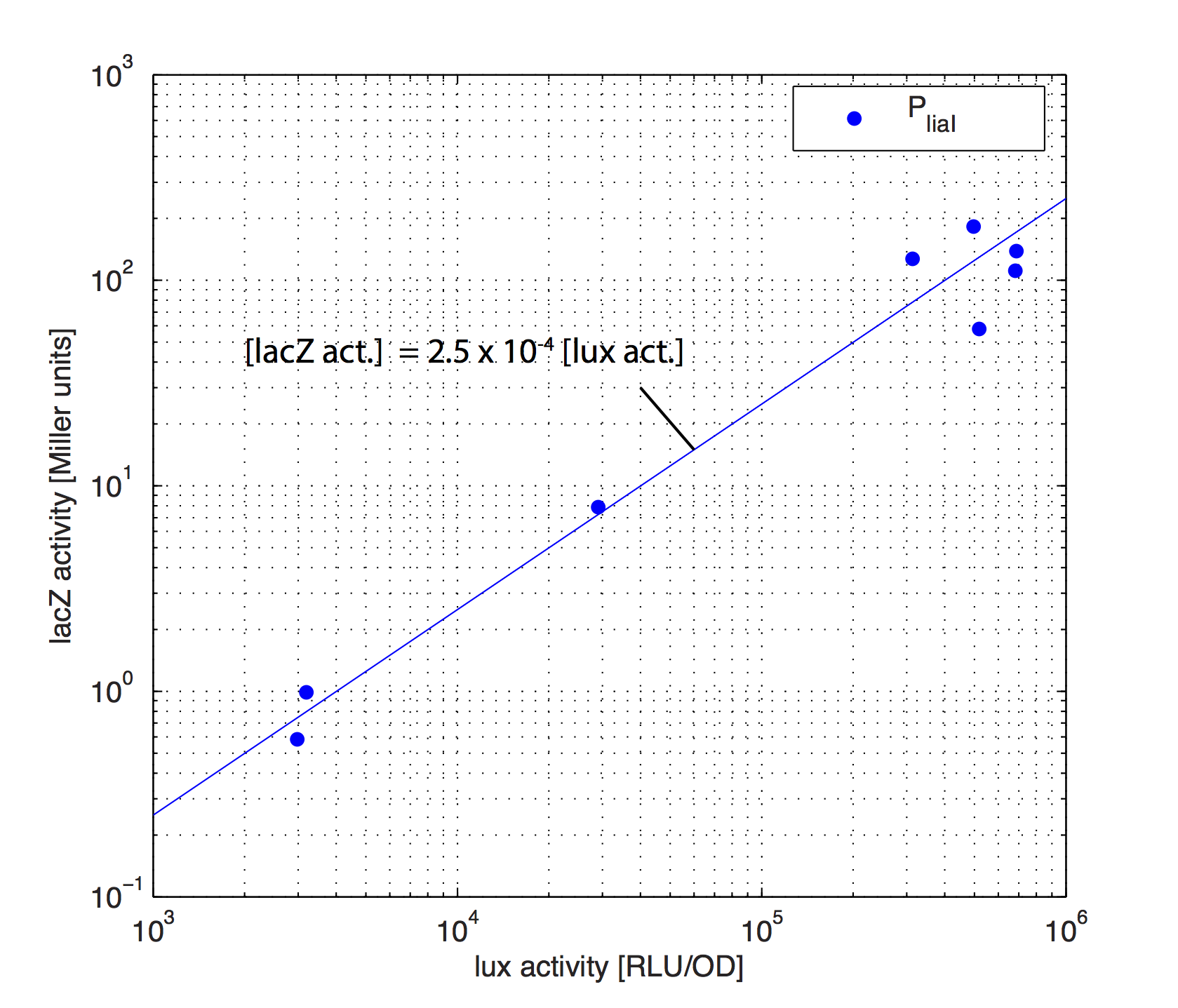


Figure S2. Correlation between reporter output of lacZ and lux.

The strains TMB1858 (P*_liaI_*-*lux*) and TMB1857 (P*_liaI_*-*lacZ*) were grown in LB medium and induced with the bacitracin concentrations 0, 0.1, 0.3, 1, 3, 10, 30 and 100 μg ml^‑1^. The respective activities show a linear correlation 30 min after induction, as is expected even though the *lacZ* activity equilibrates on a timescale much longer than the luciferase signal. In fact, when measuring the *lacZ* activity under two different conditions with protein expression rates α_1_ and α_2_ but, importantly, at the same time T, the fold-change between the protein levels directly reflects the fold-change of the expression rates: Given that the LacZ protein level, Z(t), exponentially approaches its steady state at a timescale given by the cell doubling rate δ, Z(t) ~ α/δ*[1-exp(-δ*t)], the ratio of the protein levels is independent of time, i.e., Z_1_(T)/Z_2_(T) = α_1_/α_2_. Therefore, we expect a linear correlation between luciferase and *lacZ* activities even if the latter has not yet reached its steady state level at the reference time point.

**
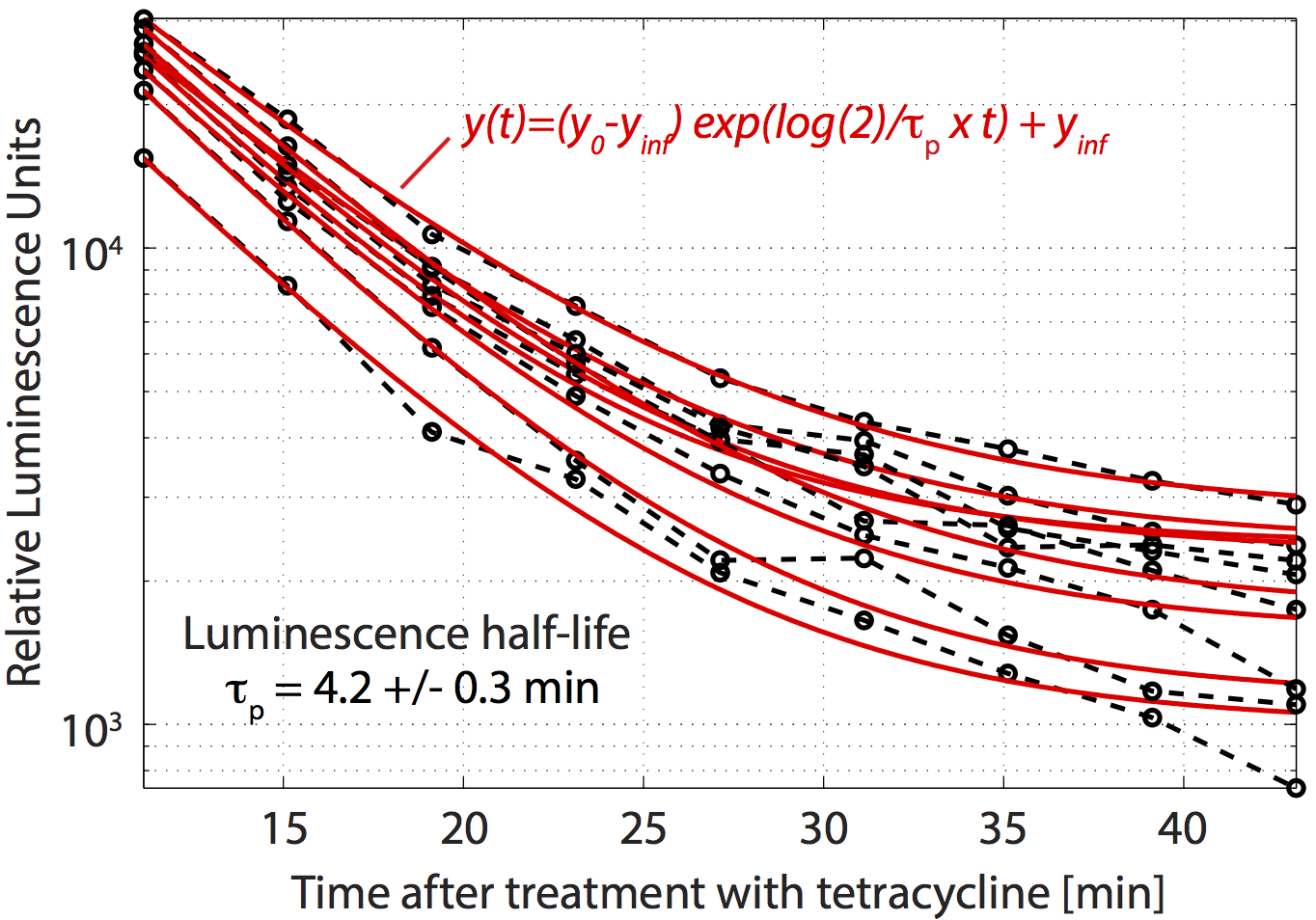
**

Figure S3. Determination of luminescence half-life.

To determine the half-life τ_p_ of the output of the luciferase reporter system, *B. subtilis* harboring the P*_xyl_*-*luxABCDE* reporter construct was grown in CSE medium in the presence of 0.15 % (w/v) xylose under the conditions described for luciferase assays with constitutive promoters. When luciferase activities reached approximately 10^5^ RLU/OD_600_ (early exponential phase), further protein synthesis was stopped by the addition of 500 µg ml^-1^ tetracycline, and luminescence and OD_600_ were monitored every 5 min. The half-life of the luminescence output was determined from a fit of the data from eight replicate assays (*symbols*) with an exponential decay function (*red lines*).


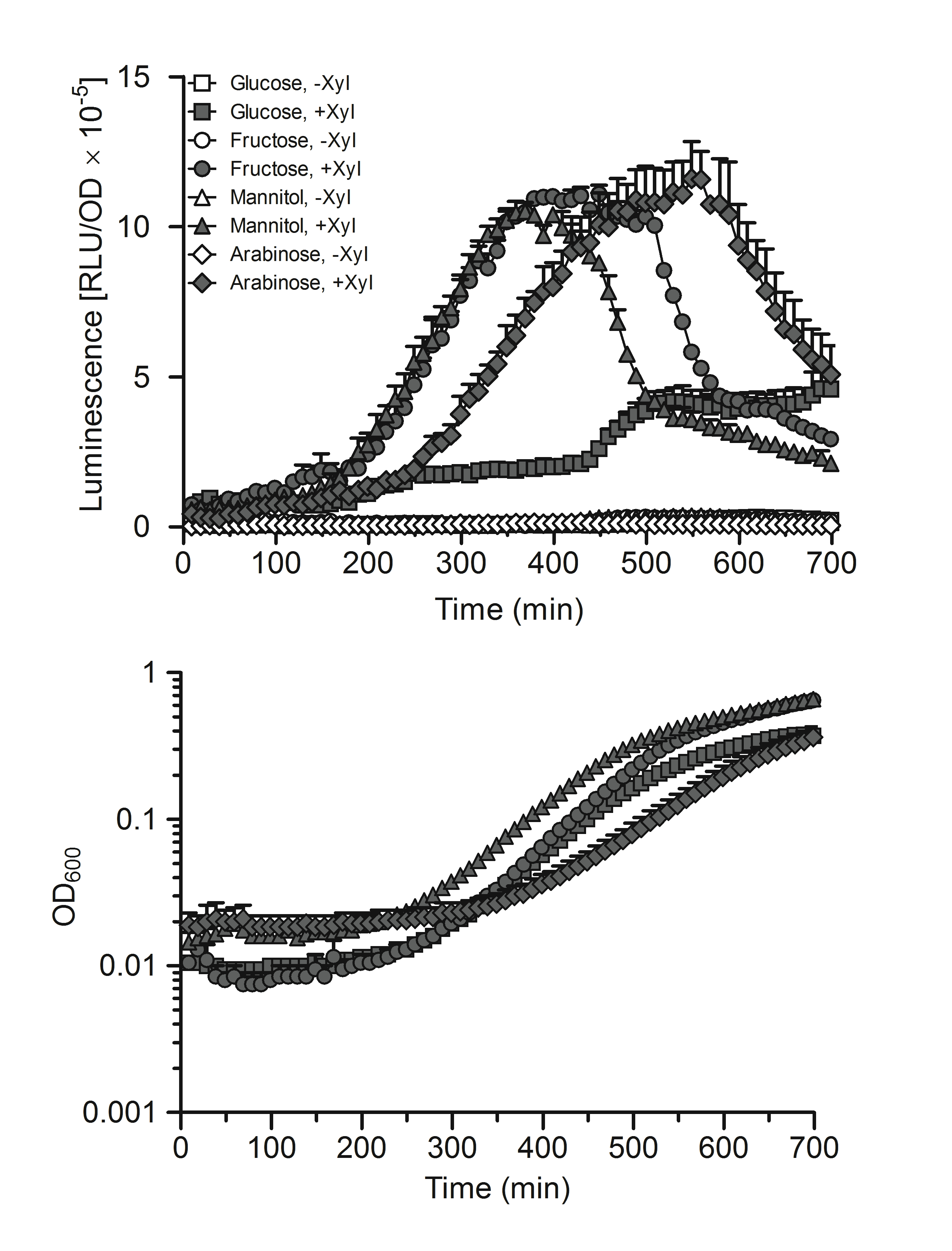


Figure S4: Effects of different carbon sources on xylose-dependent induction of P*_xylA_*.

Wild-type *B. subtilis* carrying the P*_xylA_*-*luxABCDE* reporter construct was grown in defined CSE medium supplemented with 2.5 % of different carbon sources in the presence or absence of 0.2 % xylose (Xyl) as indicated in the legend. Luminescence output, expressed as relative light units per OD_600_ (RLU/OD, top panel) and growth (OD_600_, bottom panel), were monitored over time. Results are shown as the mean and standard error of the mean of two experiments.

## Protocols

**Media**

## Luria-Bertani (LB) broth:

|  | Tryptone | 10 g |
| --- | --- | --- |
|  | Yeast extract | 5 g |
|  | NaCl | 10 g |
|  | H2O (dest) | ad 1.000 ml |

- for LB plates: add 15 g/l of agar
  - important:cool down the agar solution to 50°C before adding antibiotics

## Starch plates:

|  | Nutrient Broth (Difco) | 7,5 g |
| --- | --- | --- |
|  | Starch | 5 g |
|  | Agar | 15 g |
|  | H2O (dest) | ad 1.000 ml |

## Chemical defined medium (CSE): (100ml)

|  | 5×C-Salts | 20 ml |
| --- | --- | --- |
|  | Tryptophan (5 mg/ml) | 1 ml |
|  | Ammoniumeisencitrat (2,2 mg/ml) | 1 ml |
|  | III’-Salts | 1 ml |
|  | Potassium glutamate (40%) | 2 ml |
|  | Sodium succinate (30%) | 2 ml |
| 5×C-Salts (1 l) | | |
|  | KH_2_PO_4_ | 20 g |
|  | K_2_HPO_4_ × 3 H_2_O | 80 g |
|  | (NH_4_)_2_SO_4_ | 16,5 g |
| III’-Salts (1 l) | | |
|  | MnSO_4_ × 4 H_2_O | 0,232 g |
|  | MgSO_4_ × 7 H_2_O | 12,3 g |

- autoclave (or filtrate) each component separately and put them together freshly before starting your experiment
- Optionally: addition of media additives, for example pyruvate (0.5% final concentration) or glucose (1% final concentration)

## MOPS-based chemically defined medium (MCSE) (100ml)

|  | 10×MOPS solution | 10 ml |
| --- | --- | --- |
|  | Tryptophan (5 mg/ml) | 1 ml |
|  | Ammonium ferric citrate (2,2 mg/ml) | 1 ml |
|  | III’-Salts | 1 ml |
|  | Potassium glutamate (40%) | 2 ml |
|  | Sodium succinate (30%) | 2 ml |
|  | Fructose (20%) | 1 ml |
| 10x MOPS solution (1 l), adjust pH = 7 with KOH (10 M)  ( 400 mM MOPS, 10 mM phosphate) | | |
|  | MOPS | 83,72 g |
|  | KH_2_PO_4_ (1M) | 3,85 ml |
|  | K_2_HPO_4_ (1M) | 6,15 ml |
|  | (NH_4_)_2_SO_4_ | 33 g |
| III’-Salts (1 l) | | |
|  | MnSO_4_ × 4 H_2_O | 0,232 g |
|  | MgSO_4_ × 7 H_2_O | 12,3 g |

- autoclave (or filtrate) each component separately and put them together freshly before starting your experiment
- Optionally: addition of media additives, for example pyruvate (0.5% final concentration) or glucose (1% final concentration)

## Antibiotics

- Indicated are 1.000-times stock solutions
- Dissolve in the specific solvent and filtrate by using 0.2 µm filters
- Store at -20°C

| Strain | Antibiotic | Concentration | Dissolve in | Color code |
| --- | --- | --- | --- | --- |
| *B. subtilis* | Kanamycin | 10 mg/ml | H_2_O | Black (one bar) |
|  | Chloramphenicol | 5 mg/ml | 70% ethanol | Blue |
|  | MLS selection: |  |  | Red |
|  | Erythromycin | 1mg/ml | 70% ethanol |  |
|  | Linkomycin | 25 mg/ml | H_2_O |  |
|  | Spectinomycin | 100 mg/ml | H_2_O | Purple |
|  | Bacitracin | 50 mg/ml | H_2_O | - |
|  |  |  |  |  |
| *E. coli* | Ampicillin | 100 mg/ml | H_2_O | Green |

## QuikChange Site Directed Mutagenesis

<http://www.genomics.agilent.com/files/Manual/200523.pdf>

- **Primer Design Guidelines**
  - Both of the mutagenic primers must contain the desired mutation and anneal to the same sequence on opposite strands of the plasmid.
  - Primers should be between 25 and 45 bases in length, with a melting temperature (Tm) of ≥78°C. Primers longer than 45 bases may be used, but using longer primers increases the likelihood of secondary structure formation, which may affect the efficiency of the mutagenesis reaction.
  - The following formula is commonly used for estimating the *T*m of primers:

T_m_ = 81.5 + 0.41(%GC) - (675/N) - % mismatch

- - - *N* is the primer length in bases
    - values for **%GC** and **% mismatch** are whole numbers
  - For calculating *T*m for primers intended to introduce insertions or deletions, use this modified version of the above formula:

T_m_ = 81.5 + 0.41(%GC) - (675/N)

where N does not include the bases which are being inserted or deleted.

- - The desired mutation (deletion or insertion) should be in the middle of the primer with ~10–15 bases of correct sequence on both sides.
  - The primers optimally should have a minimum GC content of 40% and should terminate in one or more C or G bases.
- **PCR Reaction**
- Use 125 ng of each primer. To convert nanograms to picomoles of oligo, use the following equation:

X pmoles of oligo = (ng of oligo)/(330 x #of bases in oligo) x 1000

For example, for 125 ng of a 25-mer:

(125 ng of oligo)/(330 x 25 bases) x 1000 = 15 pmole

- Use standard Phusion PCR protocol with following modifications:

1. elongation time ~1 minute for 1 kb
2. 12 cycles (up to 35)
3. Annealing temperature 60°C (down to 52)

It usually works well to try different template DNA concentrations (e.g. 5, 10, 20 and 50 ng).

As a control, prepare a reaction without Phusion (should give no colonies)

- **DpnI digest**

1 µl DpnI/PCR reaction

Incubate 60 min at 37°C

- ***E. coli* transformation**

According to a standard protocol, with 10 µl PCR reaction

## Plasmid Extraction from *E. coli* - Alkaline Lysis Method

- Harvest 2-4 ml of cells in eppendorf (13,000rpm, 1 min) Decant supernatant (aspirate)
- Resuspend cells in 300 µl P1 buffer to a homogenous suspension
- Add 300 µl of lysis buffer (P2 buffer), invert about 6 times (not more!)
- Add 300 µl K-Ac/5% formic acid and invert tube approx 6 times. Should see a precipitate form
- Spin at 13,000 rpm for 10 min then transfer supernatant into new eppendorf
- Precipitate plasmid DNA in 0.7 vol (i.e. 630 µl) of room temperature isopropanol and invert about 6 times
- Spin at 13,000 rpm for 15mins and decant supernatant.
- Wash pellet in 70% ethanol (ca. 700 µl) and remove supernatant, spin again if pellet becomes dislodged.
- Quick spin to remove final trace ethanol and allow pellet to air dry (approx 10-15 mins)
- Dissolve DNA in 50-100 µl of MQ H_2_0 (pH5.5) or 10 mM Tris/HCl (pH8.0).

**Recipes:**

**P1 Buffer** (Recipe from Qiagen kit) **(store in fridge)**

50mM Tris/HCl [pH 8]

10mM EDTA [pH 8]

Make up part of the final volume with the Tris/HCl and EDTA solutions with water.

100μg/ml DNase-free RNase (from 10 mg/ml stock)

Lysis Buffer (P2) (store at RT, but only make about 10 or 20 ml as it doesn’t keep forever)

0.2M NaOH

1% SDS

**K Acetate/5% formic acid** (store at RT)

88.3g K-acetate

15ml Formic Acid

300ml volume with dH_2_0

## Transformation of *Bacillus subtilis* (simple)

• inoculate 10 ml MNGE to OD_600_ = 0,1 (or simply 1/100) from overnight culture

• let grow to OD_600_ = 1.1-1.3 at 37°C with agitation (at least 200 rpm!)

• use 400 μl cells for transformation (in test-tube, not eppendorf!):

o add DNA (ca. 1-2 µg linearized plasmid or 100 µl crude-prep genomic DNA)

o let grow for 1 h

o add 100 µl Expression Mix (may need to pre-induce: Ery 0,025 μg/ml, Cm 0,125 μg/ml)

o let grow for 1 h

o plate on selective media

**10 X MN-Medium:**

136 g K_2_HPO_4_ (x 3 H_2_O)

60 g KH_2_PO_4_

10 g Na-citrat (x 2 H_2_O)

**MNGE-Medium:**

9,2 ml 1 x MN-Medium (920 µl 10x MN + 8,28 ml sterile water)

1 ml Glucose (20%)

50 µl K-Glutamat (40%)

50 µl Fe[III]- ammonium-citrate (2,2 mg/ml)

100 µl Tryptophan (5 mg/ml)

30 µl MgSO_4_ (1M)

(100 µl threonine (5 mg/ml) for strains carrying an insertion in *thrC*)

**Expression Mix:**

500 µl yeast extract (5%)

250 µl casamino-acids (CAA) (10%)

250 µl H_2_O

50 µl Tryptophan (5 mg/ml)

Check for integration: see pages 27-30

| Competent *E. coli* cells |
| --- |
| From openwetware: <http://openwetware.org/wiki/TOP10_chemically_competent_cells> |

Overview

This protocol is a variant of the Hanahan protocol [[1](http://partsregistry.org/Help:Protocols/Competent_Cells#bibkey_Hanahan91)] using CCMB80 buffer for DH10B, TOP10 and MachI strains. It builds on Example 2 of the [Bloom05 patent](http://openwetware.org/images/b/bd/Pat6855494.pdf) as well. This protocol has been tested on NEB10, TOP10, MachI and [BL21(DE3)](http://openwetware.org/wiki/Talk:TOP10_chemically_competent_cells) cells. See [OWW Bacterial Transformation page](http://openwetware.org/wiki/Bacterial_Transformation) for a more general discussion of other techniques. The [Jesse '464 patent](http://openwetware.org/images/0/0c/Pat6960464.pdf) describes using this buffer for DH5α cells. The [Bloom04](http://openwetware.org/images/c/c2/Pat6709852.pdf) patent describes the use of essentially the same protocol for the Invitrogen Mach 1 cells.

**This is the chemical transformation protocol used by**[**Tom Knight**](http://partsregistry.org/wiki/index.php?title=User:Tk&action=edit)**and the**[**Registry of Standard Biological Parts**](http://partsregistry.org/)**.**

Materials

- Detergent-free, sterile glassware and plasticware (see procedure)
- Table-top OD600nm spectrophotometer
- [SOB](http://partsregistry.org/SOB)

**CCMB80 buffer**

- 10 mM KOAc pH 7.0 (10 ml of a 1M stock/L)
- 80 mM CaCl_2_.2H_2_O (11.8 g/L)
- 20 mM MnCl_2_.4H_2_O (4.0 g/L)
- 10 mM MgCl_2_.6H_2_O (2.0 g/L)
- 10% glycerol (100 ml/L)
- adjust pH DOWN to 6.4 with 0.1N HCl if necessary
  - adjusting pH up will precipitate manganese dioxide from Mn containing solutions.
- sterile filter and store at 4°C
- slight dark precipitate appears not to affect its function

Procedure

**Preparing glassware and media**

**Eliminating detergent**

Detergent is a major inhibitor of competent cell growth and transformation. Glass and plastic must be detergent free for these protocols. The easiest way to do this is to avoid washing glassware, and simply rinse it out. Autoclaving glassware filled 3/4 with DI water is an effective way to remove most detergent residue. Media and buffers should be prepared in detergent free glassware and cultures grown up in detergent free glassware.

**Prechill plasticware and glassware**

Prechill 250mL centrifuge tubes and screw cap tubes before use.

**Preparing seed stocks**

- Streak TOP10 cells on an [SOB](http://partsregistry.org/SOB) plate and grow for single colonies at 23°C [we use XL1 blue]
  - room temperature works well
- Pick single colonies into 2 ml of SOB medium and shake overnight at 23°C
  - room temperature works well
- Add glycerol to 15%
- Aliquot 1 ml samples to Nunc cryotubes
- Place tubes into a zip lock bag, immerse bag into a dry ice/ethanol bath for 5 minutes
  - This step may not be necessary
- Place in -80°C freezer indefinitely.

**Preparing competent cells**

- Ethanol treat all working areas for sterility.
- Inoculate 250 ml of [SOB](http://partsregistry.org/SOB) medium with 1 ml vial of seed stock and grow at 20°C to an OD600nm of 0.3. Use the "cell culture" function on the Nanodrop to determine OD value. OD value = 600nm Abs reading x 10
  - This takes approximately 16 hours.
  - Controlling the temperature makes this a more reproducible process, but is not essential.
  - Room temperature will work. You can adjust this temperature somewhat to fit your schedule
  - Aim for lower, not higher OD if you can't hit this mark
- Fill an ice bucket halfway with ice. Use the ice to pre-chill as many flat bottom centrifuge bottles as needed.
- Transfer the culture to the flat bottom centrifuge tubes. Weigh and balance the tubes using a scale
  - Try to get the weights as close as possible, within 1 gram.
- Centrifuge at 3000g at 4°C for 10 minutes in a flat bottom centrifuge bottle.
  - Flat bottom centrifuge tubes make the fragile cells much easier to resuspend
- Decant supernatant into waste receptacle, bleach before pouring down the drain.
- Gently resuspend in 80 ml of ice cold CCMB80 buffer
  - Pro tip: add 40ml first to resuspend the cells. When cells are in suspension, add another 40ml CCMB80 buffer for a total of 80ml
  - Pipet buffer against the wall of the centrifuge bottle to resuspend cells. Do not pipet directly into cell pellet!
  - After pipetting, there will still be some residual cells stuck to the bottom. Swirl the bottles gently to resuspend these remaining cells
- Incubate on ice for 20 minutes
- Centrifuge again at 3000G at 4°C. Decant supernatant into waste receptacle, and bleach before pouring down the drain.
- Resuspend cell pellet in 10 ml of ice cold CCMB80 buffer.
  - If using multiple flat bottom centrifuge bottles, combine the cells post-resuspension
- Use Nanodrop to measure OD of a mixture of 200 μl SOC and 50 μl of the resuspended cells
  - Use a mixture of 200 μl SOC and 50 μl CCMB80 buffer as the blank
- Add chilled CCMB80 to yield a final OD of 1.0-1.5 in this test.
- Incubate on ice for 20 minutes. Prepare for aliquoting
  - Make labels for aliquots. Use these to label storage microcentrifuge tubes/microtiter plates
  - Prepare dry ice in a separate ice bucket. Pre-chill tubes/plates on dry ice.
- Aliquot into chilled 2ml microcentrifuge tubes or 50 μl into chilled microtiter plates
- Store at -80°C indefinitely.
  - Flash freezing does not appear to be necessary
- Test competence (see below)
- Thawing and refreezing partially used cell aliquots dramatically reduces transformation efficiency by about 3x the first time, and about 6x total after several freeze/thaw cycles.

**Measurement of competence**

- Transform 50 μl of cells with 1 μl of standard pUC19 plasmid (Invitrogen) (we use pSB1A3)
  - This is at 10 pg/μl or 10^-5^ μg/μl
  - This can be made by diluting 1 μl of NEB pUC19 plasmid (1 μg/μl, NEB part number N3401S) into 100 ml of TE
- Incubate on ice 0.5 hours. Pre-heat water bath now.
- Heat shock 60 sec at 42C
- Add 250 μl [SOC](http://partsregistry.org/SOC)
- Incubate at 37 C for 1 hour in 2 ml centrifuge tubes, using a mini-rotator
  - Using flat-bottomed 2ml centrifuge tubes for transformation and regrowth works well because the small volumes flow well when rotated, increasing aeration.
  - For our plasmids (pSB1AC3, pSB1AT3) which are chloramphenicol and tetracycline resistant, we find growing for 2 hours yields many more colonies
  - Ampicillin and kanamycin appear to do fine with 1 hour growth
- Add 4-5 sterile 3.5mm glass beads to each agar plate, then add 20 μl of transformation
  - After adding transformation, gently move plates from side to side to re-distribute beads. When most of transformation has been absorbed, shake plate harder
  - Use 3 plates per vial tested
- Incubate plates agar-side up at 37 C for 12-16 hours
- Count colonies on light field the next day
  - Good cells should yield around 100 - 400 colonies
  - Transformation efficiency is (dilution factor=15) x colony count x 10^5^/µgDNA
  - We expect that the transformation efficiency should be between 1.5x10^8^ and 6x10^8^ cfu/µgDNA

References

1. [Hanahan D, Jessee J, and Bloom FR. *Plasmid transformation of Escherichia coli and other bacteria.* Methods Enzymol 1991; 204 63-113.](http://eutils.ncbi.nlm.nih.gov/entrez/eutils/elink.fcgi?cmd=prlinks&dbfrom=pubmed&retmode=ref&id=1943786) pmid:1943786.[PubMed](http://www.ncbi.nlm.nih.gov/entrez/query.fcgi?cmd=Retrieve&db=pubmed&dopt=Abstract&list_uids=1943786) [HubMed](http://www.hubmed.org/display.cgi?uids=1943786) [Hanahan91]
2. [Reusch RN, Hiske TW, and Sadoff HL. *Poly-beta-hydroxybutyrate membrane structure and its relationship to genetic transformability in Escherichia coli.* J Bacteriol 1986 Nov; 168(2) 553-62.](http://eutils.ncbi.nlm.nih.gov/entrez/eutils/elink.fcgi?cmd=prlinks&dbfrom=pubmed&retmode=ref&id=3536850) pmid:3536850. [PubMed](http://www.ncbi.nlm.nih.gov/entrez/query.fcgi?cmd=Retrieve&db=pubmed&dopt=Abstract&list_uids=3536850) [HubMed](http://www.hubmed.org/display.cgi?uids=3536850) [Reusch86]
3. [Addison CJ, Chu SH, and Reusch RN. *Polyhydroxybutyrate-enhanced transformation of log-phase Escherichia coli.* Biotechniques 2004 Sep; 37(3) 376-8, 380, 382.](http://eutils.ncbi.nlm.nih.gov/entrez/eutils/elink.fcgi?cmd=prlinks&dbfrom=pubmed&retmode=ref&id=15470891) pmid:15470891. [PubMed](http://www.ncbi.nlm.nih.gov/entrez/query.fcgi?cmd=Retrieve&db=pubmed&dopt=Abstract&list_uids=15470891) [HubMed](http://www.hubmed.org/display.cgi?uids=15470891) [Addison04]
4. US Patent 6,709,852 [pat6709852.pdf](http://openwetware.org/images/c/c2/Pat6709852.pdf)

[Bloom04]

1. US Patent 6,855,494 [pat6855494.pdf](http://openwetware.org/images/b/bd/Pat6855494.pdf)

[Bloom05]

1. US Patent 6,960,464 [pat6960464.pdf](http://openwetware.org/images/0/0c/Pat6960464.pdf)

All Medline abstracts: [PubMed](http://www.ncbi.nlm.nih.gov/entrez/query.fcgi?cmd=Retrieve&db=pubmed&dopt=Abstract&list_uids=1943786,3536850,15470891) [HubMed](http://www.hubmed.org/display.cgi?uids=1943786,3536850,15470891)

## β-Galactosidase Assay for *B. subtilis* (based on Miller, 1972)

**Example of culture preparation**

- Inoculate LB medium 1:100 with a fresh overnight culture carrying a promoter-*lacZ*-fusion and incubate on a shaker at 37°C
- At OD_600_ 0.4-0.5 split the culture into 2 ml samples, induce one sample with e. g. an antibiotic, leave one sample as an uninduced control
- After 30 min, harvest cells by centrifugation and store the pellet at -20°C or continue directly with the assay

**β-Galactosidase Assay**

- Resuspend the cell pellet in 1 ml working buffer
- In a cuvette dilute the samples with working buffer until OD_600_ is between 0.2 and 0.8 in a final volume of 800 µl (usually 500 µl working buffer and 300 µl of cells)
- Measure OD_600_, use 800 µl working buffer as blank
- Add 10 µl Lysozyme, vortex and incubate at 37°C for 15-45 min, check if the sample is clear
- Add 150 µl ONPG, mix well and record time (=t_0_)
- Incubate at room temperature until the sample turns yellow
- Stop the reaction by adding 400 µl Na_2_CO_3_, mix well and record time (=t_s_)
- If the samples do not turn yellow, stop the reaction after 60 min
- Measure OD_420_ and OD_550_ of each sample, use a cuvette with everything but the cells as blank
- Calculate promoter activity according to the formula:

$$Miller Units=\frac{1000 * \left(A420-\left(1,75 * OD550\right)\right)}{\left(t * v * OD600\right)}$$

A420 absorption at 420 nm t time of reaction (T_s_ - T_0_)

A550 absorption at 550 nm v volume of sample (usually 0.8 ml)

A600 absorption at 600 nm

**Solutions**

Lysozyme 15 mg/ml in Z-buffer

Na_2_CO_3_ 1 M

ONPG (2-nitrophenyl-β-D-galactopyranoside) 4 mg/ml in Z-buffer

Z-buffer (pH 7.0) Na_2_HPO_4_ * 2 H_2_O 60 mM 10.68 g

NaH_2_PO_4_ * H_2_O 40 mM 5.52 g

KCl 10 mM 0.75 g

MgSO_4_ * 7 H_2_O 1 mM 0.24 g

H_2_O ad 1000 ml

Working buffer (prepare fresh) Z-buffer

20 mM β-Mercaptoethanol (150 µl to 100 ml Z-buffer)

## Western blot detection of GFP

The membrane was incubated with primary or secondary antibodies either in a 5 ml solution in a 50 ml falcon tube, or in 1 ml solution between two plastic foil sheets.

| Shake membrane overnight in Blotto at 4°C (in a flat-bottom bowl)  Primary antibody:   - Dilute anti-GFP (Epitomics, No. 1533-1, rabbit) 1:3000 in Blotto (1.6 μl in 5 ml or 0.3 μl in 1ml) - incubate for 1 h at RT   Wash:   - 4× 10 min in 5 ml Blotto (50 ml Falcon)   Secondary antibody:   - Dilute Anti-rabbit-HRP (Promega, W401B) 1:2000 in Blotto (2.5 μl in 5 ml or 0.4 μl in 1 ml) - incubate for 1 h at RT   Wash:   - 4× 10 min in 5 ml Blotto (50 ml Falcon) - in a flat-bottom bowl 🡪 wash ca. 5 min in 1×TBS   Detection:   - Ace Glow: mix two solutions 1:1 (final: 300 µl for half a blot 🡪 150 +150 µl) - incubate shortly (few min) - Detection of luminescence with LumiImager   **Puffer:**   \|  \|  \|  \|  \| \| --- \| --- \| --- \| --- \| \| 10×TBS (1 L) \| Tris-HCl (pH 7.6) \| 500 mM \| 60.6 g \| \|  \| NaCl \| 1.5 M \| 88 g \| \|  \| dH_2_O \|  \| ad 1 L \| \|  \|  \|  \|  \| \| Blotto (1 L) \| Skim milk powder \| 2.5% \| 25 g \| \|  \| 10×TBS \| 1× \| 100 ml \| \|  \| dH_2_O \|  \| ad 1 L \| |
| --- | --- | --- | --- | --- | --- | --- | --- | --- | --- | --- | --- | --- | --- | --- | --- | --- | --- | --- | --- | --- | --- | --- | --- | --- | --- | --- | --- | --- | --- | --- | --- | --- |

| Shake membrane overnight at 4°C (in a flat-bottom bowl)  Primary antibody:   - Dilute Anti-FLAG (Sigma, Anti-Flag polyclonal, F7425, rabbit) 1:2000 in Blotto (2.5 μl in 5 ml or 0,4 μl in 1 ml) - incubate for 1 h at RT   Wash:   - 4× 10 min in 5 ml Blotto (50 ml Falcon)   Secondary antibody:   - Dilute Anti-rabbit-HRP (Promega, W401B) 1:2000 in Blotto (2.5 μl in 5 ml or 0.4 μl in 1 ml) - incubate for 1 h at RT   Wash:   - 4× 10 min in 5 ml Blotto (50 ml Falcon) - in a flat-bottom bowl 🡪 wash ca. 5 min in 1×TBS   Detection:   - Ace Glow: mix two solutions 1:1 (final: 300 µl for half a blot 🡪 150 +150 µl) - incubate shortly (few min) - Detection of luminescence with LumiImager   **Puffer:**   \| 10×TBS (1 L) \| Tris-HCl (pH 7.6) \| 500 mM \| 60.6 g \| \| --- \| --- \| --- \| --- \| \|  \| NaCl \| 1.5 M \| 88 g \| \|  \| dH_2_O \|  \| ad 1 L \| \|  \|  \|  \|  \| \| Blotto (1 L) \| Skim milk powder \| 2.5% \| 25 g \| \|  \| 10×TBS \| 1× \| 100 ml \| \|  \| dH_2_O \|  \| ad 1 L \| \|  \|  \|  \|  \| |
| --- | --- | --- | --- | --- | --- | --- | --- | --- | --- | --- | --- | --- | --- | --- | --- | --- | --- | --- | --- | --- | --- | --- | --- | --- | --- | --- | --- | --- | --- | --- | --- | --- |

## Detection of Flag-tag on Western blots

## Detection of His-tag on Western Blots

| Incubate membrane overnight in 1xTBS (3% BSA) at 4°C  Primary antibody:   - Dilute Anti-Penta-His (Qiagen, Penta-His, No. 34660, mouse) 1:2000 in 1xTBS (+5% BSA) (2.5 μl in 5 ml or 0.4 μl in 1 ml) - incubate for 1 h at RT   Wash:   - 2× 10 min in 5 ml 1x TBS (0.1% Tween20) - 1× 10 min in 5 ml 1x TBS   Sekundary antibody:   - Dilute Anti-mouse-HRP (Promega, W402B1) 1:2000 in 1xTBS (10% milk) (2.5 μl in 5 ml or 0.4 μl in 1 ml) - incubate for 1 h at RT   Wash:   - 4× 10 min in 5 ml 1x TBS (0,1% Tween20) - in a flat-bottom bowl 🡪 wash ca. 5 min in 1×TBS   Detection:   - Ace Glow: mix two solutions 1:1 (final: 300 µl for half a blot 🡪 150 +150 µl) - incubate shortly (few min) - Detection of luminescence with LumiImager   **Puffer:**   \| 10×TBS (1 L) \| Tris-HCl (pH 7.6) \| 500 mM \| 60.6 g \| \| --- \| --- \| --- \| --- \| \|  \| NaCl \| 1.5 M \| 88 g \| \|  \| dH_2_O \|  \| ad 1 L \| \|  \|  \|  \|  \| \|  \|  \|  \|  \| |
| --- | --- | --- | --- | --- | --- | --- | --- | --- | --- | --- | --- | --- | --- | --- | --- | --- | --- | --- | --- | --- |

## Detect strep-tag on Western blots with Strep-Tactin-HRP conjugate (IBA)

| Material:   - PBS buffer: 4 mM KH_2_PO_4_; 16 mM Na_2_HPO_4_; 115 mMNaCl; pH 7.4 - PBS-blocking buffer: PBS buffer with 3 % BSA and 0.5 % v/v Tween20 - Enzyme dilution buffer: PBS with 0.2 % BSA and 0.1 % v/v Tween20 - PBS-Tween buffer: PBS with 0.1 % Tween20 - *Strep*-tag protein ladder (-20°C, aliquots) can be used as positive control - For blocking biotinylated proteins use Biotin Blocking buffer (4°C fridge) - Chemiluminescence detection solution (Ace Glow (Pelab), 4°C, fridge)  1. After transfer the proteins to the membrane, block the membrane in 20 ml PBS-blocking buffer. Incubate for 1 h (room temperature) or overnight (4°C) with gentle shaking 2. Wash 3 times with 20 ml PBS-Tween buffer (each step: 5 minutes, room temperature, gentle shaking) 3. After last washing step, add 10 ml PBS-Tween buffer to the membrane 4. Optional: Before detection *Strep*-tag proteins add 10 µl Biotin Blocking buffer (10 minutes, room temperature, gentle shaking 5. Pre-dilute *Strep*-Tactin-HRP conjugate (IBA, Strep-Tactin-HRP conjugate, No. 2-1502-001) 1:100 in Enzyme dilution buffer (PBS, BSA, Tween) and add 10 µl to 10 ml PBS-Tween. Incubate 1 hour, room temperature, gentle shaking) 6. Wash 2 times with PBS-Tween buffer (each step: 1 min, room temperature, gentle shaking) 7. Wash 2 times with PBS buffer (each step: 1 min, room temperature, gentle shaking) 8. Develop chemiluminescence reaction according to the instructions of Peqlab for Ace Glow |
| --- |

## Detect HA-tag on Western blots

| Incubate blot overnight in TBS (+0.05% Tween20/ 5% milk) at 4°C (in a flat-bottom-bow), shaking  Primary abtibody:   - dilute Anti-HA **(Sigma, H6908)** **1:500** in 1 ml TBS (+0.05% Tween20/ 5% milk) (1.4 µl AK in 700 µl) 🡪 pipette onto the membrane   incubate for1h at RT  Wash:   - put Membran into 50 ml-Falcon - wash 4× 10 min mit 5 ml 1xTBS/0.05% Tween20   Sekundary antibody:   - dilute Anti-rabbit-HRP (Promega, W401B) 1:2000 in 1 ml Blotto (0.4 µl AK in 1 ml) 🡪 pipette onto membrane - incubate for1 h at RT   Wash:   - 4× 10 min in 5 ml Blotto - put membrane into flat-bottom box 🡪 wash ca. 5 min in 1xTBS   Detection:   - Ace Glow: mix two solutions 1:1 (final: 300 µl for half a blot 🡪 150 +150 µl) - incubate shortly (few min) - Detection of luminescence with LumiImager   **Puffer:**   \| 10×TBS (1 L) \| Tris-HCl (pH 7.6) \| 500 mM \| 60.6 g \| \| --- \| --- \| --- \| --- \| \|  \| NaCl \| 1.5 M \| 88 g \| \|  \| dH_2_O \|  \| ad 1 L \| \| Blotto (1 L) \| Skim milk powder \| 2.5% \| 25 g \| \|  \| 10x TBS \| 1x \| 100 ml \| \|  \| dH_2_O \|  \| ad 1 L \| |
| --- | --- | --- | --- | --- | --- | --- | --- | --- | --- | --- | --- | --- | --- | --- | --- | --- | --- | --- | --- | --- | --- | --- | --- | --- |

## Detection of cMyc on Western blots

| Incubate blot overnight in TBS (+0.05% Tween20/ 5% milk) at 4°C (in a flat-bottom-bow)l, shaking  Primary abtibody:   - dilute Anti-Myc **(Abcan, ab9106)** 1:2000 in 1 ml TBS (+0.05% Tween20/ 5% milk) (0.4 µl AK in 1 ml) 🡪 pipette onto the membrane   incubate for1h at RT  Wash:   - put Membran into 50 ml-Falcon - wash 4× 10 min mit 5 ml 1xTBS/0.05% Tween20   Sekundary antibody:   - dilute Anti-rabbit-HRP (Promega, W401B) 1:2000 in 1 ml Blotto (0.4 µl AK in 1 ml) 🡪 pipette onto membrane - incubate for1 h at RT   Wash:   - 4× 10 min in 5 ml Blotto - put membrane into flat-bottom box 🡪 wash ca. 5 min in 1xTBS   Detection:   - Ace Glow: mix two solutions 1:1 (final: 300 µl for half a blot 🡪 150 +150 µl) - incubate shortly (few min) - Detection of luminescence with LumiImager   **Puffer:**   \| 10×TBS (1 L) \| Tris-HCl (pH 7.6) \| 500 mM \| 60.6 g \| \| --- \| --- \| --- \| --- \| \|  \| NaCl \| 1.5 M \| 88 g \| \|  \| dH_2_O \|  \| ad 1 L \| \| Blotto (1 L) \| Magermilchpulver \| 2.5% \| 25 g \| \|  \| 10x TBS \| 1x \| 100 ml \| \|  \| dH_2_O \|  \| ad 1 L \| |
| --- | --- | --- | --- | --- | --- | --- | --- | --- | --- | --- | --- | --- | --- | --- | --- | --- | --- | --- | --- | --- | --- | --- | --- | --- |

## How to work with *Bacillus subtilis* vectors

Many vectors for *Bacillus subtilis* are integrating into the genome, so do all but one that are provided by the BioBrick Box. There are some features in *B. subtilis* vectors that have to be taken into account, while working with them.

- Cloning in *E.coli*
- Linearisation before *B. subtilis* transformation
- Verification of integration

### Pre-Cloning in *E. coli*

The cloning, that means the insertion of your part into the multiple cloning site of a vector, is done by normal ligations and *E. coli*. However, since the *B. subtilis* vectors are quite large, the cloning works best if only one insert is inserted. You could try to finish your construct in e.g. pSB1C3 and then clone it into the *B. subtilis* vector. For convenience, all vectors carry an RFP with promoter and terminator which is substituted by your insert during the ligation.

[
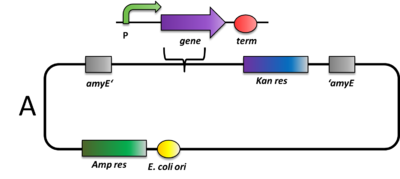
](http://2012.igem.org/File:LMU-Munich-Cloning.png)

All our vectors carry the *bla* gene that mediates Ampicillin resistance (100 µg/ml) in *E. coli*. Also, they all have two recombination sites for integration into the *B. subtilis* genome. In between those recombination sites, there is the multiple cloning site and a resistance marker for *B. subtilis*.

(Some vectors from other working groups do not carry an extra *E. coli* resistance, so the *B. subtilis* resistance is also used in *E. coli* but with lower antibiotic concentrations. There is also the possibility of single-crossover plasmids which do also work fine, but the vector can then easily cross-out again, so it is not stably integrated.)

### Linearisation before transformation in *B. subtilis*

[
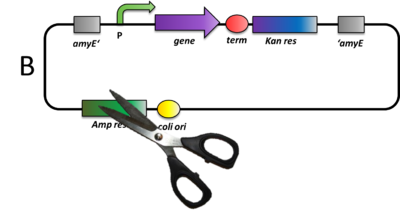
](http://2012.igem.org/File:LMU-Munich-integration-Picture2.png)

[
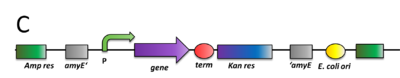
](http://2012.igem.org/File:LMU-Munich-integration-Picture3.png)

For integration ofs by double crossover, plasmids (if it is not replicative) have to be linearized before transformation. *B. subtilis* is naturally competent, takes up DNA fragments and integrates preferably linear fragment into its genome via double cross-over. The linearization of our plasmids can all be performed with *Sca*I which cuts only inside the *bla* gene. If that enzyme also cuts in your insert, please check for other single cutters outside of the area that is integrated into the *B. subtilis* genome. For the actual transformation of *B. subtilis*, please linearize 1-2 µg of your plasmid and then proceed with our [transformation protocol](http://2012.igem.org/wiki/images/4/41/LMU-Munich_2012_Transformation_of_Bacillus_subtilis.pdf).

### Verification of correct integration

Transformants are plated on selective media containing the appropriate antibiotic (resistance gene in between recombination sites). The obtained colonies then are tested for their insertion into the correct locus. Usually it is sufficient to test 4-8 colonies.

To test the insertion into the

- *amyE*-locus: *amyE* codes for an α-Amylase which degrades starch. Disruption of *amyE* disables *B. subtilis* of degrading starch. Starch is usually visualized by the starch-iodine reaction with Lugol's iodine that reveals a dark blue colour.

To test your transformants, streak the obtained colonies (and the WT as control) on a replica plate (with antibiotic) and on a starch plate and incubate overnight at 37°C. The next day, pour Lugol’s iodine on the plate so that it is covered with a thin film. Around colonies which can degrade starch (WT and wrong colonies), there should be a bright zone around the colony. Correct clones do not show this bright zone. (see also Figure 1)

| \| [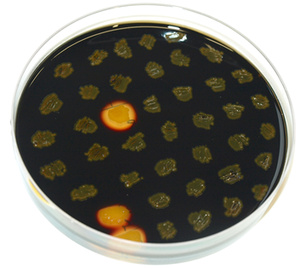](http://2012.igem.org/File:LMU-Munich-starchplate.JPG) \| \| --- \| \| \| Figure 1: Starch plate with *B. subtilis* streaked out colonies, covered with Lugol’s iodine. 3 strains can still hydrolyze starch, which can be seen by the bright surrounding area. The other clones have the insertion in the correct locus. \| \| --- \| \| |
| --- | --- | --- | --- |

- *thrC*-locus: *thrC* codes for the threonine synthase which performs an essential reaction for the production of the amino acid threonine. Disruption of that gene leads to threonine auxotrophy which can be tested for with minimal medium.Totest the transformants, streak the obtained colonies (and the WT as control) on a replica plate (with antibiotic) and on minimal medium without threonine and minimal medium with threonine (use the MNGE media, recipe see transformation protocol). Correct colonies should grow only on LB and minimal medium with threonine (see Figure 2).

| \| [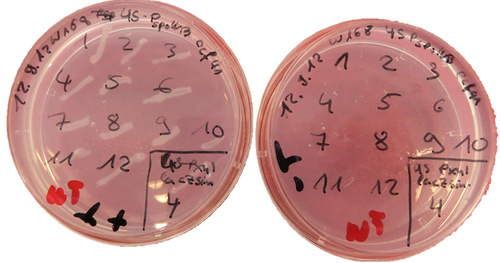](http://2012.igem.org/File:LMU-Munich-Thrplate.jpg) \| \| --- \| \| \| Figure 2: Agar-plates with MNGE-medium; left plate with threonine added, right plate without threonine. All colonies grow well on the left plate but not at all on the right plate. Those colonies all have the insertion in the correct locus. The colony in the lower right corner (4) is an exception. Is grows on both plates, indicating that the plasmid is not in the correct locus. \| \| --- \| \| |
| --- | --- | --- | --- |

- *sacA*-locus and *lacA*-locus: for those two loci, a colony PCR should be performed. The protocol can be found on our website. As shown below with an examplary locus, one of the primers of each pair is located on the genome, facing inwards, and the other one is located in between the recombination sites, facing outwards.
- For *sacA*: you can use the following primers:

up TM2505:CTGATTGGCATGGCGATTGC together with TM2506: ACAGCTCCAGATCCTCTACG as well as

down: TM2507: GTCGCTACCATTACCAGTTG together with TM2508: TCCAAACATTCCGGTGTTATC.

[
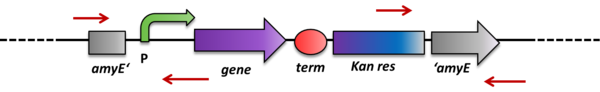
](http://2012.igem.org/File:LMU-Munich-Colony_pcr.png)

| \| [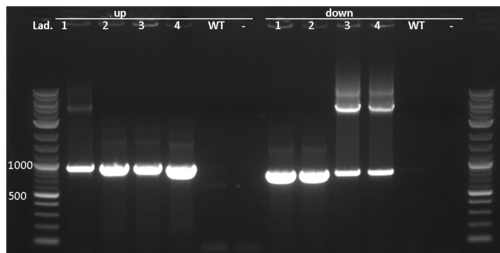](http://2012.igem.org/File:LMU-Munich-Gelfoto_colony_PCR.png) \| \| --- \| \| \| Figure 3: Colony PCR of pSB_Bs_3C-*luxABCDE*-P_lepA_ integrated into the *sacA*-locus. The expected bands are: up: 946 bp, down: 930 bp, none in WT and none with water as negative control. So all of the checked colonies have the insertion in the right locus. \| \| --- \| \| |
| --- | --- | --- | --- |

- for *lacA*, you can use the primers: TM2624: GAACGAAGGGCTAAGAGAAC

and TM2625:AAGCAGAAGGCCATCCTGAC (result: 650 bp) as well as TM2624 and TM2627: AAGAATCCGCCCATATCGAG (result: 3000 bp + length of construct). With colony PCR you can check any other locus.
